# Identification and Expression Analysis of the Complete Family of Zebrafish *pkd* Genes

**DOI:** 10.3389/fcell.2017.00005

**Published:** 2017-02-21

**Authors:** Samantha J. England, Paul C. Campbell, Santanu Banerjee, Annika J. Swanson, Katharine E. Lewis

**Affiliations:** Department of Biology, Syracuse UniversitySyracuse, NY, USA

**Keywords:** PKD, polycystin, TRP proteins, spinal cord, taste buds, node, dorsal forerunner cells, kidney

## Abstract

Polycystic kidney disease (PKD) proteins are trans-membrane proteins that have crucial roles in many aspects of vertebrate development and physiology, including the development of many organs as well as left–right patterning and taste. They can be divided into structurally-distinct PKD1-like and PKD2-like proteins and usually one PKD1-like protein forms a heteromeric polycystin complex with a PKD2-like protein. For example, PKD1 forms a complex with PKD2 and mutations in either of these proteins cause Autosomal Dominant Polycystic Kidney Disease (ADPKD), which is the most frequent potentially-lethal single-gene disorder in humans. Here, we identify the complete family of *pkd* genes in zebrafish and other teleosts. We describe the genomic locations and sequences of all seven genes: *pkd1, pkd1b, pkd1l1, pkd1l2a, pkd1l2b, pkd2*, and *pkd2l1*. *pkd1l2a/pkd1l2b* are likely to be ohnologs of *pkd1l2*, preserved from the whole genome duplication that occurred at the base of the teleosts. However, in contrast to mammals and cartilaginous and holostei fish, teleosts lack *pkd2l2*, and *pkdrej* genes, suggesting that these have been lost in the teleost lineage. In addition, teleost, and holostei fish have only a partial *pkd1l3* sequence, suggesting that this gene may be in the process of being lost in the ray-finned fish lineage. We also provide the first comprehensive description of the expression of zebrafish *pkd* genes during development. In most structures we detect expression of one *pkd1*-like gene and one *pkd2*-like gene, consistent with these genes encoding a heteromeric protein complex. For example, we found that *pkd2* and *pkd1l1* are expressed in Kupffer's vesicle and *pkd1* and *pkd2* are expressed in the developing pronephros. In the spinal cord, we show that *pkd1l2a* and *pkd2l1* are co-expressed in KA cells. We also identify potential co-expression of *pkd1b* and *pkd2* in the floor-plate. Interestingly, and in contrast to mouse, we observe expression of all seven *pkd* genes in regions that may correspond to taste receptors. Taken together, these results provide a crucial catalog of *pkd* genes in an important model system for elucidating cell and developmental processes and modeling human diseases and the most comprehensive analysis of embryonic *pkd* gene expression in any vertebrate.

## Introduction

Polycystic kidney disease (PKD) proteins are trans-membrane proteins that share a conserved polycystin-cation-channel domain, located in their last six trans-membrane domains. These proteins are crucially important for human health as they have essential functions in many aspects of vertebrate development and physiology (Delmas, 2004b; Venkatachalam and Montell, 2007; Zhou, 2009; Semmo et al., 2014). Most notably, this gene/protein family is named after PKD because mutations in either *PKD1* or *PKD2* account for all of the known forms of Autosomal Dominant Polycystic Kidney Disease (ADPKD), which is the most common genetic cause and the fourth most common cause of kidney failure. ADPKD affects one in 400–1000 individuals, across all ethnic groups, which also makes it the most frequent potentially-lethal single-gene disorder in humans (Dalgaard, 1957; Iglesias et al., 1983; Reeders et al., 1985; Levy and Feingold, 2000; Sutters and Germino, 2003; Zhou, 2009). In PKD, large epithelial-lined cysts develop and fill with fluid. This causes abnormally enlarged kidneys and the cysts compress normal renal tissue, destroying it and impairing normal kidney function. This usually results in chronic renal failure by middle age. In addition, cysts can also form in the liver, pancreas, spleen, ovaries, large bowel, brain, and heart and patients often have cardiovascular defects (Grantham, 1993; Wu and Somlo, 2000; Delmas, 2004b; Harris and Torres, 2009; Zhou, 2009; Cornec-Le Gall et al., 2013; Paul et al., 2014; Semmo et al., 2014). Mice heterozygous for a mutation in *Pkd2* also develop kidney cysts and renal failure and die as young adults (Wu and Somlo, 2000). In contrast, mice that have homozygous mutations in *Pkd2* or *Pkd1* die before birth, probably due to cardiac failure caused by incorrect heart development (Wu and Somlo, 2000; Boulter et al., 2001). In addition, these embryos have defects in their kidneys and pancreas (Lu et al., 1997, 2001; Kim et al., 2000; Wu et al., 2000; Boulter et al., 2001). *Pkd1* homozygous mutants also have skeletal defects (Boulter et al., 2001; Lu et al., 2001) and *Pkd2* and *Pkd1l1* are required for left-right patterning/asymmetry and the correct localization of several organs (Pennekamp et al., 2002; McGrath et al., 2003; Field et al., 2011; Kamura et al., 2011; Yoshiba et al., 2012; Yuan et al., 2015).

While less is known about the functions of the other *Pkd* genes, about 50% of mice homozygous for a mutation in *Pkd2l1* have heterotaxy (intestinal malrotation; Delling et al., 2013) and up-regulation of *Pkd1l2* in mouse causes profound neuromuscular defects (Mackenzie et al., 2009). *Pkdrej* is expressed in sperm suggesting that it may have a role in male fertility (Veldhuisen et al., 1999; Butscheid et al., 2006) and there is *in vitro* evidence that a complex of PKD1L3 and PKD2L1 may function as sour-taste receptors (Huang et al., 2006; Ishimaru et al., 2006), although this may not be the case *in vivo*, at least in mouse, as analysis of a mouse *Pkd1l3* mutant found no significant defect in taste reception (Nelson et al., 2010).

In humans and mouse there are eight *PKD* genes: *PKDREJ, PKD1, PKD1L1, PKD1L2, PKD1L3, PKD2, PKD2L1*, and *PKD2L2* (Zhou, 2009). These can be divided into two main sub-groups. *PKD1-like* (also called *polycystin-1*) genes (*PKDREJ, PKD1, PKD1L1, PKD1L2, PKD1L3*) are large multi-exon genes, encoding proteins of 1700–4300 amino acids. For example, human and mouse *PKD1* each have 46 exons encoding about 4300 amino acids (Li et al., 2003). PKD1-like proteins have 11 trans-membrane domains, a large extracellular N-terminal domain and a short intracellular C-terminal tail with a G-protein binding site and, in some cases, a coiled-coil domain. The N-terminal domain typically contains several repeats of an Ig-fold-containing domain called the PKD domain, a lipoxygenase homology/polycystin-lipoxygenase-atoxin PLAT/LH2 domain, a G-protein-coupled receptor proteolytic site (GPS) and a receptor egg jelly (REJ) domain (Delmas, 2004b; Zhou, 2009; Hofherr and Kottgen, 2011; Semmo et al., 2014). In contrast, PKD2-like (also called TRPP) proteins (PKD2, PKD2L1 and PKD2L2) are shorter, <1000 amino acids in each case (Veldhuisen et al., 1999; Li et al., 2003; Zhou, 2009; Semmo et al., 2014). These proteins are non-selective cation-channel proteins with six trans-membrane domains, an intracellular N-terminal domain and an intracellular C-terminal domain that sometimes contains a coiled-coil domain (Delmas, 2004b; Venkatachalam and Montell, 2007; Zhou, 2009; Hofherr and Kottgen, 2011; Semmo et al., 2014). PKD2-like proteins are part of the transient receptor potential (TRP) channel superfamily (Delmas, 2004b; Ishimaru et al., 2006; Owsianik et al., 2006; Ramsey et al., 2006; Venkatachalam and Montell, 2007; Zhou, 2009; Nilius and Owsianik, 2011; Semmo et al., 2014). TRP proteins all have six trans-membrane domains with a pore domain between the 5th and 6th domains and they have crucial roles in many different sensory functions including detection of mechanical, chemical, and thermal stimuli (Montell, 2005; Owsianik et al., 2006; Ramsey et al., 2006; Venkatachalam and Montell, 2007; Damann et al., 2008; Nilius and Owsianik, 2011; Venkatachalam et al., 2014). Interestingly, it has been proposed that the PKD2-like/TRPP proteins may be the most evolutionary ancient of all of the TRP proteins as they are found not just in vertebrates and invertebrates but also in yeast (Palmer et al., 2005; Venkatachalam and Montell, 2007; Semmo et al., 2014).

PKD1-like and PKD2-like proteins form heteromeric polycystin-receptor-channel complexes and, in at least some cases, physical interaction between these proteins is crucial for correct membrane localization of the resulting complex as well as correct physiological function (Li et al., 2003; Murakami et al., 2005; Ishimaru et al., 2006; Giamarchi et al., 2010; Field et al., 2011; Semmo et al., 2014). Consistent with this, mutations in partner proteins usually produce almost identical phenotypes both in humans and model organisms (e.g., Barr and Sternberg, 1999; Sutters and Germino, 2003; Field et al., 2011). For example, PKD2 and PKD1L1 physically interact and mutations in either of these genes cause defects in left-right patterning (Field et al., 2011). Similarly, PKD1 complexes with PKD2 and mutations in either of these genes cause ADPKD (Qian et al., 1997; Tsiokas et al., 1997; Yu et al., 2009; Zhu et al., 2011).

PKD heteromeric complexes are thought to form receptor-mediated non-selective cation-channels that are often located in primary cilia. For example, PKD1 and PKD2 form a non-selective cation-channel located in primary cilia of renal epithelial cells, that is thought to transduce extracellular stimuli such as fluid flow, possibly through altering general intracellular calcium signals (Hanaoka et al., 2000; Nauli et al., 2003; Delmas, 2004a; Delmas et al., 2004; Zhou, 2009 although see Delling et al., 2016, which challenges this model) or by altering local ciliary calcium concentrations (Delling et al., 2013; DeCaen et al., 2016). Similarly, PKD2 and PKD1L1 may form a calcium channel in the primary cilia of cells in the node, which could help establish left/right asymmetry during early stages of development, by sensing and transducing the left-biased signal (Pennekamp et al., 2002; McGrath et al., 2003; Field et al., 2011; Kamura et al., 2011; Yoshiba et al., 2012, although again see Delling et al., 2016, which challenges this model).

Given the importance of *PKD* genes to many different aspects of vertebrate embryonic development and physiology, it is crucial that we know where all of these genes are expressed. This may help us to identify other potential functions and interacting partners for this family of proteins. Zebrafish is a powerful model system for elucidating developmental and cell biological processes and for modeling and studying human diseases (e.g., Hostetter et al., 2003; Huang et al., 2014; Avagyan and Zon, 2016; Bournele and Beis, 2016; Brown et al., 2016; Carneiro et al., 2016; Griffin et al., 2016; Harrison et al., 2016; Kozol et al., 2016; Myllymaki et al., 2016; Poureetezadi and Wingert, 2016; Song et al., 2016; Wager et al., 2016; Wojciechowska et al., 2016; Zon, 2016). Consistent with this, all of the evidence so far suggests that zebrafish *pkd* genes function in ways that are highly conserved with their mammalian orthologs. For example, *pkd2* expression is enriched in the developing zebrafish pronephros (Bisgrove et al., 2005; Schottenfeld et al., 2007), Pkd2 protein is present in zebrafish kidney epithelial cells (Obara et al., 2006), and knock-down of *pkd2* function causes cyst formation in the zebrafish pronephros (Sun et al., 2004; Obara et al., 2006; Streets et al., 2006; Fu et al., 2008; Chang et al., 2011; Arif Pavel et al., 2016). In addition, *pkd2* is expressed in Kupffer's vesicle (KV) during early zebrafish embryogenesis (Bisgrove et al., 2005; Schottenfeld et al., 2007; Roxo-Rosa et al., 2015). The KV is a transient organ that forms during late gastrulation stages from dorsal forerunner cells that coalesce near the caudal end of the zebrafish embryo, and it is required to set up left/right asymmetry (Essner et al., 2005; Kramer-Zucker et al., 2005; Sampaio et al., 2014; Smith et al., 2014). Consistent with this, knock-down of Pkd2 function in zebrafish causes disturbed left-right patterning/asymmetry and randomization of heart and gut looping (Bisgrove et al., 2005; Schottenfeld et al., 2007) and zebrafish *pkd2* mutants have impaired cardiac function (Paavola et al., 2013). Similarly, knock-down of Pkd1 causes cyst formation in the liver (Tietz Bogert et al., 2013). In addition, studies in zebrafish have identified novel functions for Pkd proteins, such as helping to integrate mechanosensory feedback into locomotor neural circuits (Bohm et al., 2016).

Despite the importance of *pkd* genes, when we started this study only three *pkd* genes had been described in zebrafish, *pkd1, pkd1b*, and *pkd2*, although analyses of *pkd2l1* were also published more recently (Sun et al., 2004; Bisgrove et al., 2005; Obara et al., 2006; Streets et al., 2006; Schottenfeld et al., 2007; Feng et al., 2008; Fu et al., 2008; Francescatto et al., 2010; Giamarchi et al., 2010; Hurd et al., 2010; Mangos et al., 2010; Chang et al., 2011; Fogelgren et al., 2011; Merrick et al., 2012; Graham et al., 2013; Paavola et al., 2013; Tietz Bogert et al., 2013; Coxam et al., 2014; Djenoune et al., 2014; Fidelin and Wyart, 2014; Goetz et al., 2014; Quan et al., 2015; Roxo-Rosa et al., 2015; Yuan et al., 2015; Arif Pavel et al., 2016; Bohm et al., 2016). It was also unclear whether zebrafish have duplicate copies (ohnologs) of any of the *Pkd* genes found in mammals, from the genome duplication event at the base of the teleosts (Amores et al., 1998; Postlethwait et al., 1998; Force et al., 1999; Postlethwait, 2007). Therefore, we decided to identify the full complement of zebrafish *pkd* genes. Using bioinformatics and RT-PCR-based cloning we have identified seven zebrafish *pkd* genes: *pkd1, pkd1b, pkd1l1, pkd1l2a, pkd1l2b, pkd2*, and *pkd2l1*. We have also identified what may be a remnant of *pkd1l3* that lacks the polycystin-cation-channel domain sequence that is conserved in all other *pkd* genes. Therefore, we do not consider this a bona-fide *pkd* gene. In this paper we identify the sequences and genomic locations of all of these genes. We also confirm that no additional *pkd* genes exist in three other teleosts: medaka, stickleback or green spotted pufferfish. In addition, we describe the expression of each of the seven zebrafish *pkd* genes during embryonic and larval development. Taken together, we provide the first description of the complete family of zebrafish *pkd* genes and the most comprehensive analysis of embryonic *pkd* gene expression in any vertebrate.

## Materials and methods

### Ethics approval

All zebrafish experiments in this research were approved by the Syracuse University IACUC committee.

### Zebrafish husbandry and fish lines

Zebrafish (*Danio rerio*) were maintained on a 14-h light/10-h dark cycle at 28.5°C. Embryos were obtained from natural paired and/or grouped spawnings of wild-type (WT; AB, TL, or AB/TL hybrid) or *mindbomb* (*mib*^*ta*52*b*^; Jiang et al., 1996) or *Tg(*−*8.1gata1:gata1-EGFP)* (Kobayashi et al., 2001) fish. Embryos were staged in hours post fertilization at 28.5°C (h) or days post fertilization (dpf) according to Kimmel et al. (1995).

### Identification of *pkd* genes

Initially we searched NCBI, http://www.ZFIN.org and Ensembl for zebrafish *pkd* genes. We then blasted nucleotide sequences for these genes against the zebrafish genome using Tblastn on Ensembl http://www.ensembl.org/Danio_rerio/Tools/Blast?db=core. We identified polycystin-cation-channel domains and performed a Tblastn with these peptide sequences using default parameters at NCBI (http://blast.ncbi.nlm.nih.gov/Blast.cgi?PROGRAM=tblastn&PAGE_TYPE=BlastSearch&BLAST_SPEC=OGP__7955__9557&LINK_LOC=blasttab&LAST_PAGE=blastn).

Protein sequences were obtained from mapped mRNA transcripts using the Translate tool at the ExPASy Bioinformatics Resource Portal: http://web.expasy.org/translate/. To compare and analyze protein structures, protein domains were identified by searching against the Pfam protein database at EMBL-EBI: http://pfam.xfam.org (Finn et al., 2015).

To amplify *in situ* hybridization probe templates and confirm particular open reading frames we created zebrafish cDNA from 27 h WT zebrafish embryos. Total RNA was extracted by homogenizing 50–100 mg of embryos in 1 mL of TRIzol reagent (Ambion, 15596-026). RNA integrity (2:1 ratio of 28S:18S rRNA bands) and quality (A260/A280 ratio of ~2.0) was confirmed using agarose gel electrophoresis and spectrophotometry respectively. cDNA was synthesized using Bio-Rad iScript Reverse Transcription Supermix kit (Bio-Rad, 170-8891).

To map and confirm open reading frames, PCRs were performed using 5 μl of cDNA template in a 50 μl reaction, with Phusion High-Fidelity DNA Polymerase (NEB, M0530L) and mapping primers listed in Supplementary Table 1. Reaction conditions were 98.0°C for 30 s, followed by 30 cycles of: 98.0°C for 10 s, Annealing (see Supplementary Table 1 for temperatures) for 20 s, and extension at 72.0°C (see Supplementary Table 1 for extension times). A final extension step was performed for 5 min at 72.0°C.

For *pkd1, pkd1l2a*, and *pkd1l2b* we also performed inverse PCR to identify missing 5′ sequence, as described in Lewis et al. (1999), with the following modifications. One microgram of total RNA extracted from 27 h WT zebrafish embryos (see above) was incubated with 10 μM of gene specific primer (see Supplementary Table 2) and 1 mM each of dNTPs in a final volume of 10 μl for 5 min at 65°C. Two-hundred units of M-MuLV Reverse Transcriptase (NEB, M0253S) and eight units of Protector RNase Inhibitor (Roche, 03335399001) were then added and first strand cDNA synthesized by incubating for 1 h at 42°C. Second strand cDNA synthesis was performed immediately as described in Lewis et al. (1999), but the reaction was incubated for 4 h at 14°C, followed by 10 min at 70°C, before adding five units of T4 DNA Polymerase (NEB, M0203S) and incubating for 10 min at 37°C. Circularization was performed as described in Lewis et al. (1999), with the exception that RNA ligase was omitted and purification was performed using Amicon Ultra-0.5 Centrifugal Filter Units with Ultracel-30 Membrane (Millipore Sigma, UFC503024). Reaction products were diluted to a final volume of 500 μl using nuclease-free water and filtered by centrifuging for 10 min at 14000 × g, before eluting by inverting filter and centrifuging for 2 min at 1000 × g. Five microliters of purified, circularized product was used in a 50 μl PCR with Phusion High-Fidelity DNA Polymerase (NEB, M0530L). Reaction conditions were: 98.0°C for 30 s, followed by 35 cycles of: 98.0°C for 10 s, Annealing—(see Supplementary Table 1 for temperatures) for 20 s and Extension (see Supplementary Table 1 for extension times) at 72.0°C. A final extension step was performed for 5 min at 72.0°C.

For *pkd1* and *pkd1l2b*, nested PCR was performed. The first round of PCR was performed as described above, using the respective Nested_Set 1 primers (Supplementary Table 1). This product was diluted 1:10 in nuclease-free water and 2.5 μl of that dilution used as a template in the second round PCR, using Nested_Set 2 primers (Supplementary Table 1).

In all cases, PCR products were verified on a 1% agarose TAE gel and then purified using EZ-10 Spin Column PCR Products Purification kit (Bio Basic Inc, BS664). Purified PCR products were sequenced using the PCR primers (Supplementary Table 1) to prime the reactions and the resulting sequences blasted against zebrafish genome assembly GRCz10 using Tblastn and default parameters on Ensembl (http://www.ensembl.org/Danio_rerio/Tools/Blast?db=core).

Our mapped mRNA transcript sequences for zebrafish *pkd1, pkd1l2a*, and *pkd1l2b* have been submitted to NCBI [KY074550 (*pkd1*), KY074551 (*pkd1l2a*), and KY074552 (*pkd1l2b*)].

### Phylogenetic analyses

The peptide sequence for the polycystin-cation-channel domain was identified using Pfam (http://pfam.xfam.org/; Finn et al., 2015) and isolated, when present, from all of the *pkd* genes in zebrafish (*Danio rerio, dre*), green spotted pufferfish (*Tetraodon nigroviridis, tni*), medaka (*Oryzias latipes, ola*), stickleback (*Gasterosteus aculeatus, gac*), spotted gar (*Lepisosteus oculatus, loc*), elephant shark (*Callorhinchus milii, cmi*), fly (*Drosophila melanogaster, dme*), human (*Homo sapiens, hsa*), and mouse (*Mus musculus, mmu*). The following genome assemblies were used: zebrafish—GRCz10, green spotted pufferfish—TETRAODON 8.0, medaka—HdrR and stickleback—BROAD S1. For spotted gar, elephant shark, fly, human, and mouse proteins, the polycystin-cation-channel domain sequences were isolated from the longest protein isoforms available in Ensembl genomes LepOcu1 (GCA_000242695.1), ESHARK1, BDGP6 (GCA_000001215.4), GRCh38.p7 (GCA_000001405.22), and GRCm38.p4 (GCA_000001635.6), respectively. Protein sequence alignment was performed using Clustal Omega server at EMBL-EBI (Version 1.2.3) and default parameters: http://www.ebi.ac.uk/Tools/msa/clustalo/ (Goujon et al., 2010; Sievers et al., 2011; McWilliam et al., 2013). Phylogenetic trees for the PKD1-like and PKD2-like families were generated using regions of the polycystin-cation-channel domain contained in all of the proteins (see Supplementary Figures 1, 2). We used both the neighbor-joining (NJ) method [plotted using Phylodendron software (version 0.8d) http://iubio.bio.indiana.edu/treeapp/treeprint-form.html] and the maximum likelihood method, implementing a WAG substitution model, performed using PhyML (v3.1/3.0 aLRT) accessed at the Phylogeny.Fr web interface (http://www.phylogeny.fr/index.cgi; Dereeper et al., 2008, 2010). The maximum likelihood analyses are presented here (**Figure 3**).

### Syntenic analyses

Having identified genomic loci of teleost *pkd* genes through Tblastn analysis (see above), we compared these to *PKD* loci in human and mouse genomes using location-based displays in Ensembl to identify any conserved synteny.

### *In situ* hybridization and immunohistochemistry

**Figure 1:**
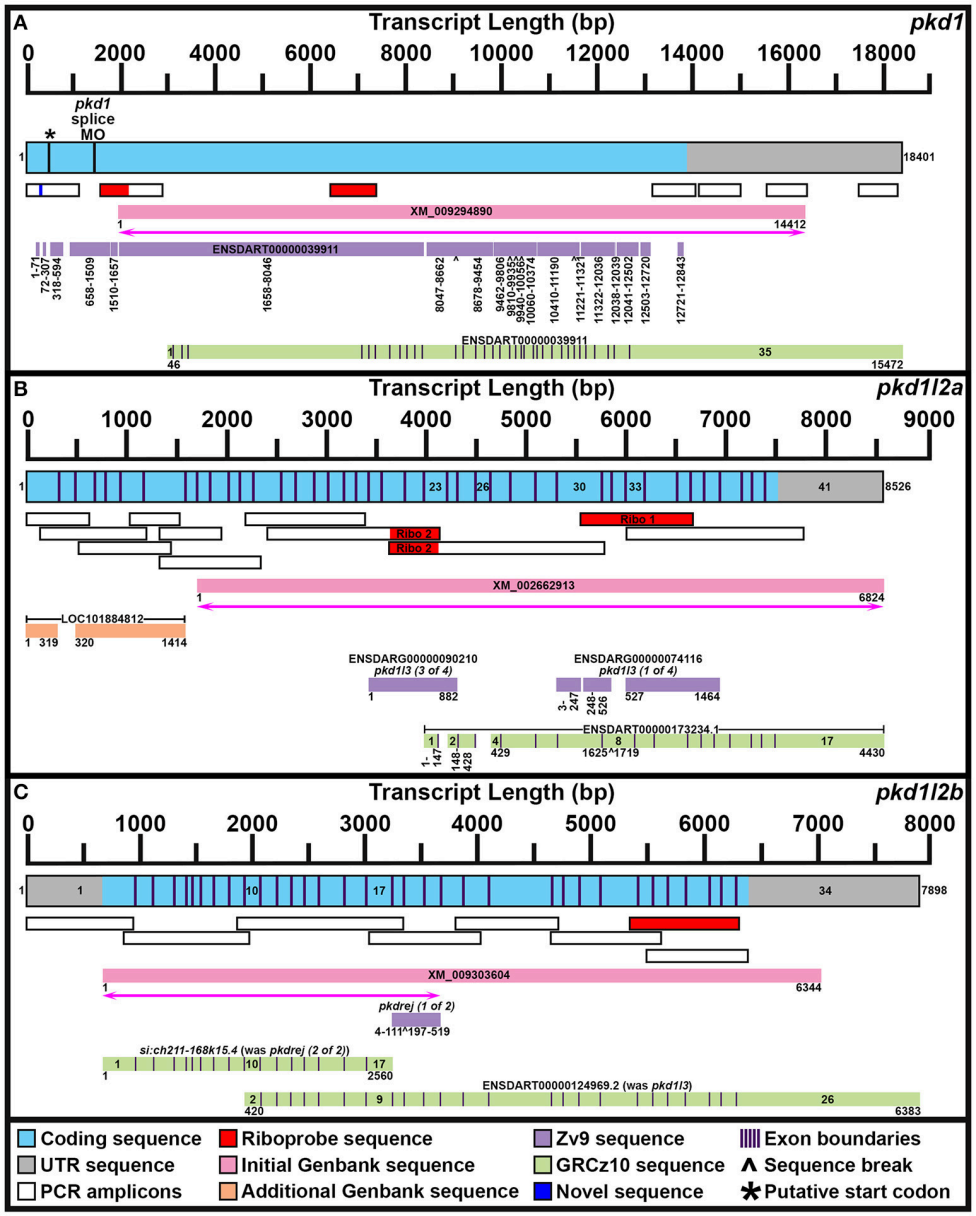
**Mapping ***pkd1***, ***pkd1l2a***, and ***pkd1l2b*** mRNA Transcripts**. Summary of mRNA transcript mapping results for *pkd1* **(A)**, *pkd1l2a* **(B)**, and *pkd1l2b* **(C)**. Approximate length in base pairs (bp) is indicated by scale at top of each panel. Mapped transcripts are shown in next row of each panel. Coding sequence is blue and UTR is gray. Numbers flanking these mapped transcripts indicate nucleotide positions. Black vertical lines (coding sequence box, **A**) indicate putative start codon, and morpholino sequence positions. Purple vertical lines (coding sequence boxes, **A–C**) indicate exon boundaries, where known. Mapped PCR amplicons generated in this study are indicated with white boxes. Red indicates riboprobe sequences used in this study. Dark blue indicates novel sequence identified in this study but not currently present in Ensembl GRCz10 genome. Genbank reference sequences used at beginning of this study are shown as pink boxes. Magenta lines with double arrows beneath these indicate regions of sequence homology identified at start of this project. Genbank reference sequences identified during this study are shown as orange boxes. Ensembl Zv9 transcript sequences are shown as lilac boxes. Ensembl GRCz10 transcript sequences are shown as green boxes. Numbers beneath sequences show nucleotide positions. ∧, break in aligned sequence. Thin purple vertical lines in green boxes indicate exon boundaries, where known. Key exons for interpreting mapping results are numbered. **(A)** Our newly mapped *pkd1* transcript contains all but 173 bases of the older Zv9 ENSDART00000039911 transcript (lilac boxes) as well as all but the first 45 nucleotides of the current GRCz10 ENSDART00000039911 transcript (green boxes). We have also identified additional 5′ sequence and missing regions of coding sequence. The GRCz10 ENSDART00000039911 transcript corresponds to nucleotides 2975–18401 of our mapped transcript. The Zv9 ENSDART00000039911 transcript corresponds to nucleotides 218–13798 of our mapped transcript but contains some gaps (nucleotides 292–357, 430–597, 875–925, 1778–1786, 1935–1973, 8363–8410, 9804–9811, 10715–10725, 11608–11640, 12356, 12359–12389, 12852–12890, and 13109–13675 of our mapped transcript). Inverse PCR identified 5′ transcript sequence along with a novel stretch of nucleotides (292–357) absent from GRCz10 Ensembl genome (shown in dark blue). Nucleotides 2659–12720 of the Zv9 transcript are almost 100% identical to nucleotides 46–10179 of the GRCz10 transcript and these regions align with nucleotides 2975–13108 of our mapped transcript. Nucleotides 10180–10746 of the GRCz10 transcript share no homology with the Zv9 transcript, but correspond to nucleotides 13109–13675 of our mapped transcript. Nucleotides 12721–12843 of the Zv9 transcript share 100% homology with nucleotides 10747–10869 of the GRCz10 transcript and correspond to nucleotides 13676–13798 of our mapped transcript. The coding sequence of the GRCz10 transcript terminates 30 nucleotides downstream of the Zv9 transcript and is followed by 4573 bp of unique 3′ UTR sequence. Using RT-PCR we have confirmed that our mapped transcript utilizes the same stop codon and 3′ UTR sequence. Specifically, we have confirmed that nucleotides 10192–11136, 11173–12085, 12598–13484, and 14524–15376 of 3′ UTR sequence in the GRCz10 transcript are transcribed and map to nucleotides 13121–14065, 14102–15014, 15527–16413, and 17453–18395 of our mapped transcript, respectively. Our inverse PCR revealed 217 nucleotides of coding sequence upstream of the Zv9 transcript and 66 nucleotides of novel coding sequence between nucleotides 71 and 72 of the Zv9 transcript. In total, this produces a 18401 bp transcript that encodes a 4608 amino acid protein and we have deposited this sequence in NCBI (NCBI accession number KY074550). This sequence lacks a start methionine. ^*^indicates in-frame methionine at 527–529 nucleotides. However, if this is the start codon, the resulting protein would lack the leucine rich repeat domain, encoded by the 175 amino acids in-frame upstream of this methionine, that is present in mouse, human and stickleback PKD1. There is a putative in-frame start codon a further 54 nucleotides (18 amino acids) upstream of our present transcript, which we think is more likely to be the true start codon. The location of the splice-blocking morpholino sequence used by Mangos et al. (2010) that resulted in kidney cysts in some animals is also indicated (nucleotides 1187–1197 of the Zv9 transcript). **(B)** Our current transcript for *pkd1l2a* encompasses both *LOC101884812* and XM_002662913 and contains additional exons not present in either of these sequences. The start of the current ENSDART00000173234.1 transcript coincides with the start of exon 23 in our longer transcript, but the first exon of ENSDART00000173234.1 is shorter than exon 23 in our transcript. Exons 2–3, 4–7, and 9–17 of ENSDART00000173234.1 are identical to exons 24–25, 27–30, and 33–41 of our transcript. Exon 26 of our transcript is absent in ENSDART00000173234.1 and exons 31–32 and intron 31–32 exist as a single exon, exon 8, in ENSDART00000173234.1. **(C)** Our current transcript for *pkd1l2b* contains both the *si:ch211-168k15.4* and ENSDARG00000101214 (ENSDART00000124969.2) sequences, utilizing a start codon 4 bases upstream of exon 1 in the current *si:ch211-168k15.4* annotation, and transitioning between exon 16 of *si:ch211-168k15.4* immediately into exon 9 of ENSDART00000124969.2. Nucleotides 683–7026 of our new 7898 bp mRNA transcript align perfectly with the original XM_009303604 6344 bp reference sequence. The start codon was identified in this study along with novel 5′ UTR sequence.

**Figure 2:**
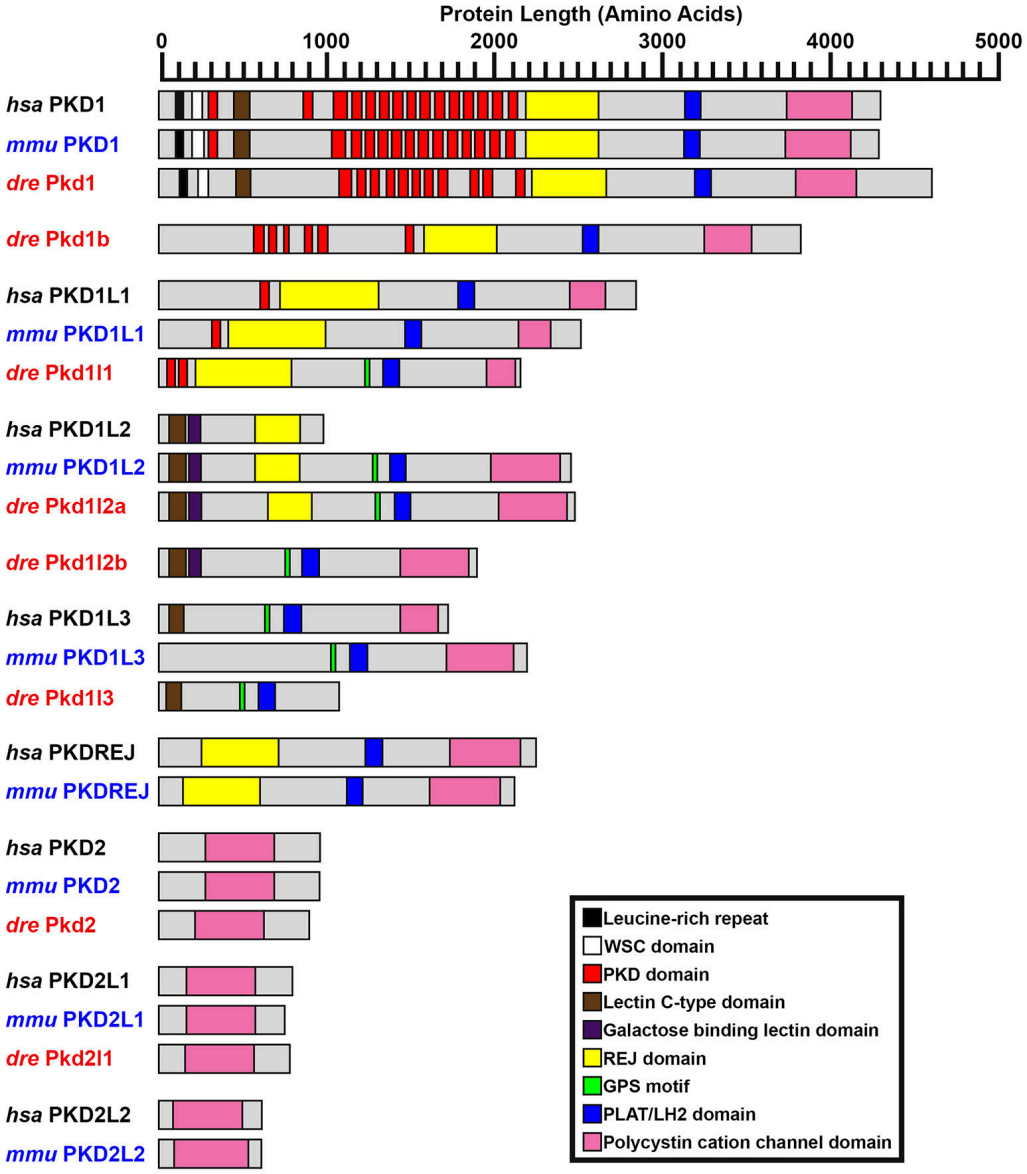
**PKD protein domains**. Schematics of protein domains identified in all eight human (*Homo sapiens, hsa*) and mouse (*Mus musculus, mmu*) and seven zebrafish (*Danio rerio, dre*) PKD proteins. The zebrafish putative partial *pkd1l3* ortholog is also shown. Approximate protein length is indicated by scale at top. Where multiple transcripts exist in Ensembl, the longest protein isoform is shown. In all three species, PKD1 is the longest PKD protein and the only protein to contain a leucine-rich repeat and carbohydrate-binding WSC domain in the amino-terminus. Pkd1b is not present in mammals. Zebrafish Pkd1b resembles Pkd1 with multiple PKD domain repeats in the amino-terminus and REJ, PLAT/LH2, and polycystin-cation-channel domains in the carboxy-terminus. In all three species, PKD1L1 contains a shorter polycystin-cation-channel domain, approximately half the size of that in other Pkd proteins. Unlike mammals, zebrafish Pkd1l1 also contains a GPS motif upstream of the PLAT/LH2 domain. The 5′ coding sequence of mouse *Pkd1l1* gene is presently incomplete. PKD1L2 is unusual in humans in that, according to information on Ensembl, longer transcripts represent polymorphic pseudogenes that have acquired mutations, preventing them from being expressed as functional proteins. As a result, the current version of human PKD1L2 is half the size of mouse and zebrafish Pkd1l2 and lacks the polycystin-cation-channel domain characteristic of PKD proteins. If this is correct, then this suggests that human *PKD1L2* is no longer a bona-fide *PKD* gene. Mouse PKD1L2 and Zebrafish Pkd1l2a and Pkd1l2b have identical domain structures, with the exception that Pkd1l2b lacks the REJ domain. PKD1L3 protein structure differs slightly between mammals. Human PKD1L3 has a Lectin C-type domain in the amino-terminus and mouse PKD1L3 does not. In addition, the polycystin-cation-channel domain in mouse PKD1L3 is 411 amino acids long, compared to only 237 amino acids in human PKD1L3. We have identified a putative partial *pkd1l3* ortholog in zebrafish, but the sequence lacks the polycystin-cation-channel domain, so we do not consider it a bona-fide *pkd* gene. Pkdrej and Pkd2l2 are not present in zebrafish. The only currently identified domain in PKD2, PKD2L1, and PKD2L2 proteins is the polycystin-cation-channel domain.

Embryos were fixed in 4% paraformaldehyde and single *in situ* hybridization or fluorescent *in situ* hybridization plus immunohistochemistry experiments were performed as previously described (Concordet et al., 1996; Batista et al., 2008). Embryos older than 24 h were often incubated in 0.003% 1-phenyl-2-thiourea (PTU) to prevent pigment formation. For fluorescent *in situ* hybridization + immunohistochemistry, after detection of the *in situ* hybridization reaction using TSA Kit #5, with HRP, Goat anti-mouse IgG and Alexa Fluor 594 Tyramide (ThermoFisher Scientific, T20915), embryos were washed 8 × 15 min in PBST and incubated in Image-iT FX Signal Enhancer (ThermoFisher Scientific, I36933) for 30 min at room temperature. Immunohistochemistry was performed using a chicken polyclonal anti-GFP primary antibody (Abcam, Ab13970, 1:500) and a Goat anti-chicken IgY (H+L), Alexa Fluor 488 secondary antibody (ThermoFisher Scientific, A-11039, 1:1000). Probes for *in situ* hybridization experiments were prepared using PCR-based DNA templates from 27 h cDNA, made as described above, and primers listed in Supplementary Table 3. Primers for all zebrafish *pkd* genes, except *pkd1* Primer Set 1, were designed using the following parameter ranges: nucleotide length—21 bases (minimum)-28 bases (maximum), tm—58°C (minimum)−65°C (maximum) and GC content—45% (minimum)-60% (maximum) with Primer3 web version 4.0.0 at http://bioinfo.ut.ee/primer3/ (Koressaar and Remm, 2007; Untergasser et al., 2012). All reverse primers include the sequence for the T3 RNA Polymerase minimal promoter: ATTAACCCTCACTAAAGGGA. This sequence is shown in bold and underlined in the reverse primers listed in Supplementary Table 3. To avoid cross-reactivity, whenever possible, riboprobes were designed against 3′ UTR or coding sequence lacking all of the conserved protein domains shown in **Figure 2**. *pkd1* Set 1 primers used to make the zebrafish *pkd1* riboprobe were identical to those described by Coxam et al. (2014). These primers generated a 580 bp PCR product and the resulting RNA probe revealed specific embryonic expression. However, this region of the *pkd1* transcript was no longer included in the annotation of the *pkd1* gene in Ensembl GRCz10 (Figure 1A). Therefore, we also generated an alternative *pkd1 in situ* probe that binds 3′ to the Coxam riboprobe, in a region included in the newer Ensembl transcript (*pkd1* Set 2 primers—see Supplementary Table 3, Figure 1A). This probe produced identical, albeit weaker, expression to the first (Coxam) riboprobe (data not shown). The stronger Coxam riboprobe was therefore used throughout this study.

To confirm genomic structure of *pkd1l2a*, two separate riboprobes were generated and tested (Supplementary Table 3, Figure 1B). These were generated against two adjacent genes that have been retired in Ensembl GRCz10 but that we show here encompass different parts of *pkd1l2a*. The *pkd1l2a* riboprobe generated with primer Set 1 is the most 3′ of the two probes. It was designed against ENSDARG00000074116 and it partially overlaps the current Ensembl *pkd1l2a* transcript. In contrast, the *pkd1l2a* riboprobe generated with primer Set 2 was designed against ENSDARG00000090210 and is immediately 5′ to the current Ensembl *pkd1l2a* transcript. Whilst both probes produced the same expression patterns, the latter probe was weaker in putative taste buds and therefore the probe designed against ENSDARG00000074116 (primer set 1) was used for all of the studies in this paper.

Each 50 μL probe reaction PCR contained 5 μL cDNA and one unit of Phusion High-Fidelity DNA Polymerase (NEB, M0530L). PCR conditions were: 94°C for 3 min followed by 35 cycles of 94°C for 30 s, 56.5°C for 30 s, 72°C for 1.5 min and then a final extension step of 72°C for 10 min. PCR products were purified by phenol:chloroform extraction. *in situ* hybridization probes were made using 1 μg purified PCR product, T3 RNA Polymerase (Roche, 11031171001) and DIG RNA Labeling Mix (Roche, 11277073910).

### Imaging

Embryos 24 h and older were deyolked in 70% glycerol/30% sterile water using mounting pins. For lateral and dorsal views of the embryo, whole embryos were mounted in 70% glycerol in coverslip sandwiches (24 × 60 mm coverslips; VWR, 48393-106), with 2–4 coverslips (22 × 22 mm; VWR, 16004-094) on either side of the sample to avoid sample compression. For ventral views of putative taste receptors, the trunk was dissected with a razor blade and the head carefully inverted on to a 24 × 60 mm coverslip and a similar coverslip sandwich made. For lateral views of eyes, they were dissected from forebrain using mounting pins and mounted as for whole embryos, but using only 1–2 coverslips each side of the specimen. Cross-sections were cut by hand using a razor blade mounted in a 12 cm blade holder (World Precision Instruments, Cat. #14134). Differential interference contrast (DIC) pictures were taken using an AxioCam MRc5 camera mounted on a Zeiss Axio Imager M1 compound microscope. A Zeiss LSM 710 confocal microscope was used to image embryos mounted in DABCO (1,4-Diazabicyclo[2.2.2]octane, Sigma, D-2522, 2% w/v solution in 80% sterile glycerol) for fluorescent double-labeling experiments. Images were processed using Adobe Photoshop software (Adobe, Inc) and Image J software (Abràmoff et al., 2004).

### Cell counts and statistics

In all cases, cells counts are for both sides of a five-somite length of the spinal cord adjacent to somites 6–10. Values are an average of five embryos. Results were analyzed using the student's *t*-test; Error bars indicate standard error of the mean.

## Results

### Zebrafish have seven *pkd* genes

To establish the full complement of zebrafish *pkd* genes we initially searched several online resources. We found NCBI nucleotide reference sequences for six genes: XM_009294890 (called *pkd1*), XM_009297371 (called *pkd1l1*), XM_009303604 (called *pkd1l2*), XM_002662913 (called *pkd1l3*), DQ175629 (called *pkd2*), and XM_690312 (called *pkd2l1*) and an additional gene, called *pkd1b*, on the zebrafish database website, ZFIN (Note: some of these records have since been retired as a result of standard genome-annotation processing and our data suggest that some of these names are not correct). To identify additional potential *pkd* genes, we blasted each of these sequences against zebrafish genome assembly Ensembl Zv9 using Tblastn. We also performed a textual search for *pkd* genes on Ensembl. Using these methods, we identified 10 potential *pkd* genes [called at that time *pkd1, pkd1b, pkd1l3, pkd1l3 (1 of 4), pkd1l3 (2 of 4), pkd1l3 (3 of 4), pkdrej (1 of 2), pkdrej (2 of 2), pkd2*, and *pkd2l1*]. We examined each of these, in order to determine which of them were indeed bona-fide *pkd* genes. These analyses identified seven *pkd* genes as described below.

#### pkd1

When we commenced our bioinformatic analyses of zebrafish *pkd* genes, the reference sequence XM_009294890 aligned in Ensembl Zv9 with a 12843 bp transcript, ENSDART00000039911 (associated with gene ENSDARG00000030417 on chromosome 1) called *pkd1*, that lacked both start and stop codons (Figure 1A; Table 1). However, this annotation changed in the current genome assembly, GRCz10, which contains a revised 15472 bp ENSDART00000039911 transcript that lacks the first 2607 nucleotides present in the older transcript. Supporting the older 5′ sequence, Coxam et al. (2014) showed enriched expression in zebrafish trunk at late embryonic stages using an *in situ* hybridization riboprobe designed against nucleotides 1307–1838 of the older transcript. We have also amplified this region from zebrafish cDNA and, in our hands, a riboprobe designed against this region is strongly expressed in embryonic pronephros, consistent with *pkd1* expression in other animals (see expression analyses below). This expression is identical, although stronger, to the expression that we see when we use an alternative riboprobe designed against a more 3′ region included in the newer Ensembl transcript (see Section Materials and Methods and Figure 1A). In addition, Mangos et al. (2010) describe a splice-blocking *pkd1* morpholino aligned with the older transcript (Figure 1A), that induced kidney cysts in some animals, consistent with Pkd1 function in other animals. Taken together, these data suggest that the current Ensembl annotation is incorrect and that at least some *pkd1* transcripts include parts of the older upstream sequence.

**Table 1:** **Characterization of teleost ***pkd*** genes**.

**Species**	**Former gene names**	**Chromosome location**	**Ensembl annotation available/ZFIN ID available for zebrafish genes?**	**Ensembl annotation complete and/or accurate?**	**Transcript mapped in this study?**	**Transcript length (bp)**	**Transcript complete?**	**Protein length (amino acids)**	**Protein domains (Position shown in amino acids)**	**Percentage amino acid identity to zebrafish Pkd polycystin-cation-channel domains**	**Supporting evidence**
***pkd1***											
Zebrafish	*pkd1a*	1(–): 53597305–53745945	ENSDARG00000030417; ZFIN ID: ZDB-GENE-100707-1	No—5′ coding sequence incomplete	Yes	18401	No∧	4608	Leucine rich repeat (93–149); Carbohydrate binding WSC domain (201–273); Lectin C-type domain (454–551); PKD domains (1067–1151, 1175–1236, 1257–1317, 1343–1402, 1425–1491, 1516–1572, 1597–1657, 1682–1747, 1854–1914, 1933–1998, 2112–2174); REJ domain (2210–2662); PLAT/LH2 domain (3198–3304); Polycystin-cation-channel domain (3786–4156)	Pkd2 (aa 54–95, 48%); Pkd1l2a (aa 121–160, 40%); Pkd1l2b (aa 112–160, 41%); Pkd2l1 (aa 86–145, 38%)	Phylogeny, synteny, domain structure, expression, and morpholino data (see text).
Green spotted pufferfish	None	18(+): 2403952–2439321	ENSTNIG00000014075	Yes	No	13572	Yes	4523	Carbohydrate binding WSC domain (201–281); Lectin C-type domain (441–544); PKD domains (1065–1155, 1172–1237, 1262–1319, 1342–1400, 1429–1496, 1521–1579, 1602–1663, 1693–1761, 2130–2186); REJ domain (2224–2690); PLAT/LH2 domain (3230–3336); Polycystin-cation-channel domain (3920–4171)	Pkd1 (aa 1–371, 34%)	Phylogeny, synteny, and domain structure.
Medaka	*pkd1 (1 of 2)*	1(+): 31241283–31246625^*^	ENSORLG00000011636 (Novel)	No—5′ and 3′ coding sequence incomplete	No	1926	No	642	Polycystin-cation-channel domain (95–508)	Pkd1 (aa 135–331, 56%)	Phylogeny and synteny.
	*pkd1 (2 of 2)*	Scaffold1073 (+): 14296–55909^*^	ENSORLG00000019755	No—5′ and 3′ coding sequence incomplete	No	10200	No	3400	Carbohydrate binding WSC domain (114–187); Lectin C-type domain (342–442); PKD domains (204–257, 981–1072, 1089–1155, 1178–1236, 1259–1323, 1347–1411, 1451–1493, 1514–1579, 1612–1673, 1694–1754, 2029–2094); REJ domain (2131–2599); PLAT/LH2 domain (3146–3251)	**NO HITS** Lacks a polycystin-cation-channel domain.	Domain structure.
***pkd1***											
Stickleback	None	GroupIX(+): 14963056–15005610	ENSGACG00000018946	No—5′ and 3′ coding sequence incomplete	No	12849	No	4283	Leucine rich repeat (72–127); Carbohydrate binding WSC domain (179–254); PKD domains (289–341, 1037–1126, 1143–1208, 1235–1290, 1311–1377, 1404–1466, 1495–1550, 1573–1633, 1663–1732, 1839–1897, 1917–1983, 2096–2156); Lectin C-type domain (435–535); REJ domain (2194–2649); PLAT/LH2 domain (3158–3263); Polycystin-cation-channel domain (3754–4098)	Pkd1 (aa 8–74 and 135–371, 50%)	Phylogeny, synteny, and domain structure.
***pkd1b***											
Zebrafish	None	12(+): 21576321–21626795	ENSDARG00000033029; ZFIN ID: ZDB-GENE-100707-2	Possible additional 5′ coding sequence (see text)	No	11674	Yes	3827	PKD domains (543–613, 641–696, 735–775, 895–951, 968–1036, 1493–1552); REJ domain (1589–2031); PLAT/LH2 domain (2519–2624); Polycystin-cation-channel domain (3250–3542)	No significant hits other than with itself.	Phylogeny with other teleosts (see text).
Green spotted pufferfish	None	2(–): 4745387–4760923	ENSTNIG00000004457	No—5′ and 3′ coding sequence incomplete	No	10137	No	3379	PKD domains (210–296, 313–378, 406–467, 486–550, 645–702, 989–1049, 1249–1311); REJ domain (1346–1787); PLAT/LH2 domain (2293–2397); Polycystin-cation-channel domain (2980–3242)	Pkd1b (aa 300–367, 57%)	Phylogeny and domain structure. Synteny with Stickleback *pkd1b* gene.
Medaka	**NO** ***pkd1b*** **ORTHOLOG**. Tblastn against the Medaka genome with the full-length zebrafish Pkd1b protein sequence revealed homology with *pkd1* on Scaffold1073 and the novel *pkd1* gene on chromosome 1, also some partial homology with *pkd1l2b*, but no additional *pkd* genes were detected.									**NO HITS**	Not applicable.
Stickleback	*pkd1b*	GroupV(–): 6686030–6695058	ENSGACG00000005441 (Novel)	No—5′ and 3′ coding sequence incomplete	No	4044	No	1348	REJ domain (9–211); PLAT/LH2 domain (718–822)	**NO HITS^¥^**	Synteny with Green spotted pufferfish *pkd1b* gene.
***pkd1l1***											
Zebrafish	*pkd1l3 (2 of 4)*	24(+): 17140489–17184898	ENSDARG00000099162 (*BX005392.1*); ZFIN ID: None available	No—3′ coding sequence incomplete	No	6486	Yes (see text).	2162	PKD domains (16–73, 99–159); REJ domain (200–781); GPS motif (1213–1251); PLAT/LH2 domain (1322–1251); Polycystin-cation-channel domain (1960–2140)	Pkd1l2a (aa 4–49, 37%); Pkd2 (aa 31–113, 33%); Pkd2l1 (aa 31–107, 30%)	Phylogeny. Synteny and expression data with other teleosts. Domain structure (see text).
***pkd1l1***											
Green spotted pufferfish	None	6(+): 2144742–2156425	ENSTNIG00000000844 (Novel)	No—5′ and 3′ coding sequence incomplete	No	6459	No	2153	PKD domains (12–74, 98–158); REJ domain (203–786); PLAT/LH2 domain (1293–1411); Polycystin-cation-channel domain (1925–2153)	Pkd1l1 (aa 3–67, 43%); Pkd2 (aa 132–215, 40%); Pkd2l1 (99–213, 32%)	Phylogeny. Synteny with other teleosts. Domain structure (see text).
Medaka	None	20(+): 13051104–13070105	No	Not applicable	No	8226≫	No—5′ and 3′ coding sequence incomplete	2742	PKD domains (294–350, 380–440); REJ domain (482–1081); GPS motif (1541–1574); PLAT/LH2 domain (1650–1768); Polycystin-cation-channel domain (2270–2530)	Pkd1l1 (aa 2–65, 52%)	Phylogeny. Synteny and expression data with other teleosts. Domain structure (see text).
Stickleback	None	GroupXXI(+): 5717457–5729908	No	Tblastn with the full-length zebrafish Pkd1l1 protein sequence identified this non-annotated region of homology on the forward strand of GroupXXI. Unfortunately, there is no mRNA or reference sequence available to determine the protein structure for this region. However, the genes flanking this locus show synteny with those flanking the other teleost *pkd1l1* genes described in this table (see Figure 4C).						Pkd1l1 (aa 3–67, 48%); Pkd2 (aa 132–211, 33%); Pkd2l1 (aa 124–212, 28%)	Synteny with other teleost *pkd1l1* genes.
***pkd1l2a***											
Zebrafish	*pkd1l3 (1 of 4);* *Pkd1l3 (3 of 4)*	7(-): 64953104–65012621	ENSDARG00000105344; ZFIN ID: ZDB-GENE-050208-89	No—5′ coding sequence incomplete	Yes	8526	Yes∧	2485	Lectin C-type domain (51–159); Galactose binding lectin domain (174–258); REJ domain (648–919); GPS motif (1298–1335); PLAT/LH2 domain (1408–1512); Polycystin-cation-channel domain (2019–2437)	Pkd1 (aa 217–296, 24%); Pkd1l1 (aa 130–209, 30%); Pkd1l2b (aa 26–419, 60%); Pkd2 (aa 35–80, 98–171, and 138–247, 38%); Pkd2l1 (aa 35–78 and 97–250, 33%)	Phylogeny, synteny, domain structure, and expression data (see text).
Green spotted pufferfish	*pkd1l3 (2 of 2)*	5(–): 6132261–6135595	ENSTNIG00000009353 (Novel)	No—5′ and 3′ coding sequence incomplete	No	2109	No	703	Polycystin-cation-channel domain (250–657)	Pkd1l1 (aa 4–109, 38%); Pkd1l2a (aa 1–426, 61%); Pkd1l2b (aa 1–417, 57%); Pkd2 (aa 48–255, 34%); Pkd2l1 (aa 49–226, 30%)	Phylogeny. Synteny with other teleost *pkd1l2a* genes.
***pkd1l2a***											
Medaka	*pkd1l3 (1 of 2)*	3(+): 18867151–18882761	ENSORLG00000007572 (Novel)	No—5′ and 3′ coding sequence incomplete	No	7410	No	2470	Lectin C-type domain (47–157); Galactose binding lectin domain (172–255); REJ domain (600–872); GPS motif (1276–1314); PLAT/LH2 domain (1387–1500); Polycystin-cation-channel domain (1992–2412)	Pkd1l2a (aa 32–426, 56%); Pkd1l2b (aa 1–417, 37%); Pkd2 (aa 48–262, 27%); Pkd2l1 (aa 49–235, 35%)	Phylogeny. Synteny with other teleost *pkd1l2a* genes. Domain structure.
Stickleback	*pkd1l3 (2 of 2)*	GroupII(+): 10511520–10528238	ENSGACG00000015742 (Novel)	No—3′ coding sequence incomplete	No	7373	No	2458	Lectin C-type domain (49–158); Galactose binding lectin domain (173–256); REJ domain (644–895); GPS motif (1285–1323); PLAT/LH2 domain (1396–1499); Polycystin-cation-channel domain (1999–2416)	Pkd1l2a (aa 30–426, 68%); Pkd1l2b (aa 25–417, 56%); Pkd2 (aa 48–182, 45%); Pkd2l1 (aa 49–183, 40%)	Phylogeny. Synteny with other teleost *pkd1l2a* genes. Domain structure.
***pkd1l2b***											
Zebrafish	*pkdrej (1 of 2); pkdrej (2 of 2); pkd1l3*	7(+): 67029232–67087646	ENSDARG00000101214; ZFIN ID: ZDB-GENE-141222-87	Incomplete—incorrect exon boundaries (see text)	Yes	7898	Yes∧	1902	Lectin C-type domain (42–150); Galactose binding lectin domain (164–245); GPS motif (760–797); PLAT/LH2 domain (871–895); Polycystin-cation-channel domain (1441–1857)	Pkd1 (aa 152–197, 37%); Pkd1l2a (aa 15–75 and 139–417, 57%); Pkd2 (aa 36–172 and 178–245, 39%); Pkd2l1 (aa 36–80 and 98–246, 33%)	Phylogeny and domain structure (see text).
Green spotted pufferfish	*pkd1l3 (1 of 2)*	Un_random (+): 65025810–65043223	ENSTNIG00000003968 (Novel)	No—5′ and 3′ coding sequence incomplete	No	7143	No	2381	Lectin C-type domain (10–117); GPS motif (1249–1287); Polycystin-cation-channel domain (1927–2352)	Pkd1l1 (aa 4–87, 23%); Pkd1l2a (aa 32–426, 55%); Pkd1l2b (aa 27–384, 43%); Pkd2 (aa 48–221, 34%); Pkd2l1 (aa 49–226, 31%)	Phylogeny.
***pkd1l2b***											
Medaka	*pkd1l3 (2 of 2)*	21(–): 29489012–29547025	ENSORLG00000018124 (Novel)	No—5′ and 3′ coding sequence incomplete	No	7293	No	2431	Lectin C-type domain (44–157); Galactose binding lectin domain (173–255); GPS motif (1278–1316); PLAT/LH2 domain (1389–1491); Polycystin-cation-channel domain (1976–2389)	Pkd1l2a (aa 32–417, 59%); Pkd1l2b (aa 1–224 and 277–383, 61%); Pkd2 (aa 137–182, 54%); Pkd2l1 (aa 49–183, 44%)	Phylogeny and domain structure. Synteny with Stickleback *pkd1l2b* gene.
Stickleback	*pkd1l3 (1 of 2)*	GroupXVI(–): 4027610–4044964	ENSGACG00000002153 (Novel)	No—5′ and 3′ coding sequence incomplete	No	4965	No	1655	GPS motif (500–538); PLAT/LH2 domain (611–713); Polycystin-cation-channel domain (1218–1626)	Pkd1l2a (aa 30–426, 59%); Pkd1l2b (aa 1–390, 56%); Pkd2 (aa 48–182, 26%); Pkd2l1 (aa 49–183, 26%)	Phylogeny. Synteny with Medaka *pkd1l2b* gene.
***pkd1l3***											
Zebrafish	*pkd1l3*	7(–): 56186991–56215290^**^	ENSDARG00000091803 (Zv9)^**^; ZDB-GENE-060810-132	Yes	No	3252	Yes	1083	Lectin C-type domain (55–155); GPS motif (493–531); PLAT/LH2 domain (604–713)	**NO HITS**	Synteny with amniote *PKD1L3* genes.
Green spotted pufferfish	None	Un_random (–): 80610479–80612299	ENSTNIG00000005751 (Novel)	Yes	No	1194	Yes	397	GPS motif (34–68); PLAT/LH2 domain (144–258)	**NO HITS**	Synteny with amniote *PKD1L3* genes.
Medaka	None	3(+): 16335410–16337664^^***^^	No	Tblastn with the full-length longest isoform of mouse PKD1L3 protein sequence identified this non-annotated region of homology on the forward strand of Chromosome 3. Unfortunately, there is no mRNA or reference sequence available to determine the protein structure for this region. However, the genes flanking this locus show synteny with those flanking the other teleost putative *pkd1l3* orthologs described in this table (see Figure 4E).						**NO HITS**	Synteny with amniote *PKD1L3* genes.
Stickleback	None	GroupII(+): 8624083–8628300	ENSGACG00000015469 (Novel)	No—5′ and 3′ coding sequence incomplete	No	1575	No	525	GPS motif (183–219); PLAT/LH2 domain (293–409)	**NO HITS**	Synteny with amniote *PKD1L3* genes.
***pkd2***											
Zebrafish	None	1(+): 49895013–49913204	ENSDARG00000014098; ZFIN ID: ZDB-GENE-040827-4	Yes	No	3336	Yes	904	Polycystin-cation-channel domain (204–624)	Pkd1 (aa 112–195, 45%); Pkd1l1 (aa 132–215, 38%); Pkd1l2a (aa 48–276, 36%); Pkd1l2b (aa 48–346, 38%); Pkd2l1 (aa 14–393, 56%)	Phylogeny, synteny and domain structure.
***pkd2***											
Green spotted pufferfish	None	18(+): 7404161–7408783	ENSTNIG00000011045	No—5′ and 3′ coding sequence incomplete	No	2553	No	851	Polycystin-cation-channel domain (170–589)	Pkd1 (aa 83–348, 22%); Pkd1l1 (aa 4–109, 32%); Pkd1l2a (aa 41–86 and 100–368, 31%); Pkd1l2b (aa 36–81 and 99–362, 33%); Pkd2 (aa 15–394, 76%); Pkd2l1 (aa 15–395, 52%)	Phylogeny, synteny and domain structure.
Medaka	None	1(–): 20624482–20633236	ENSORLG00000007003	Yes	No	2709	Yes	902	Polycystin-cation-channel domain (211–629)	Pkd1 (aa 15–154, 34%); Pkd1l1 (aa 23–108, 33%); Pkd1l2a (aa 41–177, 33%); Pkd1l2b (aa 36–187, 34%); Pkd2 (aa 15–399, 59%); Pkd2l1 (aa 4–242 and 263–395, 48%)	Phylogeny, synteny and domain structure.
Stickleback	None	GroupIX(–): 7437117–7444710	ENSGACG00000017335	No—5′ coding sequence incomplete	No	3386	No	903	Polycystin-cation-channel domain (214–633)	Pkd1 (aa 85–154, 43%); Pkd1l1 (aa 29–103, 34%); Pkd1l2a (aa 104–177, 42%); Pkd1l2b (aa 99–172, 43%); Pkd2 (aa 15–395, 77%); Pkd2l1 (aa 15–242 and 262–395, 56%)	Phylogeny, synteny and domain structure.
***pkd2l1***											
Zebrafish	None	13(+): 25324869–25340926	ENSDARG00000022503; ZFIN ID: ZDB-GENE-030616-558	Yes	No	2718	Yes	790	Polycystin-cation-channel domain (139–559)	Pkd1 (aa 113–150, 42%); Pkd1l1 (aa 140–212, 30%); Pkd1l2a (aa 49–226, 34%); Pkd1l2b (aa 49–136 and 140–185, 40%); Pkd2 (aa 15–242 and 262–395, 55%)	Phylogeny, domain structure and expression data (see text). Synteny with other teleost *pkd2l1* genes.
Green spotted pufferfish	None	17(–): 1034193–1037975	ENSTNIG00000013092	No—5′ and 3′ coding sequence incomplete	No	1977	No	659	Polycystin-cation-channel domain (69–489)	Pkd1 (aa 82–333, 23%); Pkd1l1 (aa 4–107, 23%); Pkd1l2a (aa 104–255, 28%); Pkd1l2b (aa 98–246, 30%); Pkd2 (aa 14–393, 58%); Pkd2l1 (aa 15–395, 65%)	Phylogeny and domain structure. Synteny with other teleost *pkd2l1* genes.
***pkd2l1***											
Medaka	None	15(+): 29369729–29374015	ENSORLG00000013731	No—5′ and 3′ coding sequence incomplete	No	2058	No	686	Polycystin-cation-channel domain (149–569)	Pkd1 (aa 83–154, 37%); Pkd1l2a (aa 17–259, 31%); Pkd1l2b (aa 98–172, 35%); Pkd2 (aa 14–393, 59%); Pkd2l1 (aa 15–394, 73%)	Phylogeny and domain structure. Synteny with other teleost *pkd2l1* genes.
Stickleback	None	GroupVI(–): 2954929–2959614	ENSGACG00000003378	No—5′ and 3′ coding sequence incomplete	No	2250	No	750	Polycystin-cation-channel domain (153–573)	Pkd1 (aa 83–154, 41%); Pkd1l1 (aa 28–121, 29%); Pkd1l2a (aa 101–255, 36%); Pkd1l2b (aa 36–80 and 96–246, 33%); Pkd2 (aa 1–393, 42%); Pkd2l1 (aa 1–242 and 258–394, 58%)	Phylogeny and domain structure. Synteny with other teleost *pkd2l1* genes.

*Column 2 lists former gene names used in previous genome assemblies. Column 3 shows position in current version of appropriate genome. Where an Ensembl gene annotation exists that supports a particular pkd gene, this is listed in column 4, and column 5 describes whether this annotation is accurate and/or complete. For zebrafish genes, the ZFIN ID is also given in column 4. Zebrafish transcripts that we have mapped in this study are indicated in column 6. ˆ Mapped sequences have been submitted to NCBI (see Section Materials and Methods). Columns 7 and 9 indicate transcript and protein lengths, in base pairs and amino acids, respectively. Column 8 indicates whether a complete transcript for the gene is available, either on Ensembl or as a result of our analyses in this paper. Column 10 indicates protein domains identified by searching against Pfam protein database. Column 11 indicates results (percentage amino acid sequence identity) from Tblastn searches of respective genome with polycystin-cation-channel domains of each zebrafish Pkd protein. In most cases, a specific gene was only found by a subset of these Tblastn searches. The amino acid regions of the zebrafish query sequence that align with the candidate sequence are indicated. Column 12 lists data that support the annotations shown here (see Section Results for more info). Alternative transcripts are available in Ensembl (see Section Materials and Methods for genome assemblies) for the following species and genes: zebrafish—pkd1b and pkd1l1, green spotted pufferfish—pkd1, pkd1b, pkd1l1 and pkd1l2a, medaka—pkd2l1 and stickleback—pkd1 and pkd2. We cannot rule out the possibility that alternative transcripts may exist for other genes. Where multiple transcripts exist, the longest protein isoform is shown*.

**Figure 3:**
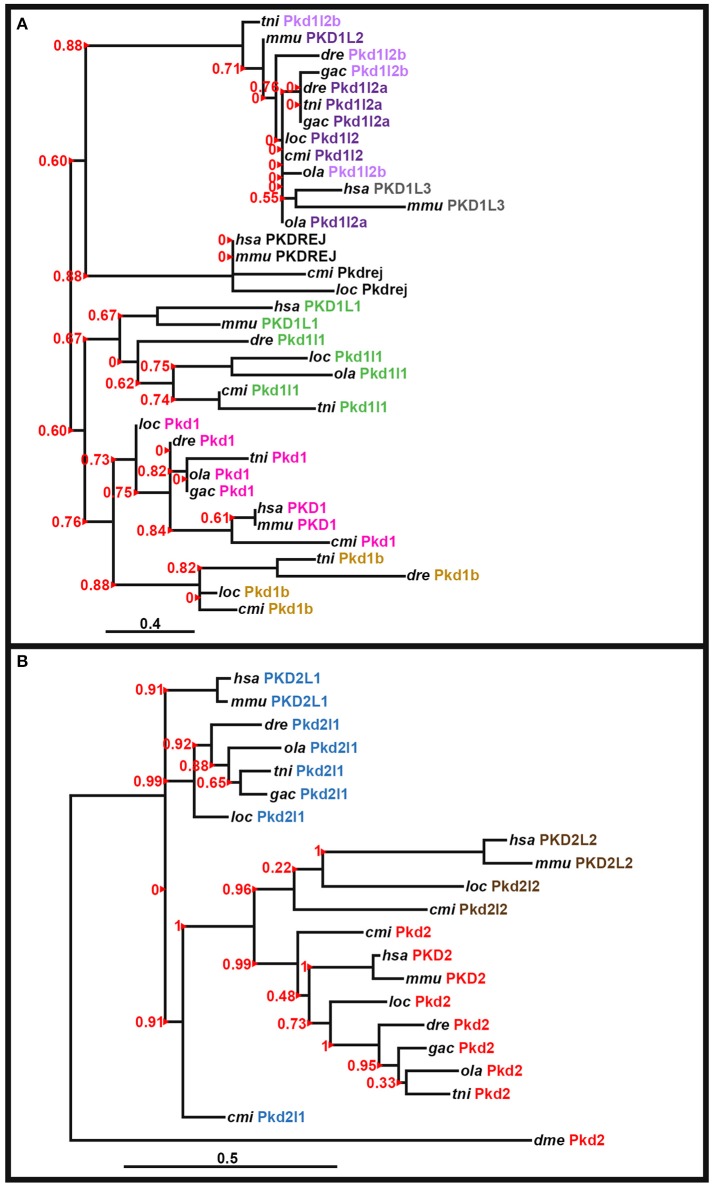
**Phylogenetic analysis of PKD proteins**. Phylogenetic analysis of human (*Homo sapiens, hsa*), mouse (*Mus musculus, mmu*), spotted gar (*Lepisosteus oculatus, loc*), elephant shark (*Callorhinchus milii, cmi*), zebrafish (*Danio rerio, dre*), medaka (*Oryzias latipes, ola)*, green spotted pufferfish (*Tetraodon nigroviridis, tni)*, and stickleback *(Gasterosteus aculeatus, gac)* PKD1-like proteins **(A)** and PKD2-like proteins with the *Drosophila melanogaster* (*dme*) Pkd2 protein as an outgroup **(B)**. In both cases a region of the polycystin-cation-channel domain that was present in all of the proteins was used (see Section Materials and Methods and Supplementary Figures 1, 2). Both analyses used a maximum likelihood method, with WAG substitution, performed using PhyML (v3.1/3.0 Alrt; see Section Materials and Methods). Human PKD1L2, stickleback *Pkd1b* and teleost, and spotted gar Pkd1l3 proteins are not included as they lack the polycystin-cation-channel domain. We did not include an invertebrate protein in the analysis of PKD1-like proteins as the evolution of this gene family seems to be more complex and while vertebrate PKD1-like proteins do have some homology to invertebrate proteins, this homology is limited and we did not identify an invertebrate protein that had good support for being a clear outgroup for this family. aLRT Sh-like branch support values are shown in red to the left of each branch. Red arrowheads indicate the branch that each value corresponds to. Scale bar = 0.4 nucleotide substitutions per site **(A)**, 0.5 nucleotide substitutions per site **(B)**.

**Figure 4:**
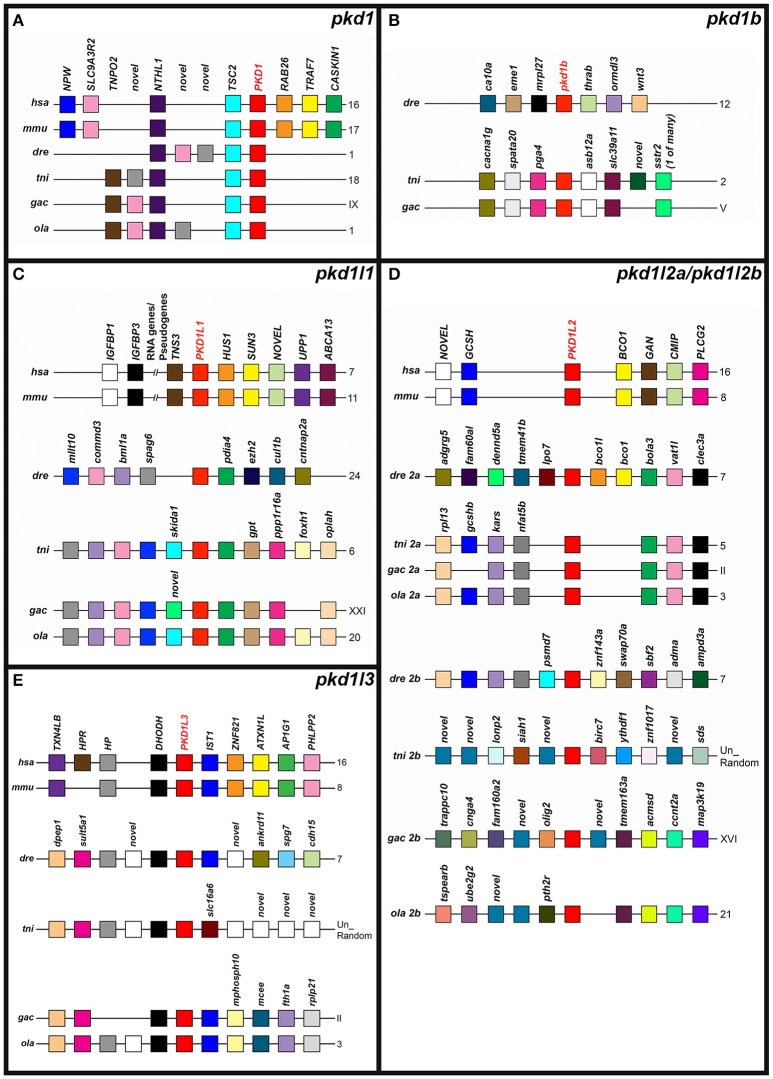
**Conserved synteny around zebrafish ***pkd1-like*** genes**. Examination of syntenic relationships between *pkd* and neighboring genes in genomic regions associated with zebrafish *pkd1* family genes. Species is indicated on left and chromosomes on right. Un-Random (*tni*), unordered random sequences that have yet to be assigned to a chromosome. *hsa*, human (*Homo sapiens*); *mmu*, mouse (*Mus musculus*); *dre*, zebrafish (*Danio rerio*); *ola*, medaka (*Oryzias latipes)*; *tni*, green spotted pufferfish (*Tetraodon nigroviridis*); and *gac*, stickleback (*Gasterosteus aculeatus*). *Pkd* genes are indicated in bold red text. Schematics are not to scale. For ease of comparison, gene clusters are shown in the same orientation, even though in some cases, gene organization is as shown, but on the opposite strand of the chromosome. Schematics only include annotated coding genes. Antisense processed transcripts and ribosomal and long-non-coding RNA loci are not included. Colors indicate homologous genes within an individual panel. So, for example, pink genes in *pkd1* **(A)** are homologous to each other (they are all *SLC9A3R2* despite their slightly different positions) but they are not homologous to pink genes in the *pkd1l1* panel. However, gray (novel) genes in **(A)** are an exception, as these three genes are not homologous to each other. We did not find a *pkd1b* gene in medaka, and none of the genes flanking *pkd1b* in green spotted pufferfish and stickleback are found near the zebrafish *pkd1b* gene **(B)**. The *PKD1L1* locus is syntenic within but not between amniotes and teleosts **(C)**. Zebrafish *pkd1l2a* is the only teleost gene to share synteny with both the aminote and other teleost *PKD1L2* loci **(D)**. Only stickleback and medaka *pkd1l2b* genes share any synteny among the *pkd1l2b* genes **(D)**. As in amniotes, all teleost putative partial *pkd1l3* orthologs are flanked by *dhodh* genes **(E)**.

* Indicates that there are two potential medaka pkd1 sequences. The currently annotated pkd1 gene [ENSORLG00000019755, formerly pkd1 (2 of 2)] is present on Scaffold1073 of the medaka genome. This is the only gene on Scaffold1073 and it encodes a 3400 amino acid protein containing all of the protein domains found within other Pkd1 proteins, with the exception of the carboxy-terminus polycystin-cation-channel domain. Tblastn analysis against the medaka genome with the polycystin-cation-channel domains of each of the zebrafish Pkd proteins did not detect a polycystin-cation-channel domain on Scaffold1073, but it did find one in novel gene ENSORLG00000011636 [formerly pkd1 (1 of 2)] on chromosome 1. This novel gene shares synteny and phylogeny with both mammalian and teleost pkd1 genes (Figures 3, 4A). Tblastn with Scaffold1073 Pkd1 protein sequence failed to detect any more Pkd1-like protein sequences encoded upstream of this novel gene on chromosome 1. Therefore, there is no evidence at present that these two sequences constitute different parts of the same gene, although the protein domains that they encode suggest that this may be the case.

¥ *When Tblastn was performed against the stickleback genome with full-length zebrafish Pkd1b, sequence with homology to REJ and PLAT/LH2 domains was detected in novel gene ENSGACG00000005441. This gene was called pkd1b in a previous genome assembly and it has conserved synteny with green spotted pufferfish pkd1b. However, unlike all bona-fide Pkd proteins, this stickleback protein does not contain a carboxy-terminus polycystin-cation-channel domain. ≫When Tblastn was performed against the medaka genome with the polycystin-cation-channel domain from zebrafish Pkd1l1, a region of homology on chromosome 20 was identified that shared synteny with pkd1l1 genes in other teleosts. Since this genomic region was not annotated, we searched the literature for putative medaka pkd1l1 sequences and identified a 8226 bp partial mRNA sequence lacking both start and stop codons, reported by Kamura et al. (2011)(Genbank reference # AB573426.1). This sequence overlaps the region of homology identified in our Tblastn analyses and the protein that it encodes is consistent with Pkd1l1 in other species. In addition, this genomic region shares synteny with other pkd1l1 genomic regions and a previous report (Kamura et al., 2011) demonstrated that this gene is co-expressed with pkd2 in Kupffer's vesicle, consistent with our expression data for zebrafish pkd1l1 (Figures 6D–F). All of these data suggest that this non-annotated region encodes medaka pkd1l1. We show here the protein domains for the BAJ65629.1 protein record associated with the AB573426.1 mRNA sequence. The pkd1l1 gene in stickleback is found in a non-annotated region of GroupXXI, for which there is no supporting transcript data in either Ensembl or NCBI. However, Tblastn with zebrafish Pkd1l1 produced 23 hits within this region of Group XXI, covering the full-length of the zebrafish Pkd1l1 protein. Furthermore, this region has conserved synteny with pkd1l1 regions in other teleosts (Figure 4C)*.

** This sequence is a non-annotated region that falls within the final intron (intron 6–7) of the third transcript of the sult5a1 gene (ENSDART00000162934.1) in the current GRCz10 Ensembl assembly. This corresponds to the locus occupied by gene ENSDARG00000091803 in Zv9. We are showing information for the retired Zv9 annotation, since our synteny analyses support this sequence being a partial pkd1l3 ortholog.

*** *This sequence is a non-annotated region that falls between the genes sult5a1 and hp on chromosome 3 in the current assembly of the medaka genome. Sequence homology and synteny analyses suggest that this is a putative medaka partial pkd1l3 ortholog*.

Therefore, we used inverse PCR and overlapping PCR amplicons, to identify/map the correct *pkd1* sequence (see Section Materials and Methods, Figure 1A and Table 1). All but 173 bases of the older ENSDART00000039911 transcript (Zv9) are present in our newly mapped *pkd1* transcript as are all but the first 45 nucleotides of the current ENSDART00000039911 transcript (GRCz10). However, compared to our new transcript, there are some gaps in the Zv9 transcript, and we have also identified novel 5′ sequence that is not present in either transcript nor the 5′ genomic sequence in GRCz10, suggesting that there may be sequence missing from chromosome 1 in Ensembl. Taken together, our data suggest that a transcript of at least 18401 bp exists that encodes a 4608 amino acid protein and we have deposited this sequence in NCBI (accession number KY074550). This sequence lacks a start methionine. There is a methionine codon in-frame at 527–529 nucleotides (Figure 1A), but the region upstream of this methionine encodes a leucine-rich-repeat domain which is conserved in mouse, human and stickleback PKD1. Therefore, we think that this methionine is unlikely to be the start codon. We consider that the start codon is more likely to be a putative in-frame methionine 54 nucleotides upstream of our present transcript. We are confident that this gene is *pkd1*, given our synteny and phylogeny analyses discussed below (see also Table 1).

#### pkd1b

In contrast to *pkd1*, since the inception of this study the annotation of ENSDARG00000033029, the gene called *pkd1b* in our initial bioinformatic searches, has remained unchanged within Ensembl. The current longest *pkd1b* transcript, ENSDART00000153412.2, encodes a 3817 amino acid protein (Table 1, Figure 2). This Ensembl sequence is strongly supported by the reference sequence XM_017358624.1, which has the Gene ID 565697 (https://www.ncbi.nlm.nih.gov/gene/), with the exception that this reference sequence encodes an additional 73 amino acids at the amino terminus. Therefore, we cannot rule out the possibility that the *pkd1b* transcript might be longer than that currently shown in Ensembl. However, this would not affect the predicted domain structure of Pkd1b protein, as the additional 73 amino acids do not contain any additional predicted protein domains (Figure 2). We are confident that this gene is *pkd1b* based on the protein domains that it encodes (Figure 2) and our phylogeny analyses discussed below (see also Table 1). However, it is worth noting that, despite its name and the absence of this gene in mammals, we do not think that this gene is a teleost duplicate of *pkd1* (see Section Discussion below).

#### pkd1l1

The reference sequence XM_009297371, called *pkd1l1* in our initial analyses, aligns with the forward strand of chromosome 24. In Ensembl Zv9 this region contained a small ORF of 225 amino acids, called *pkd1l3 (2 of 4)*. In GRCz10, this region of homology is now called *BX005392.1* (gene ENSDARG00000099162). The longest transcript at this locus is ENSDART00000169516.1, which encodes a protein of 2153 amino acids. Whilst there is no stop codon in either ENSDART00000169516.1 or an additional shorter transcript associated with *BX005392.1*, there is a putative stop codon in-frame 27 bp downstream in the 3′ flanking sequence of ENSDART00000169516.1. To assess whether this locus might encode a *pkd* gene, we generated two alternative riboprobes, one designed against exons 39–43 and the other against exons 46–49. As described below, both of these riboprobes labeled putative taste receptors and we also saw expression in dorsal forerunner cells / Kupffer's vesicle similar to *pkd1l1* expression in medaka (Kamura et al., 2011). The structure of this protein, including its shorter polycystin-cation-channel domain and fewer PKD domains is consistent with that of PKD1L1 in other vertebrates (Figure 2). In addition, our synteny and phylogenetic analyses described below also suggest that this gene is *pkd1l1*.

#### pkd1l2a

The XM_002662913 (used to be called *pkd1l3*) reference sequence aligns with the reverse strand of chromosome 7. In an early release of Zv9, this region contained two adjacent novel genes, ENSDARG00000074116 and ENSDARG00000090210, encoding proteins of 488 and 294 amino acids respectively (Figure 1B). Temporarily these genes received the annotations *pkd1l3 (1 of 4)* and *pkd1l3 (3 of 4)*, before being retired from the final release of Zv9. Given the proximity of these genes to one another on the same chromosome and their consecutive alignment with the XM_002662913 reference sequence, we hypothesized that these genes might constitute different parts of one longer gene. Consistent with this, riboprobes generated against each gene produced identical expression patterns in zebrafish ventral spinal cord and putative taste receptors (Figure 1B; and see expression analyses below).

In GRCz10 this locus is now called *pkd1l2a*. A single 17-exon transcript, ENSDART00000173234.1, is predicted to encode an 1124 amino acid protein. However, the 5′ sequence of this transcript only partially overlaps the 3′ sequence of ENSDARG00000090210 and is, therefore, likely to be incomplete (Figure 1B). It is also likely to contain inaccuracies because sequence present in our ENSDARG00000074116 riboprobe is annotated as being intronic in ENSDART00000173234.1.

To identify the correct gene sequence, we used the XM_002662913 sequence, which aligns with both of the retired *pkd1l3* genes as well as sequence upstream of them, to map the mRNA transcript using RT-PCR. Since a preliminary protein domain search of the reference sequence suggested we might be missing amino-terminus sequence, we used the RefSeq GFF3 annotation import track in GRCz10 to identify a putative locus, *LOC101884812*, immediately upstream (Figure 1B). A protein domain search of this sequence using the Pfam protein database identified lectin and galactose-binding-lectin domains typically found in the amino-terminus of PKD1L2 proteins (Figure 2). We confirmed that these regions were transcribed using RT-PCR (Figure 1B). We also performed inverse PCR to identify any 5′ coding sequence that might be further upstream (Figure 1B). Using these approaches we identified an 8526 bp mRNA transcript, comprising 41 exons, both 5′ and 3′ UTR sequences and encoding a protein of 2485 amino acids that is identical in domain structure and length to mouse PKD1L2 (Figure 2, Table 1). This transcript encompasses both *LOC101884812* and XM_002662913 and contains additional exons not present in either of these sequences (Figure 1B). There is also considerable overlap between our transcript and the current ENSDART00000173234.1 transcript (Figure 1B). The structure of the protein encoded by our mapped transcript, including its lack of a PKD domain and the presence of a galactose-binding-lectin domain, is consistent with PKD1L2 proteins in other animals (Figure 2). Our synteny and phylogenetic analyses (see below) also suggest that this is a *pkd1l2* gene. Our *pkd1l2a* transcript has been deposited at NCBI (accession number KY074551).

#### pkd1l2b

In Zv9 the XM_009303604 reference sequence partially aligned with *pkd1l2* (ENSDARG00000088121) on the forward strand of chromosome 7 (1–2985 bp) and to a non-coding region on the forward strand of Zv9_Scaffold3511 (1782–6344 bp), suggesting that the *pkd1l2* annotation on chromosome 7 might be incomplete (Figure 1C). Consistent with this, the 5′ coding sequence of the longest ENSDARG00000088121 transcript, ENSDART00000156286, lacked a start codon and only encoded a protein of 859 amino acids. This gene was subsequently renamed *pkdrej (2 of 2)* in a later Zv9 release. Interestingly, in that same genome release an additional gene, *pkdrej (1 of 2)*, encoding a protein of 124 amino acids, was reported immediately downstream of *pkdrej (2 of 2)*. Our PFAM protein domain analysis revealed that these “Pkdrej” proteins contained classic features of Pkd proteins (Lectin C-type domain, galactose-binding-lectin domain, and GPS motif [Pkdrej (2 of 2)] and PLAT/LH2 domain [Pkdrej (1 of 2)]. However, neither protein contained the REJ domain, present in amniote PKDREJ proteins (Figure 2), nor the polycystin-cation-channel domain present in all other Pkd proteins. By the first release of GRCz10, *pkdrej (2 of 2)* had become *si:ch211-168k15.4* and parts of *pkdrej (1 of 2)* had been included in the largest transcript of a new gene, ENSDARG00000101214, called *pkd1l3*, although in the most recent release of GRCz10 (version 86.10), ENSDARG00000101214 is named *pkd1l2*. These two genes overlap each other (Figure 1C). Exons 10–16 and the first 231 bases of exon 17 of *si:ch211-168k1.5.4* are identical to exons 2–9 of ENSDARG00000101214. However, the *si:ch211-168k15.4* transcript utilizes a stop codon present in intron 9–10 of ENSDARG00000101214, and the transcript for ENSDARG00000101214 (ENSDART00000124969.2) utilizes a start codon present in exon 10 of *si:ch211-168k15.4*. Since the protein encoded by ENSDART00000124969.2 is 1442 amino acids long and contains a polycystin-cation-channel domain, we tested whether this transcript and *si:ch211-168k15.4* might actually be part of a larger *pkd* gene using RT-PCR. Since the 5′ coding sequence is incomplete in *si:ch211-168k15.4* we also performed inverse PCR to identify any 5′ coding sequence that might be further upstream (Table 1). These analyses identified a longer, combined 7898 bp transcript that contains both the *si:ch211-168k15.4* and ENSDARG00000101214 sequences, utilizing a start codon 4 bases upstream of exon 1 in the current *si:ch211-168k15.4* annotation, and transitioning between exon 16 of *si:ch211-168k15.4* immediately into exon 9 of ENSDART00000124969.2. Whilst the identification of the start codon is unique to this study and we have also identified novel 3′ UTR sequence, nucleotides 683–7026 of our new 7898 bp mRNA transcript align perfectly with the original XM_009303604 6344 bp reference sequence. The resulting 1902 amino acid protein has very similar domains to Pkd1l2a (Table 1, Figure 2) and its polycystin-cation-channel domain is most similar to that of Pkd1l2a, with which it has >60% identity, more than 30% higher than with any other zebrafish Pkd protein (Table 2). The lack of a PKD domain and the presence of a galactose-binding-lectin domain are also consistent with PKD1L2 proteins in other animals, with the exception that, unlike zebrafish Pkd1l2a and amniote PKD1L2, this protein is missing a REJ domain. Our synteny and phylogenetic analyses, discussed below, also suggest that this is a *pkd1l2* gene. Therefore, we are confident that this gene is *pkd1l2b* and we have deposited the transcript sequence at NCBI (accession number KY074552).

**Table 2:** **Similarities of polycystin-cation-channel domains of zebrafish Pkd proteins**.

**Pkd Protein**	**Polycystin-cation-channel domain size (amino acids)**	**Pkd protein**						
		**Pkd1**	**Pkd1b**	**Pkd1l1**	**Pkd1l2a**	**Pkd1l2b**	**Pkd2**	**Pkd2l1**
Pkd1	392	100	27.78	16.95	23.81	25.20	25.86	24.07
Pkd1b	293	27.78	100	21.64	20.98	20.49	20.63	19.23
Pkd1l1	181	16.95	21.64	100	28.49	21.79	27.37	22.91
Pkd1l2a	419	23.81	20.98	28.49	100	62.26	29.56	29.80
Pkd1l2b	417	25.20	20.49	21.79	62.26	100	28.47	27.97
Pkd2	421	25.86	20.63	27.37	29.56	28.47	100	57.38
Pkd2l1	421	24.07	19.23	22.91	29.80	27.97	57.38	100

*Percentage identity between polycystin-cation-channel domains of zebrafish Pkd proteins generated using Clustal Omega (see Section Materials and Methods). Column 2 indicates the size of the polycystin-cation-channel domain in amino acids. Compare the protein in each row to the protein in each column to read the pairwise identity percentage*.

#### pkd2

During this study the annotation of the gene called *pkd2* remained unchanged within Ensembl. The *pkd2* mRNA reference sequence identified at the start of this study, DQ175629.1, aligns with the 14 exons present in the current Ensembl *pkd2* transcript, ENSDART00000020412.7, although the latter contains additional UTR sequence. This suggests that *pkd2* (ENSDARG00000014098) encodes a 904 amino acid protein. Our synteny and phylogeny analyses (see below) confirm that this gene is *pkd2*. Similar to PKD2 proteins in other vertebrates, the main conserved domain in the encoded protein is the polycystin-cation-channel domain (Figure 2, Table 1).

#### pkd2l1

The annotation of the gene called *pkd2l1* also remained unchanged within Ensembl during this study. The *pkd2l1* mRNA reference sequence XM_690312 aligns with 100% homology to exons 1–9, 11–12, and 14–15 of the current *pkd2l1* transcript ENSDART00000145948.1, which contains the additional exons 10 and 13. This suggests that *pkd2l1* generates a 790 amino acid protein and is encoded by ENSDARG00000022503. Consistent with this, our *in situ* hybridization riboprobe (see expression analyses below), which was designed against the 3′ coding and UTR sequence present in ENSDART00000145948.1 (nucleotides 2062–2614), generated data similar to expression reported using a riboprobe designed against more upstream sequence (nucleotides 1148–2022; Djenoune et al., 2014). As for zebrafish Pkd2, and PKD2 family proteins in other vertebrates, the main conserved domain in this protein is the polycystin-cation-channel domain (Figure 2, Table 1). Our synteny and phylogeny analyses (see below) also suggest that this gene is *pkd2l1*.

We also performed additional bioinformatics searches using a newer version of the zebrafish genome released during our study, GRCz10, to test if there were any additional potential *pkd* genes. For this, we identified peptide sequences for the polycystin-cation-channel domain in each of the zebrafish *pkd* genes and performed Tblastn analyses with each of these sequences against this newer version of the genome (see Section Materials and Methods). We used the polycystin-cation-channel domain because this is the domain that defines Pkd proteins (Figure 2). When we did this, several of the domains identified other already-identified *pkd* genes (Table 1), but no new *pkd* genes were identified.

Since the zebrafish *pkd* genes that we had identified included only one set of potential teleost-duplicates or ohnologs, (just *pkd1l2a*/*pkd1l2b* as we do not think that *pkd1 and pkd1b* are teleost duplicates because these genes exist in both cartilaginous and holostei fish as described below), we further investigated whether teleost ohnologs of any additional *pkd* genes might exist by searching for *pkd* genes in medaka, stickleback and green spotted pufferfish. We performed a textual search for *pkd* genes, and also blasted the polycystin-cation-channel domain for all seven zebrafish Pkd proteins, against each of these genomes. We identified the same complement of seven genes in both stickleback and green spotted pufferfish and six genes in medaka (*pkd1b* was missing; Table 1). For each zebrafish Pkd protein, the polycystin-cation-channel domain had greatest homology with the gene that our phylogeny and synteny analyses suggest is its closest ortholog in each of the other teleost genomes (Table 1). Whilst Pkd1l2b in green spotted pufferfish and stickleback have slightly higher sequence homology with the polycystin-cation-channel domain of zebrafish Pkd1l2a than Pkd1l2b, Pkd1l2a in both of these teleosts shows even higher sequence homology with zebrafish Pkd1l2a. Therefore, these data, together with our phylogeny and synteny analyses, suggest that we have correctly classified the genes that encode these proteins (Table 1). We found no evidence in any of the teleosts examined for additional duplicate (ohnolog) *pkd* genes.

Interestingly, our Tblastn analyses with full-length zebrafish Pkd1b identified a small region of homology in each of the teleost genomes with the PLAT/LH2 region of Pkd1b (data not shown). Visual inspection of each of these loci revealed conserved synteny with the region surrounding amniote *PKD1L3* genes (**Figure 4E**). To assess whether these teleost genomes might contain *pkd1l3* orthologs, we performed Tblastn analyses with full-length mouse PKD1L3 (Supplementary Table 4). These identified the same loci. The putative *pkd1l3* locus is not annotated in either zebrafish or medaka genomes, although a transcript is present in a previous zebrafish genome assembly (ENSDARG00000091803 in Zv9, Table 1). In the green spotted pufferfish and stickleback genomes, this locus contains a novel gene. Consistent with our Tblastn analyses with polycystin-cation-channel sequences, we have not detected these sequences encoding this domain within any of these loci. Given that the polycystin-cation-channel domain is the one domain that is present in all PKD proteins, we do not consider these sequences bona-fide *pkd* genes and therefore have not analyzed them further in this study.

Given that we found sequences with homology to part of the *pkd1l3* gene, to confirm that *pkd212* and *pkdrej* are absent in teleosts we performed Tblastn with full-length mouse PKD2L2 and PKDREJ against all of the teleost genomes discussed above. Both of these analyses only produced alignments with already identified Pkd proteins. Therefore, we are confident that there are no *pkd212* or *pkdrej* genes in these teleost genomes.

To investigate potential relationships between the zebrafish *pkd* genes we aligned the polycystin-cation-channel domains for each of the Pkd proteins and determined the percentage identity of this domain between each of them. Pkd1l2a and Pkd1l2b have the highest identity (62%; Table 2), which is consistent with them being recently duplicated genes. Pkd2l1 and Pkd2 also have a high degree of identity at 57%. However, all of the other pair-wise comparisons have <30% identity at the amino acid level.

To investigate the evolution of zebrafish *pkd* genes we identified all of the PKD genes in both spotted gar (*Lepisosteus oculatus*; a holostei fish) and elephant shark (*Callorhinchus milii*; a cartilaginous fish). In both of these species we found 8 *pkd* genes: *pkd1, pkd1b, pkd1l1, pkd1l2, pkdrej, pkd2, pkd2l1*, and *pkd2l2* (Figure 3, Supplementary Table 5). Unlike mammals, spotted gar, and elephant shark both have a *pkd1b* gene. In contrast, similar to mammals and unlike teleosts they only have one *pkd1l2* gene (Supplementary Table 5). However, like teleosts, spotted gar only has a partial *pkd1l3* sequence that lacks the polycystin-cation-channel domain but is located in a region with conserved synteny with other *pkd1l3* regions (data not shown). It is currently less clear whether a *pkd1l3* gene exists in elephant shark. In the mammalian, teleost and holostei genomes that we have examined, *PKD1L3* is always located close to a gene called *DHODH* (Figure 4E). *dhodh* is located on Scaffold_12 of the elephant shark current genome, but we did not find any evidence for a *pkd* gene nearby (data not shown), although we cannot rule out the possibility that this gene exists elsewhere in the genome.

To confirm orthologous relationships between teleost and mammalian *PKD1-family* and *PKD2-family* genes we performed phylogenetic analyses of human (*Homo sapiens*), mouse (*Mus musculus*), spotted gar (*Lepisosteus oculatus*), elephant shark (*Callorhinchus milii*), zebrafish (*Danio rerio*), medaka (*Oryzias latipes)*, green spotted pufferfish (*Tetraodon nigroviridis)*, and stickleback *(Gasterosteus aculeatus)* PKD1-like and PKD2-like proteins. We used the region of the polycystin-cation-channel domain that was present in all of the proteins and both Neighbor-Joining (NJ) and maximum likelihood methods (see Section Materials and Methods, Supplementary Figures 1–2, Figure 3). In the resulting phylogenetic trees (Figure 3; data not shown), all of the zebrafish proteins cluster with the expected proteins from other species, suggesting that their annotations are correct. Consistent with Pkd1l2a and Pkd1l2b being teleost duplicates of PKD1L2 proteins in other vertebrates, all of the PKD1L2 proteins cluster together. Interestingly, in the maximum likelihood tree, mammalian PKD1L3 genes are also contained in this cluster, although this was not the case in the NJ analysis (Figure 3 and data not shown). However, we are confident that none of the teleost *pkd1l2* genes are *pkd1l3* genes as we have also found partial *pkd1l3* genes in teleosts as discussed above.

To further test whether we had correctly identified orthologous relationships, we also examined the genomic regions around each of the zebrafish *pkd* genes and their proposed orthologs in other vertebrates for conserved syntenic relationships with other neighboring genes. We found that the *pkd1* genomic locus contains both a *NTHL1* and *TSC2* gene in humans, mouse and all four teleost species (Figure 4A). In contrast, whilst the genomic regions around green spotted pufferfish and stickleback *pkd1b* share synteny with each other, none of the genes in this region are found near zebrafish *pkd1b* (Figure 4B). The *PKD1L1* locus has considerable shared synteny between human and mouse and between teleosts, but the teleost loci don't have any obvious shared synteny with the amniote loci (Figure 4C). In contrast, *PKD1L2* is located near a *GCSH* and a *BCO1* gene in both mammals and at least one teleost and there are other genes found in common near most of the teleost *pkd1l2a* genes (Figure 4D).

**Figure 5:**
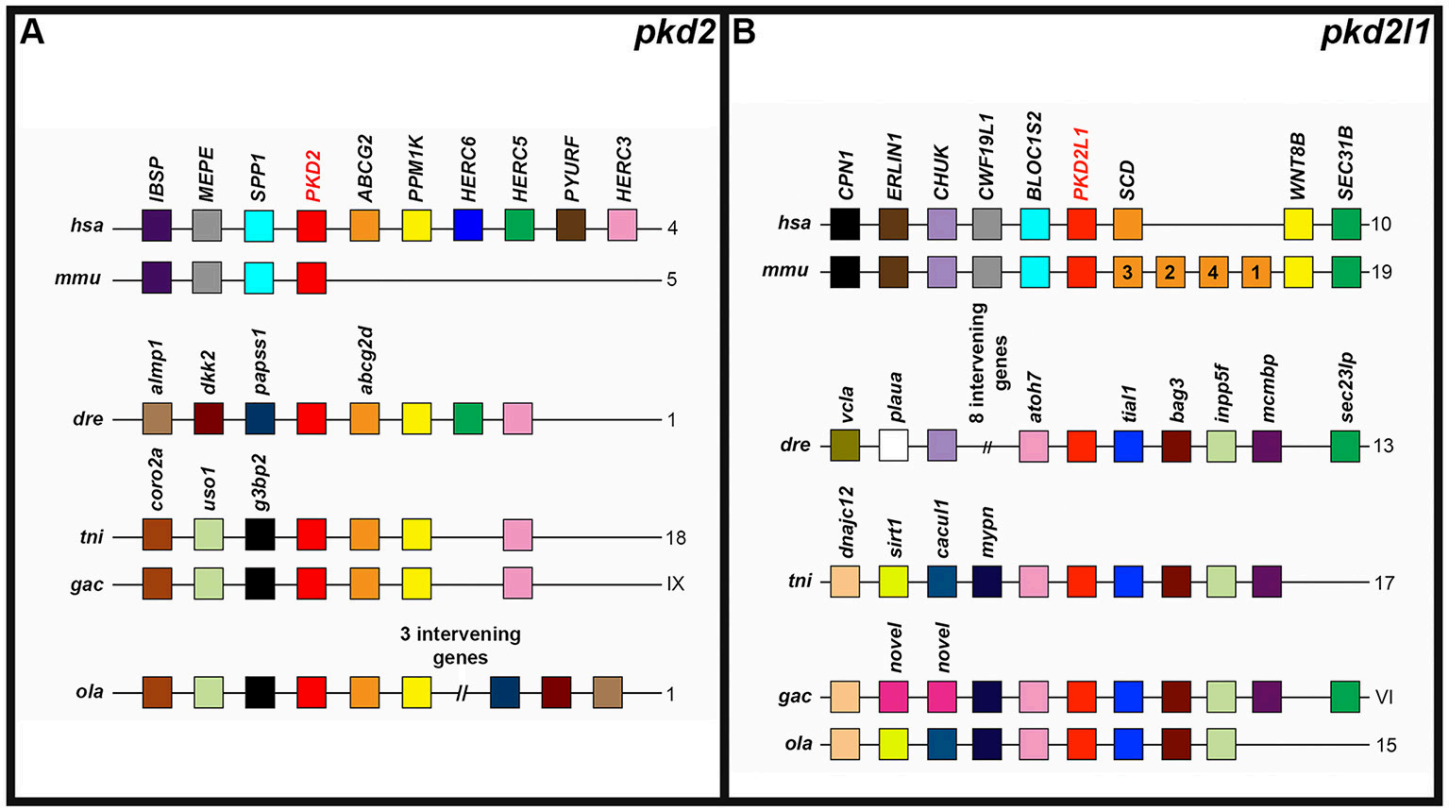
**Conserved synteny around zebrafish ***pkd2-like*** genes**. Examination of syntenic relationships between *pkd* and neighboring genes in genomic regions associated with zebrafish *pkd2* family genes. Species is indicated on left and chromosomes on right. *hsa*, human (*Homo sapiens*); *mmu*, mouse (*Mus musculus*); *dre*, zebrafish (*Danio rerio*); *ola*, medaka (*Oryzias latipes)*; *tni*, green spotted pufferfish (*Tetraodon nigroviridis*); and *gac*, stickleback (*Gasterosteus aculeatus*). *Pkd* genes are indicated in bold red text. Schematics are not to scale. For ease of comparison, gene clusters are shown in the same orientation, even though in some cases, gene organization is as shown, but on the opposite strand of the chromosome. Schematics only include annotated coding genes. Antisense processed transcripts and ribosomal and long-non-coding RNA loci are not included. Colors only indicate homologous genes within an individual panel. So, for example, pink genes in the *pkd2* panel are not homologous to pink genes in the *pkd2l1* panel. The teleost *pkd2* genes share synteny with human but not mouse *PKD2* **(A)**. The teleosts share considerable synteny at the *pkd2l1* locus, but only zebrafish and stickleback *pkd2l1* genes share any synteny with amniotes **(B)**.

The *PKD2* locus, like the *PKD1* locus, also has some conserved synteny between different vertebrates (Figure 5A). This genomic region contains an *ABCG2* and *PPM1K* gene in humans, zebrafish, green spotted pufferfish, medaka, and stickleback. While the mouse *Pkd2* locus doesn't seem to contain these genes—it shares three other genes with the human locus. The *Pkd2l1* locus also has considerable conserved synteny between different teleost genomes and between mouse and human. In addition, a couple of genes are present in both mammals and at least one teleost: *CHUK* (present in mammals and zebrafish) and a *SEC* gene—*SEC31B* (present in mammals) and *sec23lp* (present in zebrafish and stickleback; Figure 5B).

Based on all of these analyses, we are convinced that the genes that we have identified are indeed *pkd1, pkd1b, pkd1l1, pkd1l2a, pkd1l2b, pkd2*, and *pkd2l1* and that these are the only bona-fide *pkd* genes in zebrafish and the other teleosts that we have examined.

### Expression of *pkd* genes during zebrafish development

As described in the introduction, *pkd* genes have important developmental functions in many different tissues and in at least some of these locations, PKD1-like and PKD2-like proteins act in a heteromeric complex. However, before we started this study the expression patterns, and hence the potential functions and binding partners, were not known for several of the zebrafish *pkd* genes. Therefore, we performed a comprehensive expression analysis for all seven genes at developmental stages from 8 h to 5 dpf. We identified expression of specific subsets of these genes in many different tissues/structures as described below.

#### Dorsal forerunner cell and kupffer's vesicle expression

**Figure 6:**
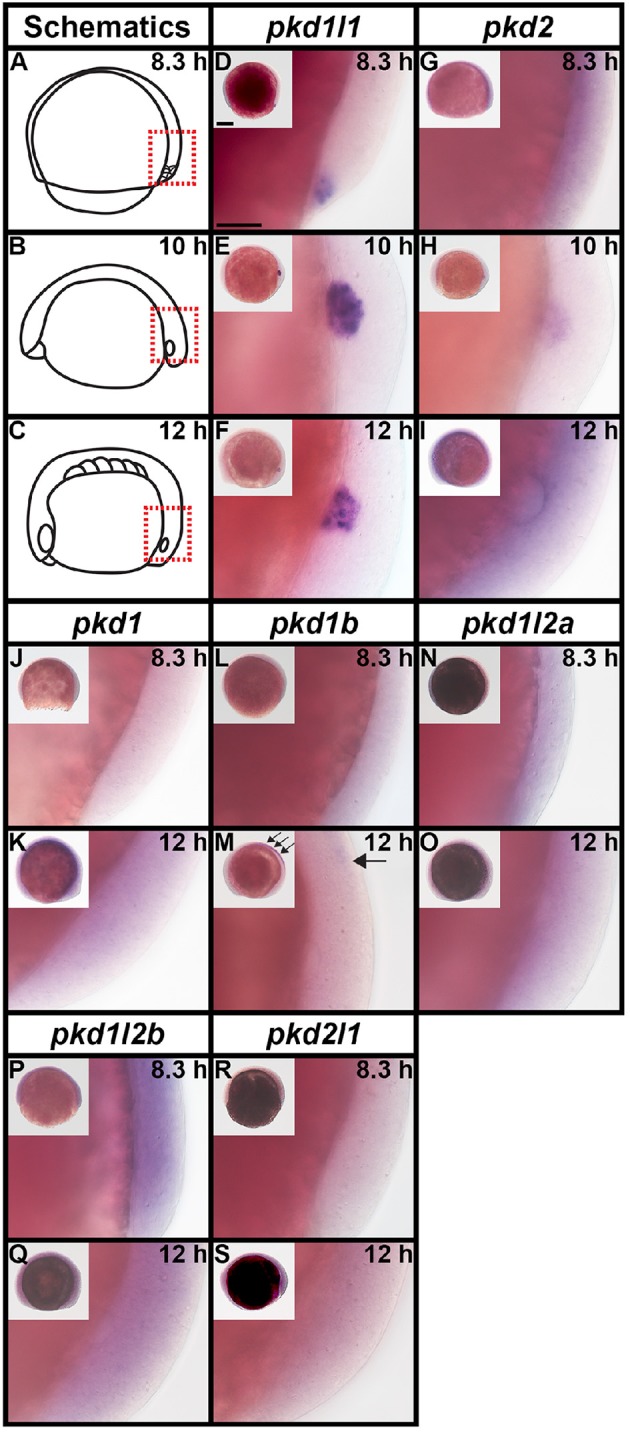
***pkd2*** **and ***pkd1l1*** are expressed in Kupffer's vesicle**. Lateral expression of *pkd* genes at 8.3, 10, and 12 h. Region shown in main panel at each stage is indicated by red dotted boxes in schematics **(A–C)**. Inset images in **(D–S)** show whole-mount view of embryo, dorsal forerunner cells/KV located in bottom right-hand corner. There is no expression of *pkd1, pkd1b, pkd1l2a, pkd1l2b*, or *pkd2l1* in dorsal forerunner cells or KV at any of these stages. The boundary of the KV cavity is faintly visible in M as a slightly different focal plane has been shown to include spinal cord expression. However, *pkd1b* is not expressed in the margin of KV. Arrows in **(M)** indicate caudal limit of spinal cord expression of *pkd1b*. *pkd1l1* is expressed in the KV region at all three stages **(D–F)** and *pkd2* is expressed at 10 and 12 h but not 8.3 h **(G–I)**. Scale bar **(D)** = 50 μm, **(D–S)** main panels and 200 μm, inset panels.

As previous studies have established that *pkd2* is expressed in Kupffer's vesicle (KV) during early zebrafish embryogenesis (Bisgrove et al., 2005; Schottenfeld et al., 2007; Roxo-Rosa et al., 2015), we investigated expression of all seven zebrafish *pkd* genes in dorsal forerunner cells/KV at 8.3, 10, and 12 h (Figure 6). We found no expression of *pkd1, pkd1b, pkd1l2a, pkd1l2b*, and *pkd2l1* at any of these stages (Figures 6J–S), with the exception that there is some spinal cord expression of *pkd1b* at 12 h (arrows in Figure 6M). In contrast, *pkd1l1* was expressed in a cluster of dorsal forerunner cells at 8.3 h (Figure 6D), that later condense to form the KV at 10 and 12 h (Figures 6E–F). *pkd2* expression was not detected at 8.3 h (Figure 6G), but was present in a group of cells in the KV region at 10 h, resolving in to a ring of cells surrounding the KV by 12 h (Figures 6H–I).

#### Pronephros/kidney expression

**Figure 7:**
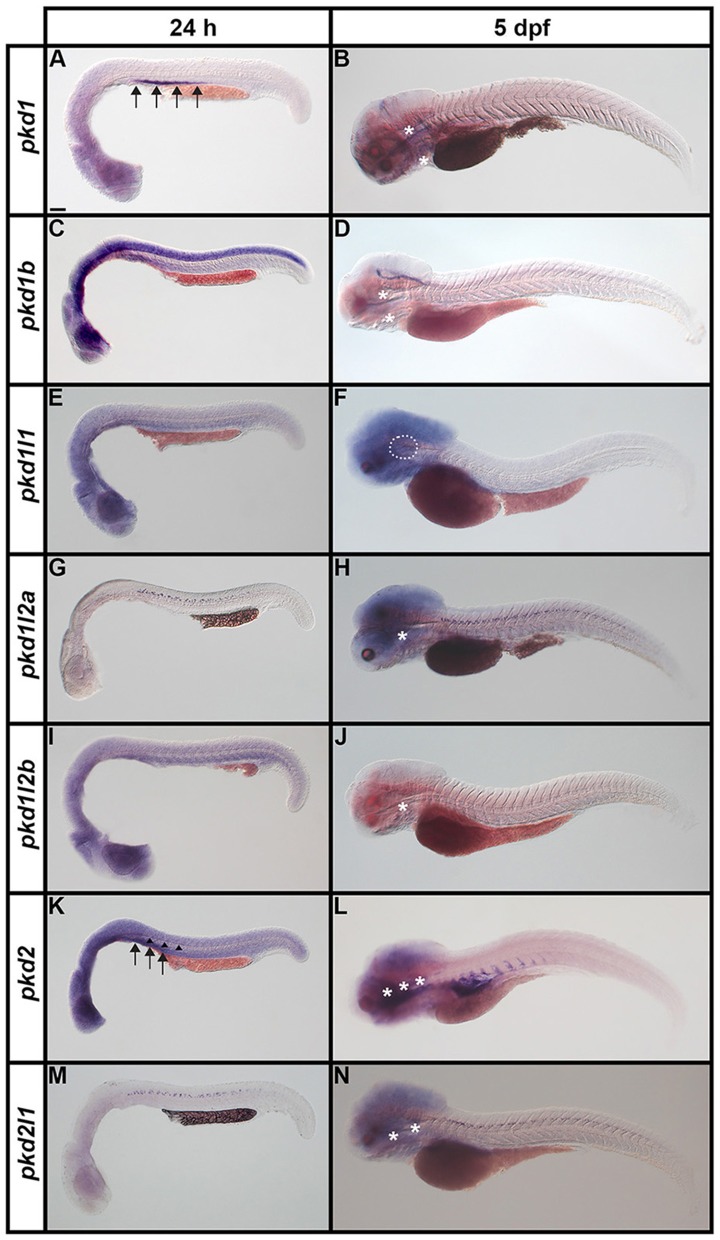
**Expression of ***pkd*** genes in zebrafish embryos and larvae**. Lateral views of whole embryo expression of *pkd* genes at 24 h and 5 dpf. Rostral left, dorsal up. **(A,B)** *pkd1* is strongly expressed in the pronephros at 24 h (arrows, **A**) but not at 5 dpf. By 5 dpf, *pkd1* expression persists only in the putative taste receptors (white asterisks, **B**). **(C,D)** *pkd1b* is broadly expressed throughout the dorsal-ventral hindbrain and spinal cord, and in the caudal-most midbrain at 24 h. By 5 dpf, strong expression persists in the floor plate in the midbrain and hindbrain, whilst weaker expression persists in putative taste receptors of the pharynx (white asterisks, **D**). **(E,F)** *pkd1l1* is not expressed at 24 h but is detected in the ear at 5 dpf (white dotted line, **F**). **(G,H)** *pkd1l2a* is expressed in cells in the ventral-most spinal cord at 24 h. This expression persists at 5 dpf, as does expression in putative taste receptors (white asterisk, **H**). **(I,J)** *pkd1l2b* expression is not detected at 24 h and persists only weakly in the pharyngeal cartilage at 5 dpf (white asterisk, **J**). **(K,L)** *pkd2* is expressed in the pronephros (arrows, **K**) and perhaps very weakly in the floor plate at 24 h (arrowheads, **K**). By 5 dpf, *pkd2* expression is restricted to the ventral region of the rostral somites and putative taste receptors (white asterisks, **L**). **(M,N)** Like *pkd1l2a, pkd2l1* is also expressed in cells in the ventral-most spinal cord at 24 h. This expression also persists at 5 dpf, together with weak expression in putative taste receptors (white asterisks, **N**). Low level diffuse staining in the brain in **(A,C,F,H,L,N)** and more widely in **(E,I,K)** is probably background staining. These embryos were stained for longer periods in order to try and detect any weak, but specific, expression in the spinal cord. As a consequence of this, the brain, which contains large ventricles which sometimes trap RNA riboprobes, often has background staining (see Section Discussion). Scale bar **(A)** = 100 μm.

**Figure 8:**
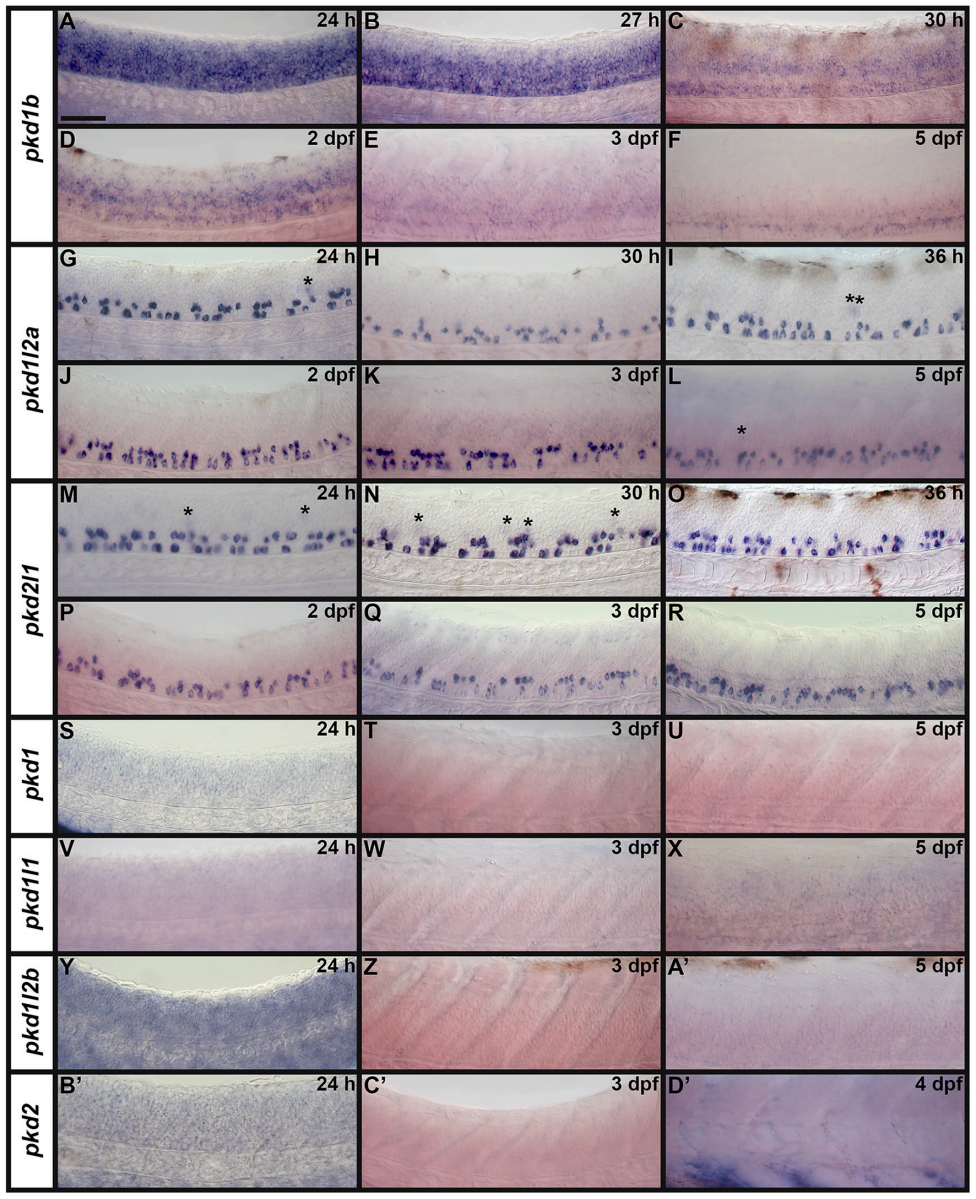
**Spinal cord expression of zebrafish ***pkd*** genes**. Lateral views showing expression of *pkd* genes at 1–5 dpf. Rostral left, dorsal up. **(A–F)** *pkd1b* is expressed broadly in the spinal cord. *pkd1l2a* **(G–L)** and *pkd2l1* **(M–R)** are both expressed in two rows of cells in the ventral spinal cord and occasionally weakly in more dorsal cells (asterisk). **(S–U)** *pkd1*, **(V–X)** *pkd1l1*, **(Y–A')** *pkd1l2b*, and **(B'–D')** *pkd2* are not expressed in spinal cord. Some of these embryos have background expression as we stained them for long periods of time to try and detect any weak, but specific, expression. Expression of *pkd2* is visible in the rostral ventral somites **(D')**. Scale bar **(A)** = 50 μm.

**Figure 9:**
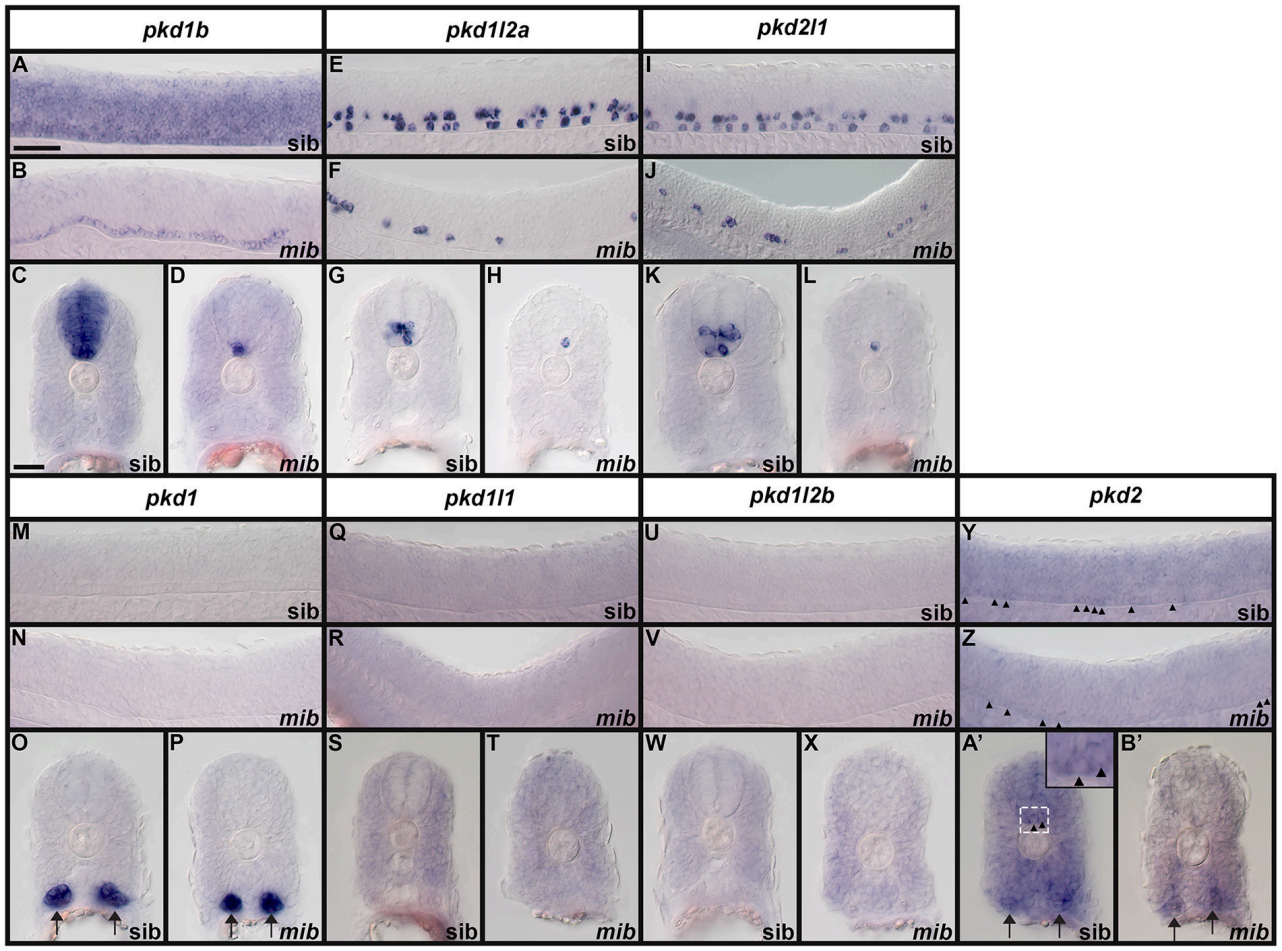
**Expression of zebrafish ***pkd*** genes in ***mindbomb*** mutants**. Lateral views **(A,B,E,F,I,J,M,N,Q,R,U,V,Y,Z)** and cross-sections **(C,D,G,H,K,L,O,P,S,T,W,X,A',B')** of *pkd* expression in the trunk of *mindbomb* mutants and sibling embryos with WT phenotypes. Dorsal is up. In lateral views, rostral is left and only the spinal cord region is shown. Arrows **(O,P,A',B')** indicate pronephros expression. Arrowheads (**Y,Z,A'** and higher magnification inset in **A'**) indicate weak expression of *pkd2* in the floor plate of the spinal cord. The focal plane in **B'** does not include labeled floor plate cells. Scale bar **(A)** = 50 μm (lateral views, **A,B,E,F,I,J,M,N,Q,R,U,V,Y,Z**); Scale Bar **(C)** = 30 μm (cross-sections, **C,D,G,H,K,L,O,P,S,T,W,X,A',B'**) and 10 μm (inset in **A'**).

**Figure 10:**
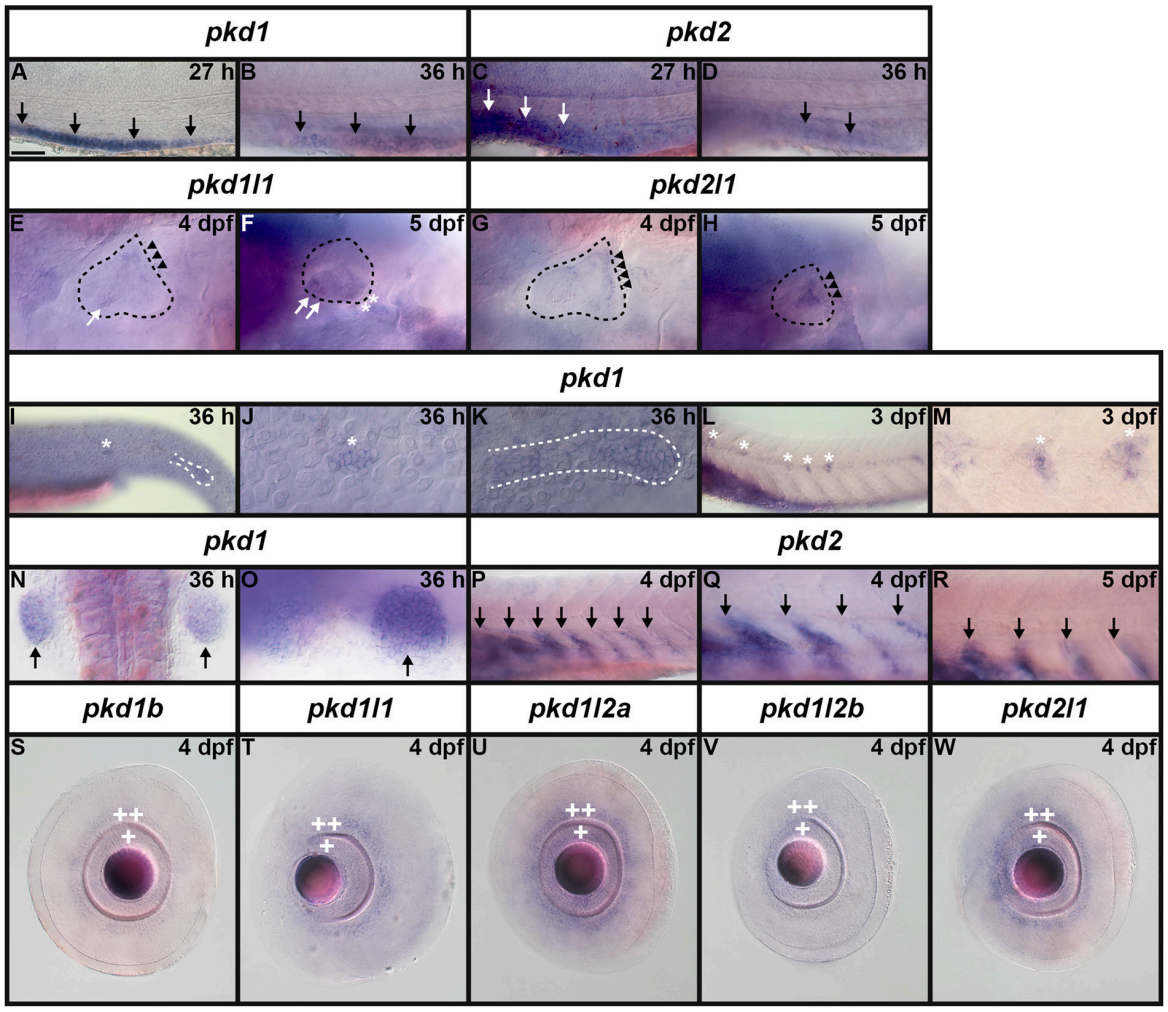
**Expression of ***pkd*** genes in kidney, somites and sensory organs. (A–D)** Lateral view of *pkd1* **(A,B)** or *pkd2* **(C,D)** expression in pronephros (black and white arrows) at 27 and 36 h. Rostral left, dorsal up. *pkd1* is strongly expressed in pronephros at 27 h **(A)**. Expression starts to decline at 36 h **(B)**. Expression of *pkd2* is weak in pronephros at 27 h **(C)** and is reduced even further by 36 h **(D)**. **(E–H)** Lateral expression of *pkd* genes in the ear at 4–5 dpf. Dotted line shows ear boundary. Weak expression of *pkd1l1* **(E)** and *pkd2l1* **(G)** is first detected at 4 dpf in the inner ear ectoderm that supports the posterior canal and posterior crista (black arrowheads). *pkd1l1* is also weakly expressed in the utricular otolith (white arrows). By 5 dpf, *pkd1l1* expression persists in the utricular otolith and the underlying utricular macula (white arrows). It is also expressed in neighboring ectoderm flanking the lateral canal and lateral crista (white asterisks; **F**). At 5 dpf the expression of *pkd2l1* persists in tissue surrounding the posterior canal and posterior crista (black arrowheads; **H)**. **(I–M)** Lateral view of *pkd1* expression in neuromasts (white asterisks) and lateral line primordium (white dotted line) at 36 h and 3 dpf. Rostral left, dorsal up. Weak expression of *pkd1* in neuromasts and lateral line primordium is first detected at 36 h [**I**, higher magnification of the neuromasts **(J)** and lateral line primordium **(K)**]. By 3 dpf expression persists in neuromasts (**L** and higher magnification view, **M**). *pkd1* is also expressed in pectoral fin buds (black arrows) at 36 h [dorsal view, rostral top **(N)**, and lateral view—rostral left, dorsal up **(O)**]. **(P–R)** Lateral expression of *pkd2* in rostral somites at 4 and 5 dpf. Rostral left, dorsal up. *pkd2* is first expressed in the ventral half of each rostral somite at 4 dpf (black arrows in **P**, higher magnification in **Q**) and persists at 5 dpf (black arrows in **R**). **(S–W)** Lateral expression of *pkd* genes in the eye at 4 dpf. Rostral left, dorsal up. *pkd1b, pkd1l1, pkd1l2a, pkd1l2b*, and *pkd2l1* are expressed in the ganglion cell layer (adjacent to lens, single white cross) and amacrine cells (outer cell layer immediately adjacent to ganglion cell layer, double white cross) of the eye at 4 dpf. The expression of *pkd1b* **(S)** and *pkd1l2b* is weak **(V)** and the expression of *pkd1l1* **(T)**, *pkd1l2a* **(U)**, and *pkd2l1* **(W)** is stronger. Only the expression of *pkd1l2b* persists in these cell layers at 5 dpf (data not shown). Scale bar **(A)** = 23 μm **(J,K,M)**; 42 μm **(E–H,O)**; 50 μm **(A–D,Q,R)**; 55 μm **(N)**; 62.5 μm **(S–W)**; and 100 μm **(I,L,P)**.

It has already been reported that *pkd2* expression is enriched in developing zebrafish pronephros (Bisgrove et al., 2005; Schottenfeld et al., 2007), and Pkd2 protein is present in zebrafish kidney epithelial cells (Obara et al., 2006). However, before this study, it was unknown if any other *pkd* genes are expressed in the developing zebrafish pronephros. Therefore, we examined expression in WT embryos (8.3, 10, 12, 24, 27, 30, 36, and 48 h, 3, 4, and 5 dpf). We found no expression of *pkd1b, pkd1l1, pkd1l2a, pkd1l2b*, or *pkd2l1* in the developing pronephros at any of these stages (Figures 6–**9** and data not shown). In contrast, while *pkd1* and *pkd2* are not expressed in presumptive pronephric mesoderm at 8.3, 10, or 12 h (Figures 6G–K), they are both expressed in the pronephros by 24 h, although the expression of *pkd1* is much stronger than that of *pkd2* (Figures 7A,K, **9O,P,A',B'**). The expression of *pkd1* in the pronephros persists until 3 dpf. We also found that weak *pkd2* expression persists in the pronephros during the pharyngula period (arrows in 27 and 36 h) but it was not enriched above the otherwise ubiquitous expression of *pkd2* by 2 dpf (**Figures 10A–D** and data not shown).

#### Spinal cord expression

Zebrafish *pkd2l1* was recently shown to be expressed in a unique population of spinal cord cells, called Kolmer Agduhr (KA) cells or cerebrospinal fluid-contacting neurons (CSF-cNs; Djenoune et al., 2014). In addition, *pkd1b* has been reported as being expressed broadly in the spinal cord at late somitogenesis stages but restricted to medial floor plate ependymal cells by 3.5 dpf (Mangos et al., 2010) and low level expression of *pkd2* has been observed in the floor plate during somitogenesis stages (Bisgrove et al., 2005; Schottenfeld et al., 2007). Therefore, we were very interested in investigating expression of *pkd* genes in spinal cord cells, particularly to see if we could identify a potential partner for Pkd2l1 in KA cells and/or additional Pkd proteins expressed in the floor plate.

We examined spinal cord expression of all 7 *pkd* genes in WT embryos at 12, 24, 27, 30, 36, and 48 h, and 3, 4, and 5 dpf. In addition, we examined expression in *mindbomb* mutants at 24 h. *mindbomb* encodes an E3-ubiquitin-ligase that is required for efficient Notch signaling. In *mindbomb* mutants Notch signaling is lost and, as a result, the vast majority of spinal cord progenitor cells precociously differentiate as early-forming populations of spinal cord neurons at the expense of later forming neurons and glia (Jiang et al., 1996; Schier et al., 1996; Itoh et al., 2003; Park and Appel, 2003; Batista et al., 2008). Therefore, comparing expression of genes in the spinal cords of *mindbomb* mutants and WT embryos enables us to distinguish between progenitor domain expression (which should be lost) and post-mitotic expression (which is often, although not always, expanded). In addition, if a gene is expressed very weakly in post-mitotic spinal cord cells, its expression is usually easier to observe in *mindbomb* mutants, where the expression is often expanded and stronger (Batista et al., 2008). Therefore, examining expression in *mindbomb* mutants also helps us to be more confident about whether a gene is expressed in the spinal cord or not.

**Figure 11:**
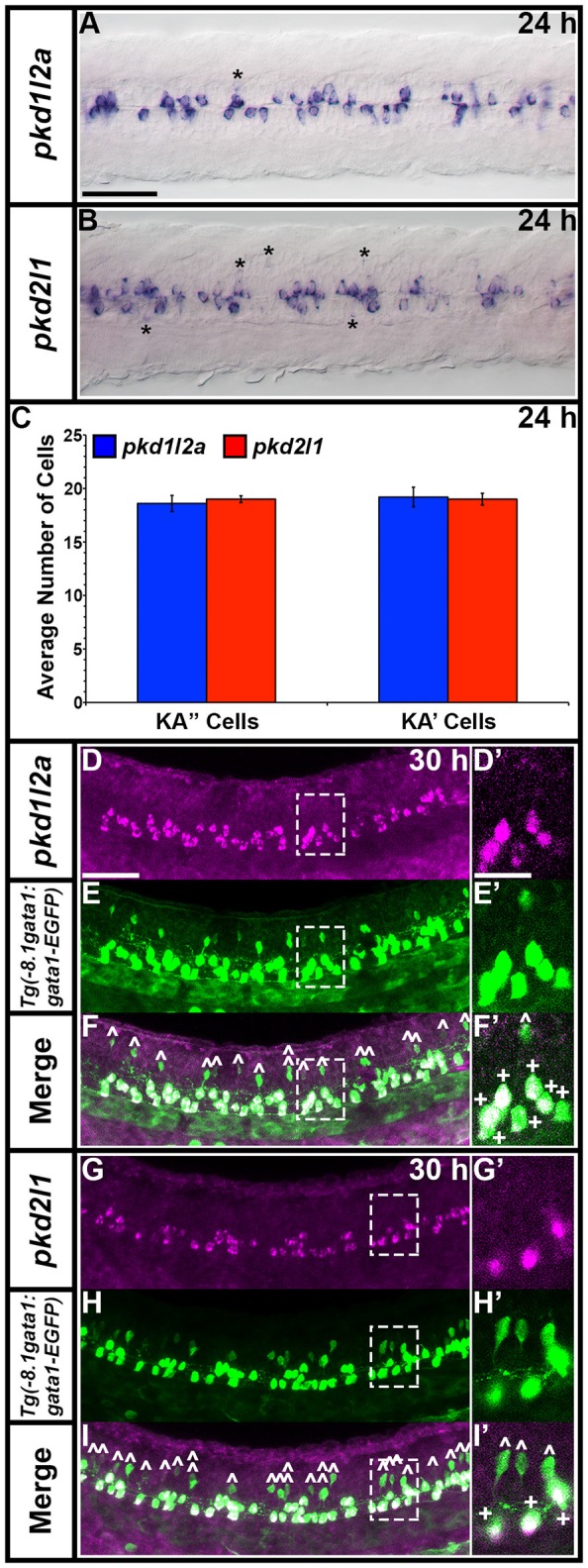
***pkd1l2a*** **and ***pkd2l1*** are co-expressed in zebrafish KA cells**. Dorsal view of *pkd1l2a* **(A)** and *pkd2l1* **(B)** expression in 24 h spinal cord. Rostral left. Most of the labeled cells are KA cells that abut the central canal. Asterisks indicate expression in occasional weak, more lateral cells (that correspond to the more dorsal cells indicated in Figures 8G–R). Prolonged staining sometimes reveals additional weak, lateral cells (data not shown). **(C)** Average number of cells (y-axis) expressing *pkd1l2a* (blue) and *pkd2l1* (red) in KA″ and KA′ cells (x-axis) at 24 h in WT spinal cord region adjacent to somites 6–10 (*n* = 5). Error bars indicate standard error of the mean. There is no statistical difference between the number of *pkd1l2a* and *pkd2l1*-expressing KA″ (*p* = 0.6419) and KA′ (*p* = 0.8571) cells respectively (Student's *t*-test). These data do not include occasional non-KA lateral cells (2 cells each in 2/5 *pkd2l1*-labeled embryos; 0 cells in 5 *pkd1l2a*-labeled embryos). **(D–F',G–I')** Lateral views of zebrafish spinal cord at 30 h. Anterior left, dorsal top. *In situ* hybridization (purple) for *pkd1l2a* **(D,D')** and *pkd2l1* **(G,G')**, EGFP immunohistochemistry (green) in *Tg(–8.1gata1:gata1-EGFP)* embryos **(E,E',H,H')** and merged views **(F,F',I,I')**. **(D'–I')** Magnified single confocal plane of white dotted box region. 100% of *pkd1l2a* and *pkd2l1*-expressing KA cells co-express *Tg(–8.1gata1:gata1-EGFP)* and 100% of GFP-positive *Tg(–8.1gata1:gata1-EGFP)* KA cells co-express either *pkd1l2a* (**F**, indicated with + in **F'**) or *pkd2l1* (**I**, indicated with + in **I'**). No GFP-positive *Tg(–8.1gata1:gata1-EGFP)* dorsal V2b cells co-express either *pkd1l2a* (white ˆ in **F,F')** or *pkd2l1* (white ˆ in **I,I'**). Double-labeled cells are not indicated in **(D–I)** main panels as they are so numerous. Scale bar **(A)** = 50 μm **(A,B)**. Scale bar **(D)** = 50 μm **(D–I)** and 20 μm **(D'–I')**.

Both WT and *mindbomb* mutant expression analyses suggest that *pkd1, pkd1l1*, and *pkd1l2b* are not expressed in zebrafish spinal cord (Figures 7A,B,E,F,I,J, 8S–A', 9M–X and Supplementary Figures 3G–U). It is likely that *pkd2* is also not expressed in spinal cord, other than very weakly in floor plate (Figures 7K,L, 8B'–D', 9Y–B' and Supplementary Figures 3V–Z). In contrast, *pkd1b* was expressed broadly in the spinal cord and *pkd1l2a* and *pkd2l1* were both expressed in post-mitotic cells in ventral spinal cord (Figures 7–9, **11**, see more detailed descriptions below).

**Figure 12:**
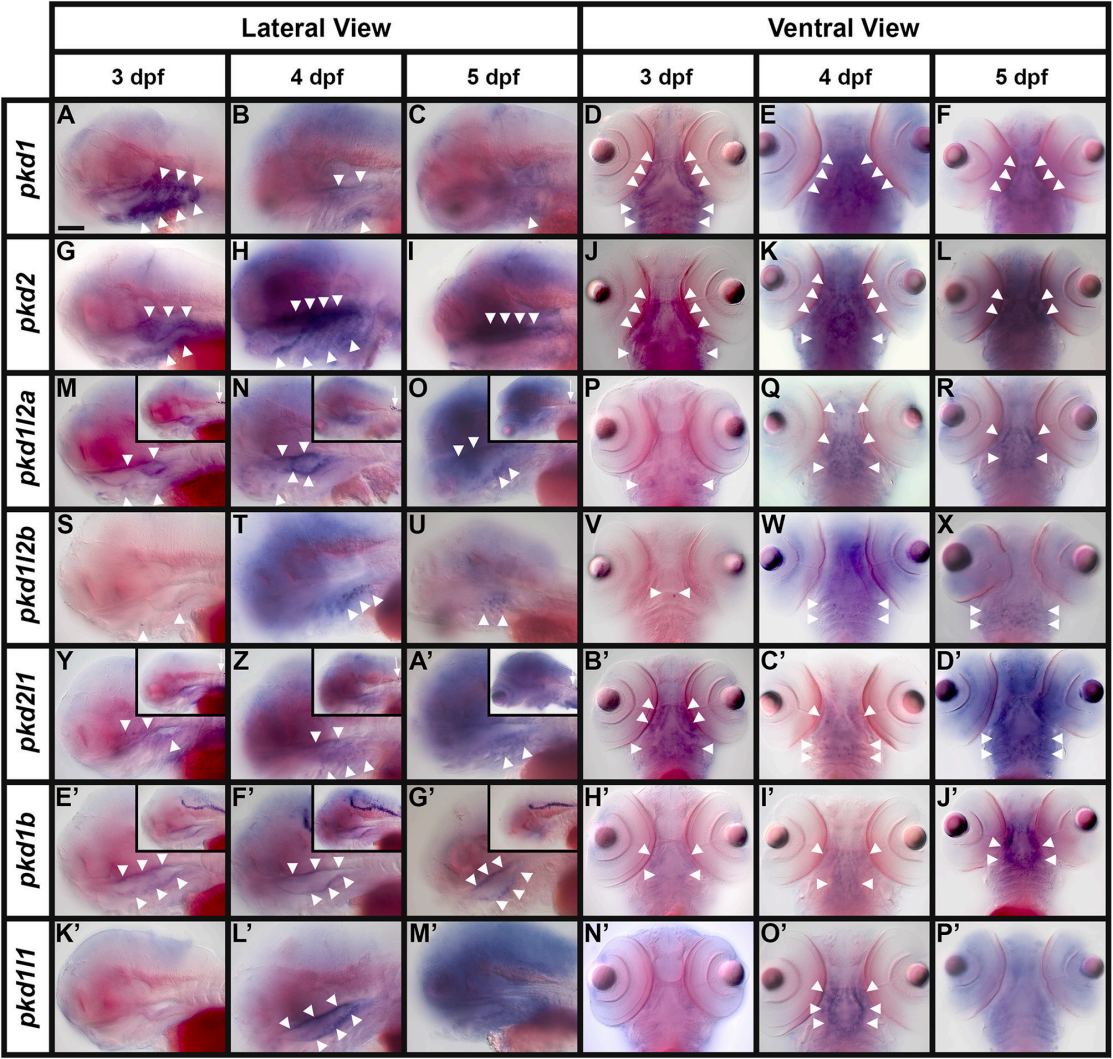
**Expression of ***pkd*** genes in taste bud regions**. Lateral **(A–C,G–I,M–O,S–U,Y,Z,A',E'–G',K'–M')** and ventral **(D–F,J–L,P–R,V–X, B'–D',H'–J',N'–P')** views of *pkd* gene expression at 3, 4, and 5 dpf. Rostral is left Rostral is left **(A–C,G–I,M–O,S–U,Y,Z,A',E'–G',K'–M')** and top **(D–F,J–L,P–R,V–X,B'–D',H'–J',N'–P')**. In most of the lateral views, the eyes are out of focus. Insets in **(M–O)** and **(Y–A')** show expression of *pkd1l2a* and *pkd2l1* in KA cells in the rostral spinal cord (small white arrows). Insets in **(E'–G')** show expression of *pkd1b* in the floor plate of the midbrain and hindbrain. White arrowheads indicate the locations of pharyngeal expression. Scale bar **(A)** = 100 μm.

*pkd1b* is expressed very broadly throughout the dorsal/ventral extent of the spinal cord at 24 h in what appears to be mainly progenitor cells (Figures 8A, 9A,C). By 27 h, expression in the most dorsal part of the spinal cord has reduced. By 30 h the expression has resolved into two broad domains in the ventral spinal cord, one just above the notochord and one in the middle of the dorsal/ventral axis. This continues until 5 dpf, at which point expression in the more dorsal domain is much weaker (Figures 8B–F). Consistent with *pkd1b* being expressed by progenitor cells, most of its spinal expression is lost in *mindbomb* mutants, with the exception of the floor plate expression, which remains. This suggests that *pkd1b* may be expressed in floor plate in addition to being broadly expressed in spinal cord progenitor cells (Figures 9B,D). Consistent with this, we observe *pkd1b* expression in the floor plate of the hindbrain from 24 h until at least 5 dpf (insets in **Figures 12 E'–G'**).

*pkd1l2a* and *pkd2l1* have very similar spinal expression patterns. Both genes are already expressed in two rows of ventral cells by 24 h (Figures 8G,M) as well as being expressed more weakly in occasional more dorsal cells (Figures 8, **11**). This expression also extends into caudal hindbrain (insets in **Figures 12M–O, Y–A'**). Unusually for post-mitotic spinal cord cells, but consistent with KA cells, these cells are located medially in ventral spinal cord, in positions where they can contact the CSF-containing central canal (**Figures 11A,B**, 9E,G,I,K). The expression of both of these genes continues throughout all of the stages that we examined, although the two rows become less distinct at later stages and *pkd2l1* expression seems to become weaker in the more ventral row of cells by 4 dpf (Figures 8H–L,N–R). To confirm that these genes are both expressed by KA cells, we performed double labels with *Tg(*−*8.1gata1:gata1-EGFP)* zebrafish which express GFP in both KA and V2b cells in the spinal cord (Kobayashi et al., 2001; Batista et al., 2008). Consistent with the previous report (Djenoune et al., 2014), we find that *pkd2l1* is expressed in all ventral KA″ cells and more dorsal KA′ cells. In addition, we show for the first time that zebrafish *pkd1l2a* is also expressed in both of these cell types (**Figures 11C–I'**).

#### Ear expression

We also detected expression of two *pkd* genes in specific territories of the ear at 4–5 dpf (Figures 10E–H). We observed *pkd1l1* and *pkd2l1* expression in the ectoderm of the inner ear that supports the posterior canal and posterior crista at 4 dpf. *pkd1l1* is also weakly expressed in the utricular otolith. By 5 dpf, *pkd1l1* expression persists in the utricular otolith and the underlying utricular macula. It is also expressed in the neighboring ectoderm flanking the lateral canal and lateral crista (Figures 10E,F). In contrast, the expression of *pkd2l1* persists in the tissue surrounding the posterior canal and posterior crista (Figures 10G,H).

#### Lateral line and neuromasts

Interestingly, we also detected expression of *pkd1*, and only *pkd1*, in the neuromasts (asterisks, Figures 10I,J,L,M) and lateral line primordium (white dotted line, Figures 10I,K). This expression was first apparent at 36 h and it persists in the neuromasts until 3 dpf, but is lost by 4 dpf.

#### Fin buds

Similarly, out of all 7 *pkd* genes, we only detected expression of *pkd1* in the pectoral fin buds. *pkd1* is expressed in these structures at 36, 48 h, and 3 dpf, but we could not detect expression above background by 4 dpf (arrows in Figures 10N,O and data not shown). At 36 and 48 h, *pkd1* expression is ubiquitous throughout these structures but as reported previously (Mangos et al., 2010) expression is restricted to the base of the fin by 3 dpf.

#### Somite expression

We also only detected expression of one *pkd* gene in somites. *pkd2* is first expressed in the ventral half of each rostral somite at 4 dpf and this expression persists at 5 dpf (Figure 7L, arrows in Figures 10P–R).

#### Eye expression

We detected transient expression of five different *pkd* genes in the retina. *pkd1b, pkd1l1, pkd1l2a, pkd1l2b*, and *pkd2l1* are expressed in the ganglion cell layer (adjacent to the lens, single cross) and the amacrine cells (outer cell layer immediately adjacent to the ganglion cell layer, double cross) of the eye at 4 dpf. The expression of *pkd1b* and *pkd1l2b* is weak, but the expression of *pkd1l1, pkd1l2a*, and *pkd2l1* is stronger (Figures 10S–W). However, only *pkd1l2b* expression persists at 5 dpf (data not shown).

#### Taste bud expression

In other vertebrates, PKD1L3 and PKD2L1 have been proposed to function as sour taste receptors (Huang et al., 2006; Ishimaru et al., 2006, although also see Nelson et al., 2010; Hofherr and Kottgen and references therein). In zebrafish, the taste buds first form at about 3 dpf on the lips/jaw and pharyngeal arches and then at 4 dpf they also form on the mouth and oro-pharyngeal cavity (Hansen et al., 2002; Kapsimali et al., 2011). Consistent with possible expression in the taste buds, we observe dynamic expression of all seven zebrafish *pkd* genes in the pharyngeal and/or jaw regions at these stages.

**Figure 13:**
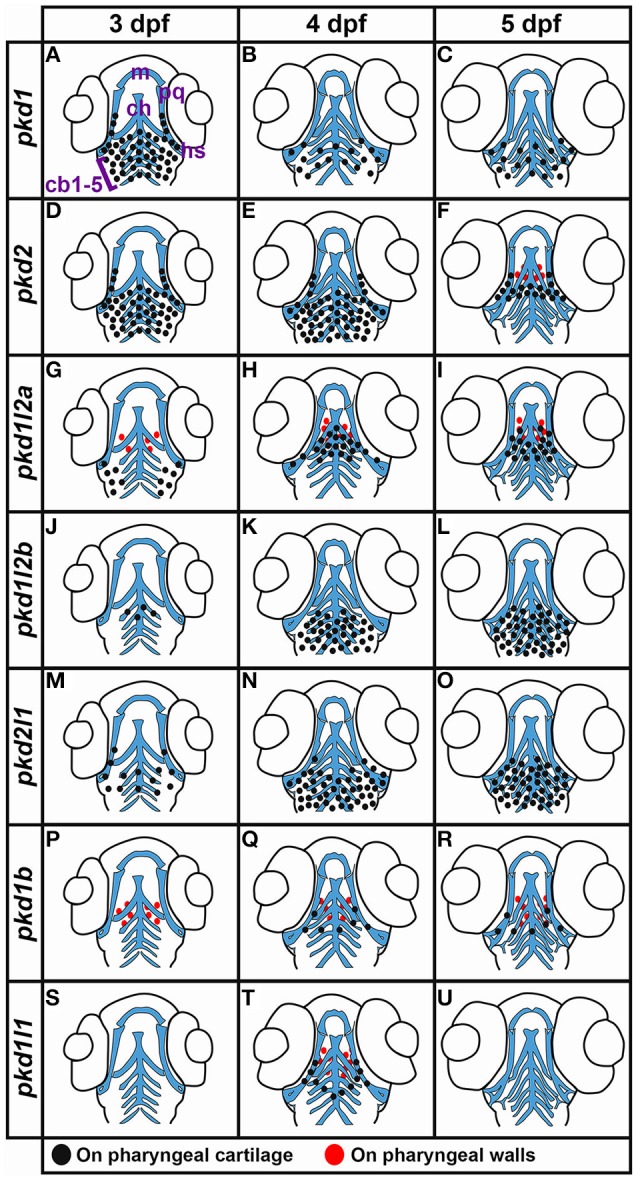
**Summary of ***pkd*** expression in ventral pharyngeal regions**. **(A–U)** Schematics showing ventral views of zebrafish pharyngeal regions summarizing expression of *pkd* genes at 3, 4, and 5 dpf. Black dots indicate expression on pharyngeal cartilage and red dots indicate expression on pharyngeal walls. Cartilage schematics are modified from Schilling and Kimmel (1997), Knight et al. (2003), and Edmunds et al. (2016). Locations of the major cartilaginous elements are shown in panel **(A)**. m, Meckel's cartilage (ventral component of lower jaw); ch, ceratohyal cartilage (derived from the second pharyngeal arch); pq, palatoquadrate cartilage (derived from the first pharyngeal arch, forms the dorsal mandibular cartilage); hs, hyosymplectic cartilage (also derived from the second pharyngeal arch); cb1–5, ceratobranchial cartilage 1–5 (forms the ventral branchial or gill arches).

Mangos et al. (2010) reported expression of *pkd1* in the pharyngeal arches and jaw forming regions at both 52 and 72 h and our data agrees with this. We see strong expression in the pharyngeal arches by 48 h (data not shown) and this expression remains strong at 3 dpf on both the ceratobranchial arches (the more caudal parts of the pharyngeal arches, posterior to the eyes, arrowheads **Figures 12D**, 13A), and also more anteriorly on the hyosymplectic cartilage (medio-lateral and just posterior to the eyes, derived from the second pharyngeal arch, arrowheads **Figures 12D**, 13A). Expression is decreased at 4 and 5 dpf (arrowheads **Figures 12A–F**, 13A–C). Consistent with an earlier report (Bisgrove et al., 2005), we also find *pkd2* expression in the pharyngeal arches at 3 dpf (arrowheads **Figures 12G,J**). In addition, we demonstrate here that strong expression of *pkd2* persists in the pharyngeal arches at 4 dpf (arrowheads **Figures 12H,K**). By 5 dpf, there is also some expression on the pharyngeal walls and the expression in the pharyngeal cartilage has reduced caudally and remains in only the rostral-most pharyngeal cartilage (arrowheads **Figures 12I,L**, 13D–F).

*pkd1l2a* and *pkd1l2b* are also expressed in putative taste buds, although their expression patterns differ from one another and from those of *pkd1* and *pkd2*. We observed *pkd1l2a* expression on the pharyngeal walls and cartilage at all stages examined. In contrast to *pkd1*, which is expressed not only across the entire surface of the ceratobranchial arches but also on the medio-lateral hyosymplectic cartilage at 3 dpf, *pkd1l2a* is expressed only laterally on the ceratobranchial arches and in very few cells medially, in the pharyngeal cavity between the eyes. By 4 dpf, *pkd1l2a* expression is strongest on the ceratohyal cartilage, which is the most rostral and medial component of the pharyngeal cartilage and this expression persists at 5 dpf (arrowheads **Figures 12M–R**, 13G–I). In contrast to *pkd1l2a*, we did not detect expression of *pkd1l2b* on the pharyngeal walls. Instead it is expressed in a few medial cells of the ceratobranchial arches (caudal-most pharyngeal arches) at 3 dpf. This expression spreads laterally by 4 dpf, and is expanded further by 5 dpf such that it is adjacent and caudal to that of *pkd1l2a* at these stages (arrowheads **Figures 12S–X**, 13J–L). At 3 dpf the expression of *pkd2l1* partially overlaps that of *pkd1l2a* and *pkd1l2b* across the ceratobranchial arches, although, like *pkd1l2b, pkd2l1* is not expressed on the pharyngeal walls, it is only expressed on the pharyngeal cartilage. This expression of *pkd2l1* increases and expands laterally across the ceratobranchial arches over the next 2 days (arrowheads **Figures 12Y,Z,A'–D'**, 13M–O).

We also see weak expression of *pkd1b* in the pharynx, probably on the pharyngeal walls at 2, 3, 4, and 5 dpf. By 4 dpf, and persisting at 5 dpf, this gene is also expressed in a few locations on the pharyngeal cartilage (arrowheads **Figures 12E'–J'**, 13P–R). *pkd1l1* is only expressed very transiently in these regions. There is no obvious expression on either the pharyngeal cartilage or the pharyngeal walls at either 3 or 5 dpf. However, there is a transient pulse of expression on both the pharyngeal walls and parts of the pharyngeal cartilage at 4 dpf (arrowheads **Figures 12K'–P'**, 13S–U).

## Discussion

In this paper, we identify seven zebrafish *pkd* genes and confirm their orthologous relationships with *Pkd* genes in other vertebrates using phylogenetic and syntenic analyses. Our bioinformatics analyses strongly suggest that this is the full complement of *pkd* genes in zebrafish and other teleosts and we are confident in our classification/naming of all of these genes, based on our synteny and phylogenetic analyses. The only possible exceptions are zebrafish *pkd1l2a* and *pkd1l2b*. The gene that we have called *pkd1l2b* in zebrafish has much more shared synteny with *pkd1l2a* genes in other teleosts than the gene that we have called zebrafish *pkd1l2a*. However, our phylogenetic analysis suggests that our assignments of *pkd1l2a* and *pkd1l2b* in zebrafish are correct. In addition, the other teleost Pkd1l2a proteins share higher homology with the zebrafish Pkd1l2a polycystin-cation-channel domain than with the Pkd1l2b polycystin-cation-channel domain. Finally, all of the teleost Pkd1l2a proteins, including zebrafish, contain a REJ domain (with the exception of green spotted pufferfish for which we only have a very short protein sequence available) and none of the teleost Pkd1l2b proteins, including zebrafish, contain a REJ domain.

Interestingly, our data suggest that only two *pkd* ohnologs from the whole genome duplication that occurred at the base of the teleosts have been retained: all four teleost genomes that we analyzed have both a *pkd1l2a* and a *pkd1l2b* gene, whereas we have not found *Pkd1l2b* in other vertebrates. In contrast, we found orthologs of all of the other teleost *pkd* genes in at least some other vertebrate lineages.

This limited number of teleost duplicate genes or ohnologs is accompanied by the lack of some *pkd* genes that exist in mammals. Our systematic bioinformatic analyses of all four teleost genomes, demonstrates that they lack *pkd212* and *pkdrej* genes. Given that both of these genes are present in the cartilaginous fish, elephant shark (*Callorhinchus milii*) and the holostei fish, spotted gar (*Lepisosteus oculatus*), it is likely that these two genes have been lost in the teleost lineage. In addition, while we have found putative partial *pkd1l3* genes in teleosts, these sequences lack the region that encodes the polycystin-cation-channel domain that is present in all other *PKD* genes, suggesting that they are no longer bona-fide *PKD* genes. The spotted gar genome also contains a partial *pkd1l3* sequence that is missing this polycystin-cation-channel domain region but has conserved synteny with *pkd1l3* genomic regions in other species. This suggests that *pkd1l3* may be in the process of being lost in the ray-finned fish lineage. In contrast, it is likely that the *Pkd1b* gene has been lost somewhere in the lobe-finned fish lineage as elephant shark, spotted gar, zebrafish, green spotted pufferfish and stickleback genomes all contain this gene, but mouse and human genomes do not (Figures 2, 3; Supplementary Table 5).

Interestingly, our data suggest that *pkd1b* has also undergone relatively rapid evolution in the teleost lineage. This gene appears to be absent in medaka. In addition, stickleback Pkd1b lacks the polycystin-cation-channel domain, suggesting that it is no longer a bona-fide Pkd protein and the polycystin-cation-channel domain is, unusually, not very highly conserved between zebrafish and pufferfish Pkd1b.

It is not clear what the functional significance is of teleosts having fewer *pkd* genes than mammals. It is possible that PKD proteins have additional functions in mammals compared to teleosts, but as not much is currently known about the functions of PKD1L3, PKD2L2, and PKDREJ it is difficult to test this idea. PKD1L3 is implicated in detection of sour-taste in mammals (Huang et al., 2006; Ishimaru et al., 2006) although this may not be the case *in vivo* in mouse, as analysis of a mouse mutant in *Pkd1l3* detected no defects in taste reception (Nelson et al., 2010; Chen et al., 2011; Hofherr and Kottgen, 2011 and references therein). However, as we find expression of all seven zebrafish *pkd* genes in potential taste-bud regions, taste detection is unlikely to be an amniote-specific PKD function. PKDREJ expression has only been detected in sperm (Butscheid et al., 2006; Sutton et al., 2008; Hofherr and Kottgen, 2011) and PKD2L2 is expressed most prominently in mouse testis and oocytes (Veldhuisen et al., 1999; Guo et al., 2000; Taft et al., 2002; Chen et al., 2008; Hofherr and Kottgen, 2011), so it is possible that PKD proteins have a function in germ cells in mammals but not teleosts.

Prior to the start of this project, expression data for zebrafish *pkd* genes was limited to *pkd1* (previously called *pkd1a*), *pkd1b* and *pkd2* (Bisgrove et al., 2005; Obara et al., 2006; Schottenfeld et al., 2007; Mangos et al., 2010; Coxam et al., 2014). In addition, expression of *pkd2l1* in spinal cord KA cells was described during this study (Djenoune et al., 2014). In this paper we confirm and extend these earlier expression analyses and we also provide completely novel information on the expression of *pkd1l1, pkd1l2a*, and *pkd1l2b*. In general, our results are consistent both with earlier studies in zebrafish and with studies of *PKD* gene expression in mammals. The main difference is that, for some of the *pkd* genes, previous reports suggest that they are expressed ubiquitously, whereas we see more specific expression patterns. It is always difficult to distinguish between low-level generalized expression and background staining, so we cannot rule out that there is also low-level ubiquitous expression in these cases.

In most structures we only detect expression of one *pkd1*-like gene and one *pkd2*-like gene, suggesting that these genes encode a heteromeric Pkd1/Pkd2 complex in each case. For example, in the dorsal forerunner cells/KV only *pkd2* and *pkd1l1* are expressed (Figure 6). This is consistent with medaka (Kamura et al., 2011) and mammals, where these two genes are expressed in the equivalent structure, the node (Field et al., 2011; Barratt et al., 2014). This suggests that the expression of these two genes in these related structures, and presumably their function in left/right patterning, is highly conserved between teleosts and mammals. However, the expression of these two genes is not identical in zebrafish. *pkd1l1* is expressed before *pkd2* and while *pkd1l1* is expressed in a condensed group of cells at 12 h, *pkd2* expression resolves into a hollow ring. These expression patterns appear to be at least partially complementary, with the *pkd2* expression surrounding the *pkd1l1* expression (Figures 6D–I). This suggests that the proteins encoded by these genes may only physically interact in a subset of the cells expressing each gene. Interestingly, a recent report suggests that in the mouse node an extracellular domain of PKD1L1 may be required for left-right patterning and that PKD1L1 may be an upstream genetic repressor of PKD2 in this organ (Grimes et al., 2016), which could explain the mainly complementary expression patterns of these two genes in zebrafish.

Similarly, in the developing pronephros we only detect expression of *pkd1* and *pkd2*. This is again, consistent with other vertebrates and with the fact that mutations in these two *PKD* genes, and only these two *PKD* genes, cause ADPKD in humans. While *pkd2* expression in the zebrafish pronephros was published previously (Bisgrove et al., 2005; Obara et al., 2006; Schottenfeld et al., 2007), this is the first report of *pkd1* expression in the developing pronephros. Both *pkd1* and *pkd2* are expressed in the pronephros by 24 h, although the expression of *pkd1* is much stronger than that of *pkd2* (Figures 7A,K, 9O,P,A',B'). The expression of *pkd1* persists until 3 dpf, although expression of *pkd2* is no longer enriched above its otherwise ubiquitous expression by 2 dpf. In contrast, Obara et al. (2006) observed expression of Pkd2 protein in the pronephros at 2 dpf by immunohistochemistry, suggesting that enrichment of Pkd2 protein expression may persist longer than that of the RNA.

In the spinal cord, we confirm the recently reported expression of *pkd2l1* in zebrafish KA cells (Figures 7M,N, 8M–R, 9I,K, 11B,C,G–I'). This expression is also conserved in mouse (Huang et al., 2006; Orts-Del'immagine et al., 2012; Petracca et al., 2016). Excitingly, we identify the potential partner of Pkd2l1 in these specialized neurons as Pkd1l2a (Figures 7G,H, 8G–L, 9E,G, 11A,C–F'). This is also consistent with a very recent report in mouse (Petracca et al., 2016). Our data suggests that all KA′ and KA″ cells express both *pkd1l2a* and *pkd2l1* (Figure 11). We also see expression of both of these genes in a very small number of more dorsal and lateral spinal neurons that are likely to be V2b neurons (Figures 8G,I,L–N, 11A,B). This is consistent with the previous report from Djenoune et al. (2014) as they also saw a small number of dorsal cells that did not contact the central canal and might, therefore, be cells other than KAs.

We also observe broad expression of *pkd1b* in the spinal cord (Figures 8A–F), consistent with an earlier report (Mangos et al., 2010) and weak expression of *pkd2* in the floor plate (Figures 9Y,Z,A'). Our *mindbomb* mutant results (Figure 9) suggest that the broad expression of *pkd1b* is in progenitor cells and that this gene is also expressed in the floor plate. This suggests that a Pkd2/Pkd1b heterocomplex may have a function in the floor plate. In mouse, *Pkd1* is also strongly expressed in the spinal cord (Guillaume et al., 1999), suggesting that there may be a conserved role for Pkd1 proteins in this region of the CNS. Interestingly, mouse PKD1 expression also seems to be enriched in the floor plate region (Guillaume et al., 1999). In contrast, only very low to undetectable spinal cord expression of *Pkd2* has been reported in mouse (Guillaume and Trudel, 2000). Given our results in zebrafish, it would be interesting to examine more closely if there is any enrichment of PKD2 in the mouse floor plate.

In contrast to the spinal cord, and also to some previous reports (e.g., Bisgrove et al., 2005; Mangos et al., 2010; Coxam et al., 2014), with the exceptions of *pkd2l1* and *pkd1l2a* expression in KA cells, which extends into the caudal hindbrain (Djenoune et al., 2014; Figures 7G,H,M,N, insets in Figures 12M–O,Y,Z,A') and *pkd1b* expression in the floor plate in the hindbrain (Figures 7C,D, insets in Figures 12E'–G') we do not see substantial expression of any of the *pkd* genes in the zebrafish brain at the stages that we examined. Due to the tubular structure of the brain, it is prone to probe trapping, particularly in the folds between the forebrain and midbrain and midbrain/hindbrain boundary, which often causes background expression. While we cannot rule out that some of the other genes are expressed broadly or ubiquitously in the brain, we think that any staining is more likely to be background rather than specific expression.

Our experiments also detected expression of *pkd1l1* and *pkd2l1* in the ear, and of *pkd1b, pkd1l1, pkd1l2a, pkd1l2b*, and *pkd2l1* in the retina, suggesting that these genes may be important for development of these crucial sensory organs. In addition, as mentioned above, we observe dynamic expression of all seven zebrafish *pkd* genes in regions that may correspond to taste receptors. This is in contrast to mouse, where only *Pkd1l3* and *Pkd1l2* have been described as being expressed in these cell types (Huang et al., 2006; LopezJimenez et al., 2006). Notably, LopezJimenez and colleagues did not observe any expression of *Pkd1, Pkd2*, or *Pkd1l1* in mouse taste receptors (LopezJimenez et al., 2006). It will be interesting in future studies to confirm whether all of the *pkd* expression in zebrafish pharyngeal regions corresponds to taste receptors and which *pkd* genes are co-expressed in particular subsets of these cells. If, indeed, *pkd* gene expression demarcates different types of receptor cells, these additional *pkd* expression domains in zebrafish might be consistent with suggestions that teleosts have relatively large numbers of different taste receptors (Okada, 2015).

We also observe expression of *pkd1* in neuromasts, although interestingly, we did not find expression of either of the *pkd2*-like genes in these cells. Given that zebrafish neuromasts are deposited by the migrating lateral line primordium, it is intriguing that the large extracellular domain of Pkd1 has been implicated in both cell migration and adhesion (Streets et al., 2003; Castelli et al., 2015).

In addition, we found expression of *pkd2*, and only *pkd2*, in the rostral-ventral somites (Figures 8D', 10P–R), suggesting that this gene is expressed in a subset of muscle cells. Interestingly, in *Drosophila*, Pkd2 is required for optimal contractility of smooth muscle cells (Gao et al., 2004), suggesting that it may have a similar role in zebrafish muscle. Potentially consistent with this idea, there is evidence that Pkd2 physically interacts with Tropomyosin I (Li et al., 2003a) and skeletal isoforms of Troponin I (Li et al., 2003b).

Finally, we also observe expression of *pkd1* in the pectoral fins, as previously reported (Mangos et al., 2010). However, in contrast to this paper, we do not detect expression of *pkd1* in the caudal notochord or around the KV. Unfortunately the authors of this paper do not report which region of *pkd1* they used to make their RNA *in situ* hybridization probe, but as the sequence that their ATG start codon morpholino is designed against is not included in our new *pkd1* transcript, it is possible that their probe contained sequence that recognized other genes in addition to *pkd1*.

In conclusion, in this paper we identify and provide a comprehensive description of the zebrafish family of *pkd* genes and their expression domains during embryogenesis and larval stages. Our data suggest that different pairs of Pkd1-like and Pkd2-like proteins have specific functions during vertebrate development. In particular, that Pkd2 and Pkd1l1 may be required for setting up correct left-right patterning, that Pkd1 and Pkd2 may be required for correct kidney development, that Pkd1l1 and Pkd2l1 may be required for ear development and that Pkd1l2a and Pkd2l1 may be required for correct function of spinal KA cells. We also identify expression of all seven *pkd* genes in potential taste bud-forming regions and of five *pkd* genes in the retina. Interestingly, we also find expression of single *pkd1* or *pkd2* genes in certain structures, such as *pkd1* in neuromasts and pectoral fins and *pkd2* in somites, suggesting that proteins encoded by these genes may also have functions independent of Pkd1/Pkd2 heterocomplexes. Given the importance of PKD proteins for many different aspects of vertebrate development and physiology, this description of the full complement of zebrafish *pkd* genes and their expression in different tissues and organs is a fundamental first step to characterizing the functional roles of these biologically vital proteins.

## Author contributions

SE performed most of the experiments and analyses in the paper, including all the bioinformatics analyses, the synteny, and phylogenetic analyses, most of the cloning (including all of the inverse PCR), some of the *in situ* hybridizations and photography, PC performed and photographed some *in situ* hybridization experiments, AS also performed some *in situ* hybridization experiments, SB performed the double-labeling experiments and took some of the photographs, KL and SE designed and directed the study and wrote the paper. All authors read and approved the final manuscript.

## Funding

This work was funded by HFSP grant RGP063 and NIH NINDS R21NS073979 to KL.

### Conflict of interest statement

The authors declare that the research was conducted in the absence of any commercial or financial relationships that could be construed as a potential conflict of interest.
