# Supplementary material for: Identification and Expression Analysis of the Complete Family of Zebrafish pkd Genes
Source: Front Cell Dev Biol. 2017 Feb 21;5:5. doi: 10.3389/fcell.2017.00005 (PMC5318412; doi:10.3389/fcell.2017.00005)
Supplement: Supplementary file 1 [file DataSheet1.pdf]

Supplementary Material

Identification and Expression Analysis of the Complete Family of Zebrafish *pkd* Genes

Samantha J. England, Paul C. Campbell, Santanu Banerjee, Annika J. Swanson, and Katharine E. Lewis*

*** Correspondence:** Professor K. E. Lewis. Email: kelewi02@syr.edu

1 Supplementary Figures and Tables

1.1 Supplementary Figures

```
tni_Pkd1l1    2049  LTATLVYHLYYLYRSVLILEVVELL-QE-RRGHVDVSLLATCEQHVRSLCGVTLFLLLLKSAAALRVFR  2125
ola_Pkd1l1    2470  VIVSLVYYVHYVYHPTMVMEVAEQLRRN-HREHIDVSTLANSEQFSRTLRGIILFLLAVKCVTVVRLNR  2537
hsa_PKD1L1    2566  VGVSLTYYAVSGHLVTLAGDVTNQFHRGLCRAFMDLTLMASWNQRARWLRGILLFLFTLKCVYLPGIQN  2634
mmu_PKD1L1    2239  VGVAIAYYAASGHLTTLAVNITDQFHKGLYQRLVDIGLMVSWHQRARCLQGILLFLWMLKYVHLLSSLS  2307
cmi_Pkd1l1    2064  IVISLFYYIYYVYYFVLAVDIIDRLHSGYFKAFVDMSFISAWNQRKRFLHGLMLFLLLLKSISLLRINR  2132
dre_Pkd1l1    2067  IICSLLLYICSMYKLQLKTEVIDFLKKEDFKLLVDLSSIFFCEQLSQSLLGIVVNLLLLKCLHLLRLNK  2135
loc_Pkd1l1    2093  ILISLLYYACSLYNYILTVESIDHLQRENFKAFVDLSLLSSWEQLRRGLHGLMVSLLLVKCVFVLRVNK  2161
dre_Pkd1l2b   1688  IILSWSSLSLFIQKTVQGNIDIQYYQKH-KNQFPSFHDTAAVDATLRYVMAFLVLLGCVKLWHLLRLSP  1755
tni_Pkd1l2b   2177  IVLSWSAVAVFIKRTLTGNRDMTYYQNN-RTQFASFYETAMADSTLRYLIAFLVLLSTLKLWRLLRLNP  2244
gac_Pkd1l2b   1458  ILLSWSAVAVFVKRTLLGNRDMTYYENH-KHQFASFYETATADSALQYLVAFLVLLATFKLWHLLRLNP  1525
ola_Pkd1l2b   2229  ILLSMSAVAIFIRRTLLGNRDVAFYQTH-RDQFASFYDTATADLQLQYLIAFLVLLATVKCWHLLRLNP  2296
mmu_PKD1L2    2231  ILASWSALVVFVKRTILADRDLQRYREH-RDEGISFSETAAADAALGYIIAFLVLLSTVKLWHLLRLNP  2298
cmi_Pkd1l2    2243  IMISWSAFSVFIKRTLLGKRDIDHYHNH-REGFVSFYETAMADAVMGYLIAFLVLLATVKLWHLLRLNP  2310
loc_Pkd1l2    2229  ILLSWSALSVFIKRTLLGNRDMQYYQDH-RDEFASFYETAAADAVLGYLIAFLVLLATVKLWHLLRLNP  2296
ola_Pkd1l2a   2243  IFLSWSALSVFIKRTLLGQRDVSQYENN-KDQFVSFHETAQADAVLGYLIAFLVLLATVKLWHLLRLNP  2310
dre_Pkd1l2a   2265  IILSWSALSVFVKRTILGNRDIEFYQNN-KDMFVSFNETATADAVLGYLIAFLVLLATIKLWHLLRLNP  2332
tni_Pkd1l2a    501  IILSWSALSVFIKRTLLGKRDIEYYQKN-KDQFASFHETAKADAVLGYLIAFLVLLATIKLWHLLRLNP   568
gac_Pkd1l2a   2250  ILLSWSALSVFIKRTVLGKRDMEYYQDN-KDQYASFHETAKADAVLGYLIAFLVLLATIKLWHLLRLNP  2317
hsa_PKD1L3    1508  ILISFILLGLDMKSISLHKKNMARYRDD-QDRFISFYEAVKVNSAATHLVGFPVLLATVQLWNLLRHSP  1575
mmu_PKD1L3    1948  VLISFSILGLSMQSLSLLHKKMQQYHCD-RDRFISFYEALRVNSAVTHLRGFLLLFATVRVWDLLRHHA  2015
loc_Pkdrej    1797  NCVLLTAVSFYMYKIRLSSDMLHFYTVN-KQSFIPFHVLASLDKALKTTLGLLAFFSILKTLRYASCLY  1864
cmi_Pkdrej    1812  VVLLLVTILLYVSKFILARNVINYYMKN-PISFIPFHVISALDQLLRFNVAFLILITILKTLRYTRFLY  1879
hsa_PKDREJ    2006  KCIFTVLIVLFLRKHFLATGIIRFYLSN-PEDFIPFHAVSQVDHIMRIILGFLLFLTILKTLRYSRFFY  2073
mmu_PKDREJ    1879  KCMFALLIVLFFWKYFLATKMVQLYLAD-PEAFIPFHAVSRVDHFMRIILAFLLFLTILKTLRYSRFFY  1946
hsa_PKD1      3943  VALTAATALVRLAQLGAADRQWTRFVRGRPRRFTSFDQVAQLSSAARGLAASLLFLLLVKAAQQLRFVR  4011
mmu_PKD1      3932  VILTAATGLVRLAQLGIADRQWTHFVQDHPRHFTSFDQVAQLGSVARGLAASLLFLLLVKAAQQLRFVR  4000
dre_Pkd1      3909  LLLAVCVASLHLSRSVVSAQQWRAFLQH-RDAFTDFFPLAQQNQLLTQMSATLLFLLVLKASHQLRFLR  3976
ola_Pkd1       339  LSVAVCVCGLHLSRCVTAGQQWELFLKQPQISFTDFHPLAQKSQLFTVMAAMLLFVLVLKASHQLRFLR   407
tni_Pkd1      4135  LALAACVCGLHLSRCATATQLWALHSKHPHDGFTDFHPLARRTQLYTLAAALLLFVLVLKVGSQLRFLR  4203
gac_Pkd1      3978  LVLAASVCGLHLSRCAAATQQWASYLRHPRDAFTDFYPVARQSQMYTAASALLLFVLVLKASHQLRFLR  4046
loc_Pkd1      3993  IALSLSTVVVHLSRVSLADRQWDTFLQH-REGFTDFYKFAFQSTVFTELAAVLLFLLILKVSHQLRFLR  4060
cmi_Pkd1      3917  IVLSACCVAVHLWRAKLADRQWLTYRAH-PDGFPNFYHVASLSYLFTSLAALLLFLLTIKTARQVRFIR  3984
loc_Pkd1b     4087  VLLALGVAALHFAFLSLSASLLRHHCHN-RHSFTNFHTAVALSEASSSLSAILLTILILKNVRQLRFVR  4154
cmi_Pkd1b     3851  ILLCVLTSALHFGRLSSADVQFEHYKQN-PWVFTNFYHVAFLAEVVTDLSAVLLTILTVKIVRQLRFVR  3918
dre_Pkd1b     3370  ALLALAAAVLRLYFLYTTTVCISRYRSI-PSSFADFHSAATVARKSNQLSAILLTLLALKMVGILQFVR  3437
tni_Pkd1b     3178  GLLSMTTAILQFCFQAHARSCISKLQNQ-GESFIDFHRAACLAQGCSQSGAILLTVLILKLLGTLRFVR  3245
```

Supplementary Figure 1: Alignment of polycystin-cation-channel domain from different vertebrate PKD1-like proteins.

Comparison of the amino acid sequences for the polycystin-cation-channel domains of human (*Homo sapiens*, *hsa*), mouse (*Mus musculus*, *mmu*), zebrafish (*Danio rerio, dre*), medaka (*Oryzias latipes, ola)*, green spotted pufferfish (*Tetraodon nigroviridis, tni),* stickleback *(Gasterosteus aculeatus, gac)*, spotted gar *(Lepisosteus oculatus, loc)* and elephant shark (*Callorhinchus milii, cmi)* PKD1-like proteins using Clustal Omega (version 1.2.3). This figure shows the region of the polycystin-cation-channel domain that is present in all of the proteins and was used to construct the phylogenetic tree in Fig. 3A. Different families of PKD1-like proteins are color-coded with the same color. This same color-coding is used in Figure 3. Numbers on either side of each sequence, indicate amino acid positions in the full-length sequences of each protein.

```
dme_Pkd2      413  SIQANMSFHH-LEGRVFLYENLLLGPPRLRQIRVRKESCYVNDAFIRYFNTCYAAYSSGA  471
hsa_PKD2L2    103  SWYNNQQLYNLKNSSRIYYENILLGVPRVRQLKVRNNTCKVYSSFQSLMSECYGKYTSAN  162
mmu_PKD2L2    103  SWYGNKQLYSVKNSSRIYYENVLLGIPRVRQLRVRNNTCKVYPAFQSLVSDCYSKYTVEN  162
loc_Pkd2l2     99  DRFPKKSLD--ENESFIYFENVLLGVPRIRQVKVTNTSCSVHGSFAFE-MECYDAYLTLK  155
cmi_Pkd2l2     83  EWYDGQPLS--SKESYIYYENILLGVPQIRQVRVRNNTCSVLKYFRDTFKDCFNEYSLAN  140
hsa_PKD2L1    173  KWYNNQSLG-HGSHSFIYYENMLLGVPRLRQLKVRNDSCVVHEDFREDILSCYDVYSPDK  231
mmu_PKD2L1    173  KWYNNQSLG-RGSHSFIYYENLLLGAPRLRQLRVRNDSCVVHEDFREDILNCYDVYSPDK  231
cmi_Pkd2l1    177  KWYNNDSFS-RGPHSFIYYENMVLGVPRIRQIKVMNNSCVVHKDFREDITGCYNVYSLDN  235
dre_Pkd2l1    165  KWYNDEPLE-NGNQSFIYYENLLLGVPRVRQLKVKQNSCKVPTDFKKEISDCFDVYNIKK  223
loc_Pkd2l1    195  KWYNNQSLS-NGPHSFIYYENMLLGVPRVRQIKVQNNSCKVHRDFRADILGCYDVYDPQK  253
ola_Pkd2l1    175  KWYNQQSLN-NGNQSFIYYENMLLGVPRMRQIKMKNNSCKVHQDFKDEIFGCYDVYDEKK  233
tni_Pkd2l1    197  KWYNDQPLT-TEDKSFIYYENMLLGVPRMRQIKILNNSCKVHSDFKDEITGCFDVYNDKK  255
gac_Pkd2l1    181  KWYNNQSLN-RGEQSFIYYENMLLGVPRMRQIKIMNNSCNVHKDFKDEITGCFDVYNDKK  239
loc_Pkd2      295  VWYNNRTLP--ENRSWIYHESLLLGAPRLRQLKVRNESCVVHEDLRDTVQDCYGAYSPGH  352
ola_Pkd2      293  VWYNNQSLP--GNQSFIYYENILLGVPRLRQVRVHNETCSIHEDLRDEVQGCYGVYTPSN  350
dre_Pkd2      266  LWYNNKSLP--ENQSLIYYENLLLGVPRLRQLRVRNESCSVHEDLRDEVYDCYNVYSPAN  323
tni_Pkd2      258  VWYNNQSLP--ENQSLIYYENLLLGVPRLRQVKVRNESCSVHQDLLGEALECYNLYSPSN  315
gac_Pkd2      239  VWYNNKSLP--ENQSLIYYENLLLGVPRLRQLKVRNESCSVHEDLRDEVLDCYNMYTPTN  296
cmi_Pkd2      230  LWYNNETMT--ENKSFIYYENLLLGVPRMRQLKVKNESCKIHEDLKDEIKECYDVYSFAN  287
hsa_PKD2      198  MQPSNQTEA--DNRSFIFYENLLLGVPRIRQLRVRNGSCSIPQDLRDEIKECYDVYSVSS  255
mmu_PKD2      242  AQTSNHTQA--DNRSFIFYENLLLGVPRLRQLRVRNGSCSIPQDLRDEIKECYDVYSVSS  299

dme_Pkd2      472  EDRKPMHKG--S--PFRTMHDLDSTPIWTVLAFYRTGGYTVNLDYDKDRNVKIINDLKDI  527
hsa_PKD2L2    163  EDLSNFGLQINTEWRYSTSN-TNSPWHWGFLGVYRNGGYIFTLSKSKSETKNKFIDLRLN  221
mmu_PKD2L2    163  EDFSDFGLKRNPEWTHTPSS-RTAPWHWGFVGVYRDGGYIVTLSKSKSETKAKFVDLRLN  221
loc_Pkd2l2    156  EDTASFGPMNSTAWIYSPPSSVV-DIHWGWVSTYSGGGFYLDLEKAKEKNADKIRTLRSN  214
cmi_Pkd2l2    141  EDHSAFGLQTGSEWEYTVPNSQFELRHWGKISKYRNGGYILDLDRQKEESAKKIKHLKSN  200
hsa_PKD2L1    232  EEQLPFGPFNGTAWTYHSQDELGGFSHWGRLTSYSGGGYYLDLPGSRQGSAEALRALQEG  291
mmu_PKD2L1    232  EDQLPFGPQNGTAWTYHSQNELGGSSHWGRLTSYSGGGYYLDLPGSRQASAEALQGLQEG  291
cmi_Pkd2l1    236  ENKAPFGLQNGTAWTYSSEEQLGGSSHWGKLTVYSGGGYYQDLKSNMVESSAILQDLKDN  295
dre_Pkd2l1    224  EDDGAFGLINGTAWQYHSEKEIKGSSHWGLLTTYSGAGFYQDLTTTRDESAALLEDLREN  283
loc_Pkd2l1    254  EDTVAFGLINGTAWQYHTEDQMNGSSQWGLLATYTGGGYYQDLKMTRDESADILMTLKNN  313
ola_Pkd2l1    234  EDDLNFGLINGTAWTYHTEKEMRGSSHWGLLSTYSGAGYYQDLDRTRERSAEILSTLMDN  293
tni_Pkd2l1    256  EDYFNFGLINGTAWSYHTEKEIKGSSHWGLLTTYSGAGYYQDLRRTKEESAAILKDLLDN  315
gac_Pkd2l1    240  EDGLSFGLINGTAWTYHTEKEVKGSSHWGLLTTYSGAGYYQDLSRTKDESAIILGELVDN  299
loc_Pkd2      353  EDTSPFGLLNGTAWIYTNEADLNGSSYWGLIATYSGGGYYLDLSRTRAETAAQIQTLKGN  412
ola_Pkd2      351  EDNSPFGPQNGTAWVHTTESKMNASTHWGQVSKYGGGGYYQDLSRTREESGLQLQFLKDH  410
dre_Pkd2      324  EDKAPFGPKNGTAWRFKDESSLGESSYWGQVSTYGGGGYYQDLSRTREKSANQLQELKNN  383
tni_Pkd2      316  EDTASFGPKNGTAWVYATETEVNGSSHWGQVSKYGGGGYYQDLSPTREGSLVQLQLLKKC  375
gac_Pkd2      297  EERASFGPKIGTAWVYTAESDMDGGGYVGQISKYGGGGYYQDLSRTKEESAAQLQFLKDQ  356
cmi_Pkd2      288  EDSAPFGPKDGTAWIYTPEKDLKANRYWGLIATYSGGGYSLNLGRRRDETAAKFKVLKEN  347
hsa_PKD2      256  EDRAPFGPRNGTAWIYTSEKDLNGSSHWGIIATYSGAGYYLDLSRTREETAAQVASLKKN  315
mmu_PKD2      300  EDRAPFGPRNGTAWMYTSEKELNGSSHWGIIASYSGAGYYLDLSRTREETAAQLAGLRRN  359

dme_Pkd2      528  HWLDRGSRLCLVEFNLFNENTDIFQSIKLIAEIPPTGGVIPQAHLQTVKMYSFFTDRSML  587
hsa_PKD2L2    222  SWITRGTRVIFIDFSLYNANVNLFCIIRLVAEFPATGGILTSWQFYSVKLLRYVSYYDYF  281
mmu_PKD2L2    222  NWISRGTRAVFIDFSLYNANVNLFCIIRLLAEFPATGGLLTSWQFYSVKLLRYVSYYDYF  281
loc_Pkd2l2    215  RWLTRGSRAVFIDFSVYNSNINLFCVVRLVAELPATGGVLTSWQFYSVKLLRYVSSVDYV  274
cmi_Pkd2l2    201  LWLDLGSRAVFIDFSVYNANVNLFCALRLLVEFPATGGATTSWHIYTVKLLRYVSTLDYF  260
hsa_PKD2L1    292  LWLDRGTRVVFIDFSVYNANINLFCVLRLVVEFPATGGAIPSWQIRTVKLIRYVSNWDFF  351
mmu_PKD2L1    292  LWLDRGTRVVFIDFSVYNANINLFCILRLVVEFPATGGTIPSWQIRTVKLIRYVNNWDFF  351
cmi_Pkd2l1    296  LWLDRGTRAVFVDLSVYNANINLFCIIRLVVEFPATGGAVTSWQFRTVKLIRYVNNWDYF  355
dre_Pkd2l1    284  LWLDRGTRVLFIDFTAYNANINLFCVIRLVVEFPATGGAIPSYQIRTVKLIRYVTTWDYF  343
loc_Pkd2l1    314  LWLDRGTRAVFVDFSAYNANINLFSVISLLVEFPATGGAIPSYQIRTVKLIRYINNWDYF  373
ola_Pkd2l1    294  CWLGRGTRVVFIDFSAYNANINMFCVISLMVEFPATGGAVPSYQIRTVKLLRYVTRWDYF  353
tni_Pkd2l1    316  LWLDRGTRVIFVDFSTYNANINMFCIIRLVVEFPATGGAIPSYQIRTVKLIRYISVWDYF  375
gac_Pkd2l1    300  LWLDRGTRAAFLDFSAYNANINMFCVIRLVVEFPATGGAIPSYQIRTVKLIRYINYWDYF  359
loc_Pkd2      413  LWLDRGSRAVFIDFSVYNANVNLFCIVRLVVEFPATGGAVTSWQLQTVKLNRYVSNVDYA  472
ola_Pkd2      411  LWLDRGTRAIFLDFSVYNGNVNLVCIARLLVEFPATGGVLTSWQFQTLRLIKYVSSWDYF  470
dre_Pkd2      384  LWLDRGTRAVFLDFSIYNGNVNLFCIVRLLVEFPATGGAVPSWQFQTVRLLRYVSSWDYF  443
tni_Pkd2      376  LWLDRGTRAVFLDFSVYNGNINLFCIVRLLVEFPATGGVLTSWRFQTVRLIRYVSSWDYF  435
gac_Pkd2      357  LWLDRGTRAVFLDFSVYNGNINLFCIARLLVEFPATGGVVTSWQFQTVRLIRYVSSWDYF  416
cmi_Pkd2      348  LWLDRGTRVVFIDFTVYNANINLFCVIRLVVEFPATGGALSSWQFQTVKLIRYISSFDFF  407
hsa_PKD2      316  VWLDRGTRATFIDFSVYNANINLFCVVRLLVEFPATGGVIPSWQFQPLKLIRYVTTFDFF  375
mmu_PKD2      360  FWLDRGTRAAFIDFSVYNANINLFCVVRLLAEFPATGGVVPSWQFQPVKLIRYVTAFDFF  419
```

```
dme_Pkd2      588   MTVIYIFWYIMVIYYTIYEITEIRKSGIKIYFCSMLNILDCAILLGCYLALVYNIWHSFK   647
hsa_PKD2L2    282   IASCEITFCIFLFVFTTQEVKKIKEFKS-AYFKSIWNWLELLLLLLCFVAVSFNTYYNVQ   340
mmu_PKD2L2    282   IASCEVIFCIFLFVFIIQELRKVNEFKS-AYFRSVWNWLEMLLLLLCFLAVSFYAYCNMQ   340
loc_Pkd2l2    275   LALCEVVFCLFIIAFGFQECVELYQKRC-SYFIEFWNCLELILIVMSVVAISFNVYRTSE   333
cmi_Pkd2l2    261   LGACEILFCLFILNYLGQELIEMYELKL-RYFQSFWNCLDFLIVLLSLLAIVFSIYRTAK   319
hsa_PKD2L1    352   IVGCEVIFCVFIFYYVVEEILELHIHRL-RYLSSIWNILDLVVILLSIVAVGFHIFRTLE   410
mmu_PKD2L1    352   IVGCEVVFCVFIFYYVVEEILEIHLHRL-RYLSSVWNILDLVVILLSIVAVGFHIFRTLE   410
cmi_Pkd2l1    356   IIGCEIVFCVFIFYYIVEEILEIRIHKL-QYIKCIWNCLDLMVILLSLVSIGFHIFRTIK   414
dre_Pkd2l1    344   IIGCEIVFCVFILYYIVEEILEFRIHKL-SHFTSIWNILDIVVILLAVVAIVFSAFRTIK   402
loc_Pkd2l1    374   IAGCEIVFCVFIFYYVVEEILELRIHKL-SYFTSIWNILDVVVILLAIIAIIFNAFRTIK   432
ola_Pkd2l1    354   ILGCEAVFCVFILYYMVEEVLELRIHRF-SYLKSVWNILDIVVILLALVAIAFNVFRTIK   412
tni_Pkd2l1    376   ILGCEVVFCLFIFYYIVEEILELRIHKF-SYFRSIWNILDIVVILLAVVAIIFNVFRTIK   434
gac_Pkd2l1    360   VLGCEVVFCIFILYYVVEEILELRIHKL-SYFNSIWNILDIVVIMLAIVAIIFNIFRTVK   418
loc_Pkd2      473   IAACEIAFCLFVLYYVVEEALEVRIHKL-YYFRNLWNCLDILIIALSVAAIVINIYRTVT   531
ola_Pkd2      471   VGVCEVAFCLFVLYYVVEEVLEIHIHRL-HYFKNLWNCLDVLIVTLSVVAIIMSITRAAM   529
dre_Pkd2      444   VGMCEVSFCLFVLYYLVEEALEIRLHRL-RYFKSLWNCLDVLIVALSVPAIIMNICRTSA   502
tni_Pkd2      436   VGVCEVAFSLFILYYIVEEVLEIRIHRL-HYFRSLWNCLDVLIIILLSVVAVIMNITRTAT   494
gac_Pkd2      417   VGLCEVAFCLFIVYYMVEEVLEVRIHRL-HYFKSLWNCLDVLIVVLSVVAIIMNITRTAM   475
cmi_Pkd2      408   LAACEIAFSIFIFYYIVEEILEIYIHRL-HYFRNLWNCLDIVIIMLSLVAICINIYRMSM   466
hsa_PKD2      376   LAACEIIFCFFIFYYVVEEILEIRIHKL-HYFRSFWNCLDVVIVVLSVVAIGINIYRTSN   434
mmu_PKD2      420   LAACEIIFCFFIIYYVVEEILEIRIHRL-SYFRSFWNCLDVVIVVLSVVAMVINIYRMSN   478

dme_Pkd2      648   VMSLTARA-HSDVTYQSLDVLCFWNIIYVDMMAILAFLVWIKIFKFISFNKTLVQFTTTL   706
hsa_PKD2L2    341   IFLLLGQLLKSTEKYSDFYFLACWHIYYNNIIAITIFFAWIKIFKFISFNKTMSQLSSTL   400
mmu_PKD2L2    341   SFLLLGQLLKNTDSYPDFYFLAYWHIYYNNVIAITIFFAWIKIFKFISFNETMSQLSSTL   400
loc_Pkd2l2    334   VHTLLESLQENPQKYPDFHFLAFWQTQYNNMIAVNVFICWVKVFKYINFNKTMNTLTSTL   393
cmi_Pkd2l2    320   VSLVLEKLLENSFKYPNLYFLAYWQNQYNNMIAITVFIAWIKIFKFISFNETMTQLATTL   379
hsa_PKD2L1    411   VNRLMGKLLQQPNTYADFEFLAFWQTQYNNMNAVNLFFAWIKIFKYISFNKTMTQLSSTL   470
mmu_PKD2L1    411   VNRLMGKLLQQPDTYADFEFLAFWQTQYNNMNAVNLFFAWIKIFKYISFNKTMTQLSSTL   470
cmi_Pkd2l1    415   VNIILGALLKYPEAYADFEFLAFWQTQYNNMNAVNLFFAWIKIFKYISFNKTMTQLSSTL   474
dre_Pkd2l1    403   VDGLLGNLLKQPDIYADFEFLAFWQTQYNNMNAVNLFFAWIKIFKYISFNKTMTQLTSTL   462
loc_Pkd2l1    433   VNSLLGKLLEQPDIYADFEFLAFWQTQYNNMNAVNLFFAWIKIFKYISFNKTMTQLSSTL   492
ola_Pkd2l1    413   VDNLLGKLLENPNIYADFEFLAFWQTQYNNMNAVNLFFAWIKIFKYISFNKTMTQLSTTL   472
tni_Pkd2l1    435   VDNLLGKLLEQPDIYADFEFLAFWQTQYNNMNAVNLFFAWIKVFKYISFNKTMTQLSSTL   494
gac_Pkd2l1    419   VDNLLGKLLEQPGIYADFEFLAFWQTQYNNMNAVNLFFAWIKVFKYISFNKTMTQLSSTL   478
loc_Pkd2      532   VNSLLKKLLEIQDAYPNFQPLAYMQVQFNNMVAMIVFFSWIKLFKFINFNKTMSQLSSTM   591
ola_Pkd2      530   GGDLLKGL-ENYTSHTSFDSLANLQVQFNNMAAVIVFFCWVKLFKFINFNKTMSQLSTTM   588
dre_Pkd2      503   VSHRLHFLLENHSTYPNFEPLARLQVHFNNLAAIIVFLSWVKLFKFINFNKTMNQLSTTM   562
tni_Pkd2      495   VGNLLKGLLENHAAHPSFESLAHLQVQFNNVAAVIVFFSWVKLFKFINLNKTMSQLTTTM   554
gac_Pkd2      476   VSSRLKGLLENHTAHPTFEPLANLQIQFNNMAAVIVFFSWVKLFKFINFNKTMSQLSGTM   535
cmi_Pkd2      467   VNTLLGELLEKQNSYHNFESLAYWQIQFNSVAAVVVFFAWIKIFKFISFNKTMIQLSSTM   526
hsa_PKD2      435   VEVLL-QFLEDQNTFPNFEHLAYWQIQFNNIAAVTVFFVWIKLFKFINFNRTMSQLSTTM   493
mmu_PKD2      479   AEGLL-QFLEDQNSFPNFEHVAYWQIQFNNISAVMVFLVWIKLFKFINFNRTMSQLSTTM   537

dme_Pkd2      707   KRCSKDLAGFSLMFGIVFLAYAQLGLLLFGTKHPDFRNFITSILTMIRMILGDFQYNLIE   766
hsa_PKD2L2    401   SRCVKDIVGFAIMFFIIFFAYAQLGFLVFGSQVDDFSTFQNSIFAQFRIVLGDFNFAGIQ   460
mmu_PKD2L2    401   SRCMKDIVGFAIMFFIIFSAYAQLGFLVFGSQVDDFSTFQNSIFAQFRIVLGDFNFAGIQ   460
loc_Pkd2l2    394   SHCAKDVIGFAVMFFIIFFAYAQLAYLLFGSQVENFSTFKNCVFTQFRIILGDINFNEIE   453
cmi_Pkd2l2    380   QHCAKDIAGFAIMFFIIFFGYAQLGYLIFGAQVESFHTFSNCIFTQFRIILGDFNFAEIE   439
hsa_PKD2L1    471   ARCAKDILGFAVMFFIVFFAYAQLGYLLFGTQVENFSTFIKCIFTQFRIILGDFDYNAID   530
mmu_PKD2L1    471   ARCAKDILGFAIMFFIVFFAYAQLGYLLFGTQVENFSTFVKCIFTQFRIILGDFDYNAID   530
cmi_Pkd2l1    475   ARCAKDILGFAIMFFIVFFAYAQLGYLLFGTQVENFSTFLKCIFTQFRIILGDFNYDEID   534
dre_Pkd2l1    463   ARCALDIFGFAIMFFIVFFAYAQLGYLLFGTEVETFSTFNKCIFTQFRIILGDFDYDAID   522
loc_Pkd2l1    493   ARCAKDILGFAIMFFIVFFAYAQLGYLLFGTEVQTFSTFVKCIFTQFRIILGDFDYDAID   552
ola_Pkd2l1    473   GRCAKDILGFAVMFFIVFFAYAQLGYLLFGTEVDTFSTFVKCIFTQFRIILGDFNFDAID   532
tni_Pkd2l1    495   GRCAKDIVGFAVMFFIVFFAYAQLGYLLFGMEVESFSTFAKCIFTQFRIILGDFDYNAID   554
gac_Pkd2l1    479   GRCAKDILGFAIMFFIVFFAYAQLGYLLFGTEVESFSTFVKCIFTQFRIILGDFDYDAID   538
loc_Pkd2      592   SRCAKDIIGFAIMFLIIFLAYAQLAYLVFGTQVNDFSTFQACIFTQFRIILGDFNFAEIE   651
ola_Pkd2      589   SRCAKDLVGFAIMFFIIFLAYAQLAYLVFGTQVNDFSTFQGSVFTQFRIILGDFDFFEIK   648
dre_Pkd2      563   SRCAKDLMGFAIMFFIVFLAYAQLAYLVFGTQVNDFSTFQACIFTQFRIILGDFDFSEIE   622
tni_Pkd2      555   SRCAKDLVGFAIMFFIIFLAYAQLAYLVFGTQVGDFSTFQASIFTQFRIILGDFEFSEIE   614
gac_Pkd2      536   SRCAKDLVGFAIMFFIIFLAYAQLAYLVFGTQVNDFSTFQASIFTQFRIILGDFEFSEIM   595
cmi_Pkd2      527   SRCAKDILGFAIMFFIVFLAYAQLAYLVFGTQVDDFSTFQTCIFTQFRIILGDFDFIKIE   586
hsa_PKD2      494   SRCAKDLFGFAIMFFIIFLAYAQLAYLVFGTQVDDFSTFQECIFTQFRIILGDINFAEIE   553
mmu_PKD2      538   SRCAKDLFGFTIMFSIIFLAYAQLAYLVFGTQVDDFSTFQECIFTQFRIILGDINFAEIE   597
```

***dme*_Pkd2**	767	QANRVLGPIYFLTYILLVFFILLNMFLAIIMETYNTV	803
***hsa*_PKD2L2**	461	QANPILGPIYFITFIFFVFFVLLNMFLAIINDTYSEV	497
***mmu*_PKD2L2**	461	QANWILGPIYFITFIFFVFFVLLNMFLAIINDTYSEV	497
***loc*_Pkd2l2**	454	HSNRILGPIYFTTFVFFVFFILLNMFLAIINDTYSEV	490
***cmi*_Pkd2l2**	440	KANRILGPIYFTTFVFCIFFVLLNVFLAIINETYAEV	476
***hsa*_PKD2L1**	531	NANRILGPAYFVTYVFFVFFVLLNMFLAIINDTYSEV	567
***mmu*_PKD2L1**	531	NANRILGPVYFVTYVFFVFFVLLNMFLAIINDTYSEV	567
***cmi*_Pkd2l1**	535	NANRILGPIYFVTYVFFVFFVLLNMFLAIINDTYSEV	571
***dre*_Pkd2l1**	523	RANRVLGPIYFFSYVFFVFFVLLNMFLAIINDTYSEV	559
***loc*_Pkd2l1**	553	QANRILGPIYFVTYVFFVFFVLLNMFLAIINDTYSEV	589
***ola*_Pkd2l1**	533	QANRVLGPIYFVTYVFFVFFVLLNMFLAIINDTYSEV	569
***tni*_Pkd2l1**	555	RANRVLGPIYFVTYVFFVFFVLLNMFLAIINDTYTEV	591
***gac*_Pkd2l1**	539	RANRVLGPIYFVTYVFFVFFVLLNMFLAIINDTYSEV	575
***loc*_Pkd2**	652	EANRILGPIYFTTFVFFIFFILLNMFLAIINDTYSEV	688
***ola*_Pkd2**	649	EANPVLGPIYFITFVLFIFFILMNMFLAIINDTYSEV	685
***dre*_Pkd2**	623	EADSVLGPIYFTTFVFFIFMILLNMFLAIINDTYSEV	659
***tni*_Pkd2**	615	EASPVLGPVYFTTFVFFVFFILMNMFLAIINDTYSEV	651
***gac*_Pkd2**	596	EANPVLGPIYYTTFAFFIFFILMNMFLAIINDTYSEV	632
***cmi*_Pkd2**	587	EANRILGPIYFATFVFFIFFILLNMFLAIINDTYSEV	623
***hsa*_PKD2**	554	EANRVLGPIYFTTFVFFMFFILLNMFLAIINDTYSEV	590
***mmu*_PKD2**	598	EANRVLGPLYFTTFVFFMFFILLNMFLAIINDSYSEV	634

Supplementary Figure 2: Alignment of polycystin-cation-channel domain from different PKD2-like proteins.

Comparison of the amino acid sequences for the polycystin-cation-channel domains of human (*Homo sapiens*, *hsa*), mouse (*Mus musculus*, *mmu*), zebrafish (*Danio rerio, dre*), medaka (*Oryzias latipes, ola)*, green spotted pufferfish (*Tetraodon nigroviridis, tni),* stickleback *(Gasterosteus aculeatus, gac)*, spotted gar *(Lepisosteus oculatus, loc)*, elephant shark (*Callorhinchus milii, cmi)* and *Drosophila melanogaster* (*dme*) PKD2-like proteins using Clustal Omega (version 1.2.3). This figure shows the region of the polycystin-cation-channel domain that is present in all of the proteins and was used to construct the phylogenetic tree in Fig. 3B. Different families of PKD2-like proteins are color-coded with the same color. This same color-coding is used in Figure 3B. Numbers on either side of each sequence, indicate amino acid positions in the full-length sequences of each protein.

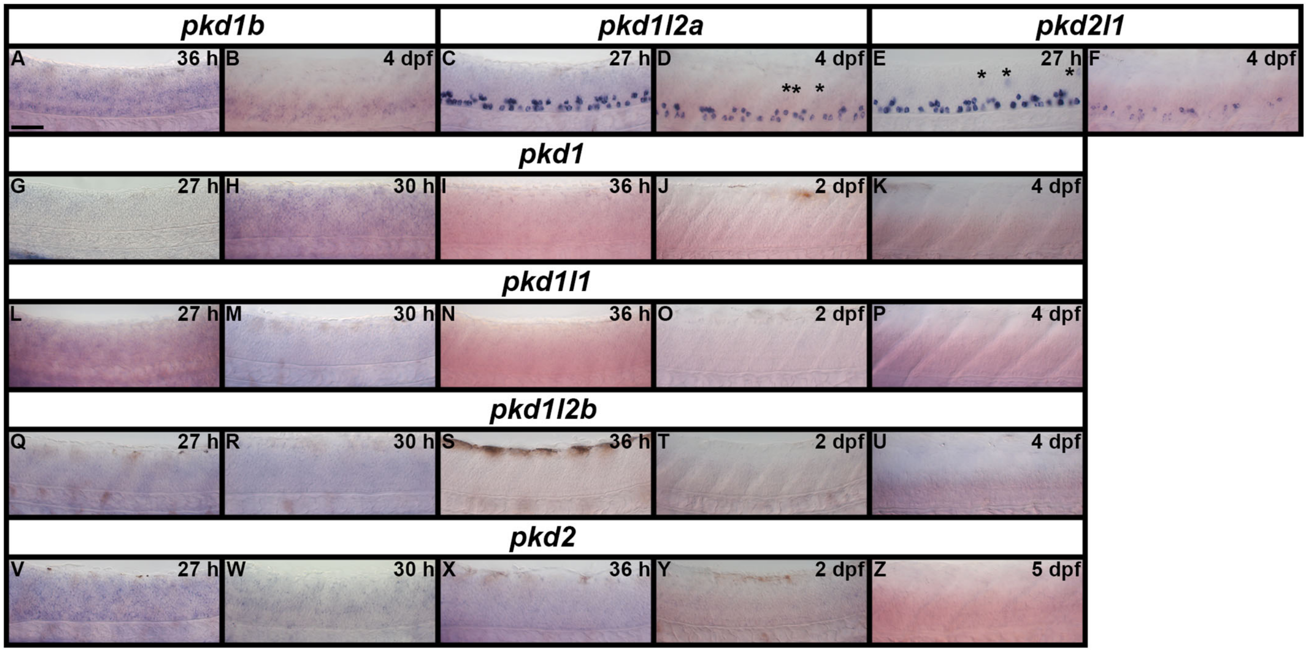

<u>Supplementary Figure 3: Spinal cord expression of zebrafish *pkd* genes</u>

Lateral views of additional stages of *pkd* spinal cord expression not included in other figures. Rostral left, dorsal up. By 36 h, *pkd1b* is expressed in two ventral rows of cells in the spinal cord (A). This expression persists at 4 dpf (B). *pkd1l2a* and *pkd2l1* are co-expressed in KA cells (C-F) and occasional weak more dorsal cells (asterisks D & E). *pkd1*, *pkd1l1*, *pkd1l2b* and *pkd2* are not expressed in spinal cord (G-Z). Scale bar (A) = 50 µm.

1.2 Supplementary Tables

Primer Name	Forward Primer Sequence	Reverse Primer Sequence	Annealing Temperature (°C)	PCR Extension Time (Seconds)	Transcript Exons Mapped
*pkd1*_Map_ Nested Set 1	GTGCAGCACCAACTCTGAG	CATGAAAACACTTGGAGTTGCAC	62.0	100	1-595 bp of transcript[+]^
*pkd1*_Map_ Nested Set 2	CACCAACTCTGAGCCCAATCAC	GAGGACGAGAAGATGACCAGC	68.0	100	1-595 bp of transcript[+]^
*pkd1*_Map_ Set 3	AGGAGCTAGATCTGAGCAACAAC	CCTCTCTGCCATCCTTACTGATC	66.0	60	527-1143 bp of transcript^
*pkd1*_Map_ Set 4	ACGTGTGTGTGTCTCTGGAC	GGATCCATTAACCCTCACTAAAGGGAAC TGTACTCTGGGTATTGTGTGC §	61.0	60	1575-2154 bp of transcript^
*pkd1*_Map_ Set 5	ATTCAGAAAGGGCTCACACTGAC	CAGAGAATTGTGGGAGTTGTGC	69.0	60	2122-2920 bp of transcript^
*pkd1*_Map_ Set 6	AGAGGACAGGAAAGAGCAAGAAG	CAAACGGGACTTCATGAGGATTG	67.0	60	13121-14065 bp of transcript^
*pkd1*_Map_ Set 7	CAATCCTCATGAAGTCCCGTTTG	CTGACATACAATCGCCACAGAAC	68.0	60	14102-15014 bp of transcript^
*pkd1*_Map_ Set 8	TATTCTGGTGGGCTGTTCTGATG	GTCAGTCACTCAACAACAACCTC	66.0	60	15527-16413 bp of transcript^
*pkd1*_Map_ Set 9	GGTCAGTGATCAGCTAGTAGTGC	TTCTGAGGAAGCGCAAACTCTC	65.0	60	17453-18305 bp of transcript^
*pkd1l2a*_Map_ Set 1	GTGCTGATCATAATGCTACGCTTC	CTAAAGAGCGGCCAGACTCAAAC	69.0	60	*pkd1l2a* exons 1-2*
*pkd1l2a*_Map_ Set 2	CTGCTATGAGTTTGTGGAGTTGC	CTGTGCTTCCATGCTGAACTTTG	68.0	60	*pkd1l2a* exons 2-6
*pkd1l2a*_Map_ Set 3	GCTGATCATAATGCTACGCTTCAG	GATATTGAGGACTCGCACCGGAC	69.0	60	*pkd1l2a* exons 3-7
*pkd1l2a*_Map_ Set 4	CAGCCCACAAAACCATCACTATC	CATCATCTGGTGTGTCTCGGAG	69.0	60	*pkd1l2a* exons 6-7
*pkd1l2a*_Map_ Set 5	TCTGAATGAGGTGACGGCAG	CCCATTTGCATTGGTCGATACAG	67.0	100	*pkd1l2a* exons 7-10
*pkd1l2a*_Map_ Set 6	TCTGAATGAGGTGACGGCAG	ATGCTTCTGATGGCTTCTCAGTC	66.0	100	*pkd1l2a* exons 7-13

*pkd1l2a*_Map_ Set 7	CAAATGAAAGAGGACAGCAGCTC	AAAGAACATCAGAGCCCAGAGAG	68.0	60	*pkd1l2a* exons 13-18
*pkd1l2a*_Map_ Set 8	TGTAACGCTCCCTATCCTCTTTC	AATTAACCCTCACTAAAGGGAGCGCCTA CAAAACACACCAC§	67.0	100	*pkd1l2a* exons 13-23
*pkd1l2a*_Map_ Set 9	CTTCAGCACTGTGGTCATCAAC	GAGTACAAGAGCAAAGAAGACTGC	65.0	100	*pkd1l2a* exons 22-29
*pkd1l2a*_Map_ Set 10	GTCAGAGGCCAGTGAAAAGAAAG	AATTAACCCTCACTAAAGGGAGCACCTC ACTTCGATATTGGAAC§	68.0	60	*pkd1l2a* exons 30-34
*pkd1l2a*_Map_ Set 11	TTATGTCGGATTCCTCTGGATGC	TTTCCACTCAGAAAAGCTGACA	67.0	100	*pkd1l2a* exons 33-41
*pkd1l2b*_Map_ Nested Set 1	ATCCAGAATGATGAAACCCAGC	CACGGTCACAAACTCATAGCAG	68.0	100	*pkd1l2b* exon 1[+]
*pkd1l2b*_Map_ Nested Set 2	GGACTGGTGGATTGGACTTGTG	CAAAAGACTGCTGGTTCTCTGTG	68.0	100	*pkd1l2b* exon 1[+]
*pkd1l2b*_Map_ Set 3	ATCCAGAATGATGAAACCCAGC	TGGAGCGGTTCAGAGTCCTC	66.0	60	*pkd1l2b* exons 2-9
*pkd1l2b*_Map_ Set 4	TGAGCAAAGATGAGGTGAAGAGC	AACCCGTGATGAAGTGGAAGATC	70.0	100	*pkd1l2b* exons 9-18
*pkd1l2b*_Map_ Set 5	TCCATATGTTACCCAGTGTCTGTG	TGAGGAGGCTCAGACTTCTTC	66.0	60	*pkd1l2b* exons 18-21
*pkd1l2b*_Map_ Set 6	TGCTGCTCTGCTCTATGTTGAC	ACTTCTTCATCCTCGGCTTCAAC	68.0	60	*pkd1l2b* exons 22-23
*pkd1l2b*_Map_ Set 7	CTTCACCGAGCCTGTAAAAGTTG	GGCCATGAGAAAGGACATAAAGC	69.0	60	*pkd1l2b* exons 24-28
*pkd1l2b*_Map_ Set 8	CCGTGTACAATGCTAATGTCGATC	CACGTCAATGACCGGGATTATG	69.0	60	*pkd1l2b* exons 29-34

Supplementary Table 1. PCR Primers for mapping *pkd1*, *pkd1l2a* and *pkd1l2b* mRNA transcripts

PCR primers and conditions used to amplify and sequence overlapping fragments of zebrafish *pkd1*, *pkd1l2a* and *pkd1l2b* transcripts (genes for which annotations in Ensembl are incomplete). Column 1 lists names given to each primer set. Columns 2 and 3 list PCR primers used to map transcript region indicated in column 6. Columns 4 and 5 list annealing temperatures and extension times of PCR protocols used in each case. The rest of the protocol is provided in materials and methods. *These primers were used on a circularized cDNA product following inverse PCR to generate 5' sequence. [+]These primers were used in a series of nested PCR reactions. Nested Set 1 primers were used on a circularized cDNA product following inverse PCR to generate 5' sequence. Nested Set 2 primers were used in reactions seeded with the Nested Set 1 PCR product. See materials and methods for details. § These reverse primers contain sequence for T3 RNA Polymerase

promoter at their 5' end, since they were also used to generate *in situ* hybridization riboprobes (see Supplementary Table 3 for riboprobe primers). ^ Since some of our mapped sequence data is not present in the current Ensembl chromosome 1 genomic sequence (GRCz10), we cannot map all exon boundaries for *pkd1* and so instead we show the base pair locations within the mapped mRNA transcript that were amplified and sequenced by these primers (see Figure 1 text for further information).

Gene-Specific Primer	Primer Sequence
pkd1	CGGCACTCCTTCATGACGAG
pkd1l2a	CTGTGGAGATGTTTGTGAAGGAG
pkd1l2b	GTCCAAGATGCGATTGTCAGATG

Supplementary Table 2. PCR Primers for Inverse PCR

Sequences of gene-specific reverse primers used in first strand cDNA synthesis during inverse PCR for *pkd1, pkd1l2a* and *pkd1l2b* transcripts (see materials and methods for further details).

Gene Name	Forward Primer Sequence	Reverse Primer Sequence	PCR Product Size (bp)
pkd1 – Set 1*	ACGTGTGTGTGTCTCTGGAC	GGATCC**ATTAACCCTCACTAAAGGGA**ACTGTACTCTGGGTATTGTGTGC	580
pkd1 – Set 2	CTCCAGCTACATATCAGTGGGAC	**AATTAACCCTCACTAAAGGGA**ATCTAGAACCAGCGTCTCGTTAC	1007
pkd1b	GTTCTTGGACTGTGAGCATTGTC	**AATTAACCCTCACTAAAGGGA**TCATGGGAGGTCAAAAGAGTCAC	963
pkd1l1 – Set 1	GCGTGTCCCTCTGTCTTACATG	**AATTAACCCTCACTAAAGGGA**GTCCAGTTCCACCAATCATCATG	604
pkd1l1 – Set 2	GTGATTCTCCAGTTTGTCCTGTAC	**AATTAACCCTCACTAAAGGGA**GTTCACAACAATGCCAAGGAGTG	447
pkd1l2a – Set 1	GTCAGAGGCCAGTGAAAAGGAAG	**AATTAACCCTCACTAAAGGGA**GCACCTCACTTCGATATTGGAAC	1136
pkd1l2a – Set 2	CGTGACTGCGCCCAATATATC	**AATTAACCCTCACTAAAGGGA**GCGCCTACAAAACACACCAC	501
pkd1l2b	CTGTTTGGGGTTTAACTGCACTG	**AATTAACCCTCACTAAAGGGA**ATTACCAGTTCCACGGTTTCCTC	982
pkd2	CTTACTCTGAGGTGAAAGCTGAC	**AATTAACCCTCACTAAAGGGA**GTTTTCACAAATAAACACCCCTCAC	878
pkd2l1	CAGAGGCTGGTTCACAAAGTTTC	**AATTAACCCTCACTAAAGGGA**GCCTGATACAGTTGAAATTCCCAG	553

Supplementary Table 3. PCR primers for creating *in situ* hybridization riboprobes

Primer sequences used to generate riboprobes for *in situ* hybridization. Expected PCR product sizes (in base pairs) are indicated in column 4. * indicates primers published in Coxam *et al*., (2014). All other riboprobe primers were designed during this study. T3 RNA polymerase promoter sequence at 5' end of each reverse primer is bold and underlined. *pkd1* – Set 1, *pkd1l1* – Set 1 and *pkd1l2a* – Set 1 primers generated riboprobes that gave the strongest expression in our assays and so were used exclusively in this study. See materials and methods for further information.

Species	Percentage Amino Acid Identity to Full-Length Mouse PKD1L3 Protein
Zebrafish	aa **976-1120** (29%), **1148-1236** (37%), 1258-1316 (39%) and 1529-1601 (38%)
Green Spotted Pufferfish	aa **1004-1127** (34%), **1108-1208** (40%), **1209-1312** (38%) and 1539-1595 (46%)
Medaka	aa **976-1044** (31%), **1037-1116** (40%), **1148-1209** (55%) and **1209-1312** (30%)
Stickleback	aa **1037-1168** (35%), **1113-1209** (33%), **1206-1257** (38%), 1255-1312 (46%) and 1529-1594 (43%)

Supplementary Table 4. Percentage Amino Acid Identity Between Full-Length Mouse PKD1L3 Protein and Teleost Putative *pkd1l3* Orthologs

Results obtained from blasting full-length mouse PKD1L3 against teleost genomes. Regions of the mouse query sequence that have identity with sequences in respective teleost genomes are indicated by amino acid (aa) numbers and percentage amino acid sequence identity is indicated in parentheses. Numbers in bold correspond to regions of the mouse PKD1L3 query sequence that contain at least part of the GPS motif (amino acids 1020-1058) or PLAT/LH2 domain (amino acids 1131-1245).

**Species**	**Current Ensembl Gene Name**	**Ensembl Gene ID**	**Chromosome Location**	**Supporting Evidence**
pkd1				
Spotted gar	*pkd1*	ENSLOCG00000002998	LG13(+): 4728181-4832181	Shares synteny with teleost and mammalian *pkd1* genes (data not shown). Phylogeny (Fig. 3A).
Elephant shark	*pkd1* (two *pkd1* genes in genome)	SINCAMG00000009812	Scaffold_312(+): 34635-113581	Shares synteny with teleost and mammalian *pkd1* genes (data not shown). Phylogeny (Fig. 3A).
pkd1b				
Spotted gar	*pkd1b*	ENSLOCG00000010726	LG10(-): 27484014-27547534	Shares synteny with green spotted pufferfish and stickleback *pkd1b* genes (data not shown). Phylogeny (Fig. 3A).
Elephant shark	*pkd1* (two *pkd1* genes in genome)	SINCAMG00000001332	Scaffold_10(+): 2273648-2326286	Does not share synteny with teleost *pkd1b* genes (data not shown). Formerly called *pkd1* but phylogeny suggests that this gene is *pkd1b* (Fig. 3A).
pkd1l1				
Spotted gar	Novel	ENSLOCG00000013313	LG9(+): 50517659-50554155	Shares synteny with zebrafish *pkd1l1* gene (data not shown). Shares homology with amino acids 205-642, 859-908, 1303-1400 and 1495-1607 of mouse PKD1L1 protein. Phylogeny (Fig. 3A).
Elephant shark	*pkd1l1*	SINCAMG00000004987	Scaffold_55(+): 2766931-2840140	Shares synteny with amniote *PKD1L1* genes (data not shown). Phylogeny (Fig. 3A).
pkd1l2				
Spotted gar	Novel	ENSLOCG00000003938	LG23(+): 6943671-6977736	Shares synteny with all teleost *pkd1l2a* genes (data not shown). Shares homology with amino acids 4-525, 1239-1667 and 1804-2410 of mouse PKD1L2 protein. Phylogeny (Fig. 3A).
Elephant shark	*pkd1l2*	SINCAMG00000003573	Scaffold_12(-): 2515744-2542623	Shares synteny with amniote *PKD1L2* genes (data not shown). Phylogeny (Fig. 3A).

pkd1l3				
Spotted gar	Novel	ENSLOCG00000004480	LG23(+): 8258413-8268971	No homology with the polycystin-cation-channel domains of zebrafish Pkd proteins or mouse PKD1L3 protein. Shares homology with amino acids 1037-1333 and 1525-1594 of mouse PKD1L3 protein (containing the GPS and PLAT/LH2 domains). Shares synteny with mammalian and teleost *PKD1L3* genes (data not shown).
Elephant shark	All *PKD1L3* genes examined in this study are syntenic with the *DHODH* gene. In the elephant shark, *dhodh* is present on Scaffold_12 and is also flanked by the genes *znf821* and *atxn11*, which also surround the mammalian *PKD1L3* genes. Tblastn analysis with either the polycystin-cation-channel domains of zebrafish PKD proteins or the full-length mouse PKD1L3 protein failed to detect any hits adjacent to these genes on Scaffold_12 in the elephant shark genome, although we cannot rule out the possibility that a *pkd1l3* ortholog might exist elsewhere in the genome.			
pkdrej				
Spotted gar	*pkdrej*	ENSLOCG00000016654	LG8(+): 40852661-40859002	Phylogeny (Fig. 3A).
Elephant shark	*pkdrej*	SINCAMG00000010353	Scaffold_35(-): 5337490-5343965	Shares synteny with mammalian *PKDREJ* genes (data not shown). Phylogeny (Fig. 3A).
pkd2				
Spotted gar	*pkd2*	ENSLOCG00000013447	LG4(-): 66726806-66740815	Shares synteny with green spotted pufferfish, stickleback and medaka *pkd2* genes (data not shown). Phylogeny (Fig. 3B).
Elephant shark	*polycystin 2*	SINCAMG00000010189	Scaffold_21(+): 1937754-1973648	Shares synteny with green spotted pufferfish, stickleback and medaka *pkd2* genes (data not shown). Phylogeny (Fig. 3B).
pkd2l1				
Spotted gar	*pkd2l1*	ENSLOCG00000011334	LG5(+): 28795999-28813061	Shares synteny with all teleost *pkd2l1* genes examined (data not shown). Phylogeny (Fig. 3B).
Elephant shark	*pkd2l1*	SINCAMG00000015409	Scaffold_3(+): 6986034-6999417	Unusual synteny, as is flanked by *abcg2*, which is adjacent to all *PKD2* genes examined in this study. However, it is also flanked by *mypn1, sirt1* and *dnajc12*, which flank green spotted pufferfish and medaka *pkd2l1* genes (data not shown). Phylogeny (Fig. 3B).
pkd2l2				
Spotted gar	*pkd2l2*	ENSLOCG00000011380	LG6(-): 32405994-32414168	Doesn't share synteny with mammalian *PKD2L2* genes. Phylogeny (Fig. 3B).

pkd2l2				
Elephant shark	*polycystic kidney disease 2*	SINCAMG00000005264	Scaffold_87(+): 2858280-2865700	Shares no synteny with mammalian and teleost *PKD2* genes. Does share synteny with mammalian *PKD2L2* genes (data not shown). Phylogeny (Fig. 3B).

Supplementary Table 5. Characterization of *pkd* genes in the genomes of holostei and cartilaginous fish.

Column one indicates the species. Column 2 lists gene name used in current Ensembl genome assembly (see materials and methods). Column 3 lists Ensembl gene ID. Column 4 shows position in current version of appropriate genome. Column 5 lists data that support the annotations shown here (see results for more info). *pkd* genes were first identified by performing textual searches of the appropriate genome assembly and their synteny and phylogeny examined (data not shown, Fig. 3A-B). We then searched for additional *pkd* genes that are present in either mammalian or teleost genomes, but had not been identified by our previous searches, by performing Tblastn analyses with the polycystin-cation-channel domain of the zebrafish Pkd protein, or the full-length sequence of the mouse PKD protein in question.

2 Supplementary References

Coxam, B., Sabine, A., Bower, N. I., Smith, K. A., Pichol-Thievend, C., Skoczylas, R., et al., (2014). Pkd1 regulates lymphatic vascular morphogenesis during development. *Cell Rep.* 7, 623-633. doi: 10.1016/j.celrep.2014.03.063
